# No Evidence for Pace of Life Evolution Along Elevational Gradients in Squamate Reptiles

**DOI:** 10.1111/ele.70343

**Published:** 2026-02-23

**Authors:** Tiberiu C. Sahlean, Ryan A. Martin

**Affiliations:** ^1^ Institute of Biology Bucharest Romanian Academy Bucharest Romania; ^2^ Department of Biology Case Western Reserve University Cleveland Ohio USA

**Keywords:** elevation gradient, latitude gradient, life‐history, pace of life syndrome, physiology, POLS, Reptilia, slow‐fast continuum, Squamata

## Abstract

Ecological conditions can significantly influence the trade‐off between survival, growth and reproduction, driving life‐history divergence among populations in different environments. The pace of life syndrome hypothesis (POLS) proposes that additional suites of traits, from physiology to behaviour, adaptively co‐evolve with life‐history traits along a slow‐fast continuum. Using data from 192 studies representing 104 squamate species, we performed phylogenetic meta‐analyses testing whether traits vary predictably across elevation within species in accordance with POLS. Results show there is no clear evidence for an overall intraspecific elevational pace‐of‐life syndrome in squamate reptiles. While high elevation populations had significantly lower body temperatures and larger egg sizes, most traits—including body size, longevity, fecundity and thermal tolerance—exhibited non‐significant elevational patterns. Critically, lizards and snakes responded fundamentally differently: snakes showed significant differences in adult female and neonate body size and fecundity across elevation, while lizards showed no significant divergence. Elevation‐latitude interactions provide further evidence against a single pace‐of‐life solution to elevation. Our findings challenge the applicability of POLS theory within species, revealing that life‐history evolution is more context‐dependent and taxonomically constrained than syndrome‐based approaches suggest. These results contribute to growing evidence that universal trait syndromes rarely emerge across environmental gradients in diverse taxonomic groups.

## Graphical abstract

Ecological conditions can significantly influence the trade‐off between survival, growth, and reproduction, leading to evolutionary divergence in life‐history traits among populations experiencing different environments. The pace of life syndrome hypothesis (POLS) proposes that additional suites of traits, from physiology to behaviour, adaptively co‐evolve with life‐history traits along this continuum. Using data from 192 studies representing 104 species, we find no clear evidence for an overall intraspecific elevational POLS in squamates, with most traits showing no significant patterns and a fundamental divergence between lizards (no response) and snakes (significant differences in adult female and neonate body size and fecundity). These findings challenge the broad applicability of POLS theory, suggesting that life‐history evolution is more context‐dependent and taxonomically constrained than universal syndrome‐based approaches predict.

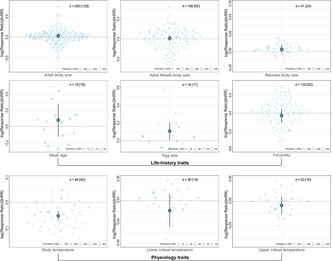

## 1 Introduction

Life‐history strategies can be arranged along a fast‐slow continuum (Stearns 1977; Promislow and Harvey 1990; Alice Boyle et al. 2016). This continuum emerges from the selection pressure to maximise the number of surviving offspring over an individual's lifetime, while simultaneously optimising growth and survival given limited resources (Stearns 1992; Hille and Cooper 2015). As these conditions cannot all be perfectly satisfied concurrently, this constraint is hypothesized to result in a co‐variation of life‐history traits across taxa called the pace‐of‐life (POL), which defines the fast‐slow continuum (Ricklefs 2000). Recent developments have extended this concept, initially to incorporate physiological (Ricklefs and Wikelski 2002; Wikelski et al. 2003; Martin II et al. 2006) and, later, behavioural traits (Réale et al. 2010), which co‐evolve with life‐history traits in a framework called the pace of life syndrome (POLS) hypothesis. According to POLS, suites of traits co‐vary across fast‐slow gradients, with a slow pace of life being characterised by small clutches, slow growth, long lifespan, and slow metabolism, while animals with a fast pace of life would have traits such as large clutches, fast growth, short lifespan, and fast metabolism.

To date, the POLS hypothesis has been tested for a variety of taxa, most comprehensively for birds (e.g., Hille and Cooper (2015), Wiersma et al. (2007), Wikelski et al. (2003)), mammals (e.g., Bielby et al. (2007), Promislow and Harvey (1990), Oli (2004)) and fish (e.g., Binder et al. (2016), Polverino et al. (2018), Turko et al. (2019)). Healy et al. (2019) concluded that a large proportion of variation in animal life history strategies can be explained by the pace of life, mortality risk and the number of breeding episodes throughout an individual's lifetime (i.e., degree of iteroparity). However, Dammhahn et al. (2018), based on a topical collection of meta‐analyses and reviews on POLS syndromes, as well as other integrative studies (Royauté et al. 2018; Mathot et al. 2019), emphasise that the empirical evidence for the POLS framework is mixed, especially at the within‐species level.

Variation in ecological conditions can mediate the trade‐off between survival, growth and reproduction, resulting in the evolution and divergence of traits across populations experiencing distinct environments (Mathot and Frankenhuis 2018), and, in the end, to an optimal pace of life solution. This hypothesis has been most prominently investigated in relation to latitude and, to a lesser extent, elevation (Hodkinson 2005; Hille and Cooper 2015). Spatial variation in latitude and elevation produces similar conditions (i.e., lower temperatures, greater seasonality, higher stochasticity, lower productivity, shorter breeding seasons) (Hodkinson 2005; Camfield et al. 2010; Hille and Cooper 2015; Alice Boyle et al. 2016; Balasubramaniam and Rotenberry 2016), but they also differ in several important aspects: high elevation environments are subject to increased solar insolation and lower atmospheric pressure (including partial pressure of gases), while maintaining the same photoperiod as the lowlands below (Körner 2007; Alice Boyle et al. 2016; Balasubramaniam and Rotenberry 2016). Moreover, elevational gradients are characterised by steep environmental changes over short horizontal distances (usually kilometres), which also leads to a decrease in land area (Körner 2007), while latitudinal gradients result in more gradual change over longer horizontal distances (usually hundreds of kilometres) (Hodkinson 2005) and do not necessarily imply a decrease in environmental space.

Understanding how predictably traits associated with pace of life respond to environmental gradients such as elevation can lead to more accurate predictions of organismal responses to current and future environmental change (Martin 1996; Buckley 2010; Alice Boyle et al. 2016; Martin et al. 2023). Indeed, elevational gradients are commonly used as a space‐for‐time substitution to better predict ecological and evolutionary consequences of climate change (Verheyen et al. 2019; Lovell et al. 2023). While organisms show widespread adaptive variation in phenotypic traits (especially life‐history traits) corresponding to changes in elevation (Keller et al. 2013), whether elevation consistently drives predictable and parallel pace‐of‐life evolution within species is largely unknown and has not been quantitatively tested for any taxa outside of birds (Alice Boyle et al. 2016; Balasubramaniam and Rotenberry 2016).

With a legacy of hundreds of millions of years of evolution, extant squamates are the most diverse clade of terrestrial vertebrates (Uetz et al. 2024). Squamates occupy most areas of the Earth, except polar regions and display a vast diversity of morphological and ecological forms (Vitt and Caldwell 2014). Thus, not surprisingly, they also exhibit a wide range of life histories and physiological and behavioural adaptations, shaped and evolved by the environments they live in (Shine 2005). Within squamates, the pace of life was investigated most comprehensively on the garter snake ( *Thamnophis elegans* ) (see Bronikowski and Arnold (1999), Bronikowski (2000), Bronikowski and Vleck (2010), Gangloff et al. (2017), Palacios et al. (2013)). Healy et al. (2019) also included reptile species from most living groups (chelonians, squamates and crocodilians) in their analysis of pace‐of‐life evolution. The adaptive impact of environmental factors on the phenotypic traits of reptiles has been a major focus of many studies, reviews and meta‐analyses, notably in relation to body size (Bergmann's rule) (Ashton and Feldman 2003; Angilletta et al. 2004; Ashton 2004; Sears and Angilletta Jr. 2004; Olalla‐Tárraga et al. 2006; Pincheira‐Donoso and Meiri 2013), life‐history traits (Sinervo 1990a; Rohr 1997; Sears and Angilletta Jr. 2003; Mesquita et al. 2016), and thermal physiology and behaviour (Sinervo 1990b; Garcia‐Porta et al. 2019; Muñoz and Bodensteiner 2019; Rivera‐Rea et al. 2023). Since single‐species studies lack generality, while single‐trait studies might overlook important factors driving variation (Alice Boyle et al. 2016), currently missing are integrative studies investigating the variation of traits within the POLS framework in squamates and the role environmental factors such as elevation play in shaping the pace of life (but also see Scharf et al. (2015)).

The goal of our study is to provide a quantitative synthesis testing intraspecific divergence and variation in squamate life history and physiology along the pace of life continuum in response to elevation. More precisely, we compare trait means and trait variation between conspecific populations occurring at different elevations, rather than comparing traits across species. We note that our approach tests whether individual POLS‐related traits diverge in the predicted direction along elevational gradients, rather than testing for the existence of POLS, which would require examining covariation among multiple traits (Réale et al. 2010; Dammhahn et al. 2018). Further, we also examine how these responses differ among squamate taxa (suborders, families). We broadly predict that, overall, populations of squamates from low elevations will exhibit traits corresponding to a fast pace of life, while populations from high elevations will conform to a slow pace of life, given the associated changes in environmental conditions and selection pressures with increasing elevation, specifically colder temperatures, shorter available activity times, and decreases in food availability and predation leading to convergent evolutionary (and/or plastic) responses (Adolph and Porter 1993; Shine 2005; Laiolo and Obeso 2017).

We also investigated how trait variability varied across elevation. Here, we predicted that populations living at higher elevations will show increased trait variation overall compared to lower elevations, because of the reduced availability and greater spatio‐temporal variation in resources (e.g., in the thermal environment or in food resources), resulting in greater variation in physiology and growth among individuals (Shine 2005). For the traits with data available to test our predictions, we expected populations from higher elevation to show higher heterogeneity in body size and reproductive output. In contrast however, we predicted higher elevation populations to be more precise thermoregulators due to lower thermal quality of their environments, leading to a decreased variation in body temperature at higher elevations (Blouin‐Demers and Nadeau 2005; Lourdais et al. 2013; Lymburner and Blouin‐Demers 2020).

## 2 Methods

### 2.1 Data Collection

We conducted an extensive literature search up to the end of 2024 for empirical field studies reporting trait data (life‐history or physiology) for two or more populations of the same species located at different elevations. One person (TCS) was responsible for the entire process, from performing the search to constructing the final database of articles used for the meta‐analyses. The search was conducted on Google Scholar and Scopus using multiple combinations of search terms (see Appendix S3, Table S1). First we looked at the title and abstract to see if it matched our area of interest in any way, then quickly continued to the methodology section to check if at least two populations located at different elevations were used, and then we looked for any type of summary data or Supporting Information; material from which we could extract information. Google Scholar does not allow the bulk download of references so in this case the entire process was performed online. We avoided the inconsistent search results displayed in Google Scholar by having one person conduct the entire search procedure and using browser bookmarks to continue with the results from the same search. We collected peer‐reviewed articles published in English which contained relevant data, based on a rigorous protocol detailed in Appendix S3—Supporting Information; methods. We started with 1916 articles and in the end we extracted data from 192. When faced with multiple populations or summary statistics divided into different categories we used statistical techniques for pooling data (*N* = 131 effect sizes) (see Appendix S1—dataPooling column, Appendix S3—Supporting Information; methods). We also extracted data from figures and, in some cases, specifically when we lacked one study to include a family in a meta‐regression, we e‐mailed authors of the primary papers (we contacted the authors of four papers and received one reply with three effect sizes for *Vipera ammodytes* ).

Upon finalising the database, we assigned traits to several categories: (1) adult body size, composed of snout‐vent length (SVL) and mass of adults, (2) adult female body size, composed of SVL of adult females, irrespective of reproductive status, (3) neonate body size, composed of SVL and mass of neonate individuals (not juveniles), from either oviparous or viviparous species, born in nature, or from clutches incubated to mimic the natural environment of the focal population, (4) body temperature (*T*_b_), comprising measurements taken in the field, (5) lower critical temperature (CT_min_), measuring the critical thermal minimum, (6) upper critical temperature (CT_max_), measuring the critical thermal maximum, (7) egg size, comprising measurements of egg volume, egg mass, egg length and egg width, (8) mean age, comprising the estimated mean age of individuals obtained from growth rings and (9) fecundity, comprising measurements of clutch size, clutch mass, clutch weight, mean number of live born, mean litter mass and relative clutch mass (RCM) (Appendix S2).

### 2.2 Effect Size Calculation

Trait categories collected varied in their unit of measure, so we chose the response ratio (or ratio of means) (*lnRR*) as our effect size metric (Hedges et al. 1999) for the analysis of mean differences between low and high altitude populations. The response ratio has been widely used in ecological studies since its introduction (Lajeunesse 2015) and, similar to the standardised mean difference (SMD), it is unitless, thus allowing for combining different outcomes (Friedrich et al. 2008). Unlike SMD, though, the response ratio has no indication of the magnitude of the effect, because it is calculated as the natural logarithm of the ratios between the two means (Nakagawa et al. 2015), which can be beneficial in cases when there is no knowledge regarding the expected magnitude of change. However, a sense of magnitude can be obtained by converting the result to a percentage change using the formula, where Ψ is the effect size (Pustejovsky 2018). Since the result is symmetric around 0, in our analyses negative values indicate a change towards low elevation populations, while positive values indicate a greater response in high elevation populations.

For the analysis of variation of traits between low and high elevation populations, we used the coefficient of variation (*lnCVR*) as our effect size statistic (Nakagawa et al. 2015), which is calculated as the natural logarithm of the ratio between the coefficients of variation from two groups and does not necessitate any additional data beyond what is used for the analysis of response ratios. Moreover, the coefficient of variation is most useful in systems where a mean–variance relationship (such as Taylor's law) exists (Senior et al. 2020) such as populations in higher elevation environments where total area decreases. Since the results are also symmetric around zero, we changed the sign of the results to match the meta‐analysis of response ratios: negative values signify greater variation of traits in low elevation populations, while positive values signify greater variation in high elevation populations.

Effect sizes were computed using the *escalc* function in the *metafor* package (Viechtbauer 2010). We calculated a separate effect size for each summary statistic when multiple summaries were derived for the same population (e.g., body mass and snout‐vent length) to maximise the amount of data available (Noble et al. 2017).

### 2.3 Meta‐Analytical Methods

Meta‐analyses and meta‐regressions were performed in R v4.4.1 (R Core Team 2022) using the *metafor* package with a restricted maximum likelihood estimator (REML). Datasets for meta‐analyses and meta‐regressions, both for the mean effect size and the coefficient of variation, were selected based on a set of rules detailed in the Supporting Information; methods. We used three distinct model types to address our research questions regarding mean effect and variation of traits: (Adolph and Porter 1993) overall meta‐analyses, which we used to test for intraspecific differences in mean and variability of squamate traits between low and high elevation populations (Agrawal 2020) categorical moderator meta‐regressions, where we compare intraspecific differences in trait response to elevation in higher taxonomic units, using suborder or family as categorical moderators, and (Alice Boyle et al. 2016) continuous moderator meta‐regressions, which tests the influence of elevational and latitudinal factors in determining the observed intraspecific differences.

We used multi‐level models to deal with shared measurements and phylogenetic relationships between species, and random factors were included based on their ability to explain the total amount of heterogeneity (*I* ^*2*^: representing the total relative within‐and‐between study variance) (factors explaining < 1% of heterogeneity were dropped), calculated for multi‐level models using the *orchard 2.0* package (Nakagawa, Lagisz, et al. 2023). Phylogeny was controlled using the most recent phylogenetic subset downloaded from VertLife (https://vertlife.org) and the procedure detailed in Supporting Information; methods, as described by Noble et al. (2017), Nakagawa et al. (2022), and Nakagawa, Yang, et al. (2023).

Models used the following structure for overall meta‐analyses $z_{i}=\beta_{0}+u_{\text{effect}\left[i\right]}+u_{\text{study}\left[i\right]}+u_{\text{species}\left[i\right]}+u_{\text{phylogeny}\left[i\right]}+u_{\text{location}\left[i\right]}+m_{i}$, where *z*_*i*_ is the effect size for the observation *i*, *β*_*0*_ is the overall mean effect size (or intercept), *u*_effect_ to *u*_location_ are the random effects, and *m*_*i*_ is the sampling error.

For the meta‐regressions with categorical moderators, the model structure was the following for suborders and families: $\begin{array}{c} z_{i}=\beta_{1}X_{1\left[i\right]}+\ldots +\beta_{k}X_{k\left[i\right]}+u_{\text{effect}\left[i\right]}+u_{\text{study}\left[i\right]}+u_{\text{species}\left[i\right]}+ \end{array}$ $\begin{array}{c} u_{\text{phylogeny}\left[i\right]}+u_{\text{location}\left[i\right]}+m_{i} \end{array}$, where *β*_*1*_ to *β*_*x*_ are the mean effects corresponding to dummy variables *X*_*1*_ to *X*_*k*_ for each family or suborder; the categorical models did not have an intercept as we were interested in differences in higher taxonomic levels not comparisons to a reference level.

For meta‐regressions with continuous moderators model assumed the following structure: $\begin{array}{c} z_{i}=\beta_{0}+\beta_{1}X_{1}+\ldots +\beta_{k}X_{k}+ \end{array}$ $\begin{array}{c} +u_{\text{effect}\left[i\right]}+u_{\text{study}\left[i\right]}+u_{\text{species}\left[i\right]}+u_{\text{phylogeny}\left[i\right]}+u_{\text{location}\left[i\right]}+m_{i} \end{array}$, where *β*_*1*_ to *β*_*k*_ are the regression coefficients for the variables *X*_*1*_ to *X*_*k*_. We note that the inclusion of the random factors in the final models was based on the amount of heterogeneity explained (see above and Tables 1 and 3).

**TABLE 1:** Mean response ratios (*β*_0_) modelled for each trait using multi‐level meta‐analyses without moderators. For each trait, the number of studies, number of effect sizes (*k*), standard errors (SE), 95% confidence intervals, significance (*p*) and heterogeneity (*I* ^*2*^) are summarised. In cells lacking *I* ^*2*^ values, the corresponding random factor was not included in the model based on the percentage of heterogeneity explained (see Methods section).

	Trait								
	Adult body size	Adult female body size	Neonate body size	Egg size	Fecundity	Mean age	*T*_b_	CT_min_	CT_max_
*N*_studies_	129	93	26	11	63	18	40	14	20
*k*	250	106	45	16	130	18	46	26	34
Initial prediction	↑ with elevation	↑ with elevation	↑ with elevation	↑ with elevation	↓ with elevation	↑ with elevation	↓ with elevation	↓ with elevation	↓ with elevation
*β*_0_	0.030	0.006	0.033	0.106	−0.053	0.077	−0.052	−0.115	−0.006
SE	0.029	0.013	0.024	0.047	0.076	0.098	0.009	0.093	0.008
*p*	0.306	0.671	0.188	**0.038**	0.489	0.441	**< 0.001**	0.228	0.462
Lower CI	−0.028	−0.021	−0.017	0.007	−0.206	−0.129	−0.070	−0.307	−0.022
Upper CI	0.089	0.032	0.083	0.206	0.100	0.284	−0.034	0.077	0.010
*I* ^*2*^_effect_ (%)	35.48	—	57.99	98.15	36.8	11.2	—	—	—
*I* ^*2*^_study_ (%)	—	5.76	20.52	—	—	11.2	66.1	—	—
*I* ^*2*^_species_ (%)	11.76	5.3	—	—	—	11.2	3.49	36.1	80.7
*I* ^*2*^_phylo_ (%)	—	2.31	—	—	26.04	46.6	—	57.8	14.9
*I* ^*2*^_location_ (%)	47.41	84.37	20.52	—	35.17	11.2	27.81	—	—
*I* ^*2*^_total_ (%)	99.78	97.73	99.04	98.15	98.02	91.2	97.41	94.18	95.6

We tested for the impact of shared measurements using a variance–covariance matrix as suggested by Nakagawa, Yang, et al. 2023 and continued the analyses with the original values based on the results (see methods).

### 2.4 Moderating Variables

In our meta‐regression models, we used elevation range (continuous), latitudinal difference (continuous), mean latitude (continuous), suborder (categorical) and family (categorical) as moderators (analogous to predictor variables in linear models) to test different hypotheses. Meta‐regressions were produced when there were at least four studies for each family and a minimum of 20 total effect sizes (Fu et al. 2011), and to keep results consistent, we applied the same criteria for all meta‐regressions, independent of moderator type. The trait categories that fit our criteria were adult body size, body temperature, adult female body size, neonate body size, and fecundity for mean effects, and adult body size, body temperature, adult female body size, and fecundity for the coefficient of variation.

Data regarding the elevation of the populations was extracted directly from the study (if available); otherwise, we used geographic coordinates or georeferencing to extract the data (see Supporting Information; Methods). Elevation of the population pairs was used to calculate elevation range, which we then log‐transformed to improve normality and included as a moderator to test if an increased separation corresponds to an increase in trait differentiation.

Because our database included all multi‐population studies which contained relevant data, it also meant that some populations had large latitudinal spacing which could create a bias in the results. We accounted for the effect of latitudinal differences between populations and between studies using two variables: (1) the log‐transformed absolute value of the latitude difference between low and high elevation populations, and (2) the absolute value of the mean latitude of the population pairs (see Supporting Information; Methods).

### 2.5 Publication Bias

We performed publication bias tests in the form of funnel plots and meta‐regressions aimed at detecting (a) a small study effect, which refers to cases where effect sizes based on small sample sizes tend to be larger (using Egger's regression adapted for multilevel models) or (b) a time‐lag bias or decline effect, where positive results are published earlier than negative results (Nakagawa et al. 2022; Nakagawa, Yang, et al. 2023). We performed publication bias tests for traits where meta‐regressions were also performed (see Supporting Information; methods). We detected a time‐lag bias for response ratios of neonate body size in both single moderator meta‐regression (*t* = −2.379, *p* = 0.0265) and the all‐in multilevel meta‐regression (*t* = −2.2342, *p* = 0.0365). Therefore, the results presented and discussed for neonate body size are the bias‐corrected values of the intercept (*β*_0_) (Nakagawa, Yang, et al. 2023).

## 3 Results

Our final database used for the meta‐analyses consisted of 671 effect sizes extracted from 192 studies, comprising 104 species of squamates from 10 families (Appendix S2). Families Lacertidae and Phrynosomatidae had the greatest number of species (33 and 18 species respectively), while for Anguidae and Phyllodactylidae we only had one representative each. We collected data for six life‐history traits—*adult body size*, 250 effect sizes extracted from 140 studies, *adult female body size*, 106 effect sizes extracted from 93 studies, *neonate body size*, 45 effect sizes extracted from 26 studies, *egg size*, 16 effect sizes from 11 studies, *mean age*, 18 effect sizes from 18 studies, *fecundity*, 130 effect sizes from 63 studies and three physiological traits—*body temperature* (*T*_b_), 46 effect sizes from 40 studies, *lower critical temperature* (CT_min_), 26 effect sizes from 14 studies and *upper critical temperature* (CT_max_), 35 effect sizes from 21 studies. Coordinate pairs were available from around the world, including North, Central and South America, Eurasia, Australia and Africa, but from the latter we recovered the least amount of data (Appendix S3, Figure S1). The median difference in elevation between population pairs was 918 m (range 100–3500 m) and the highest elevation population was located at 4583 m.

### 3.1 Trait Divergence Across the Elevational Gradient

#### 3.1.1 Overall Effect Size

**FIGURE 1:**
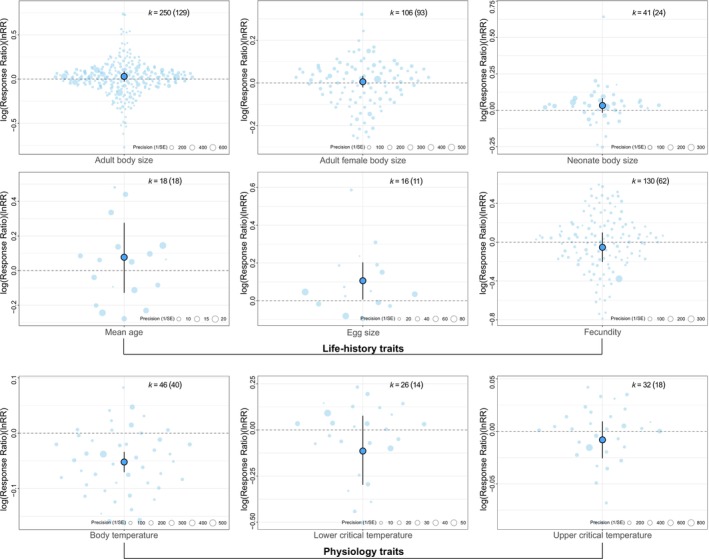
Summary of mean responses (log response ratio, *lnRR*) and 95% confidence intervals for life‐history and physiological traits of squamates in response to elevational differences. Individual effect sizes are displayed as points scaled by precision (1/SE), where larger points indicate more precise estimates. The mean effect is shown as the central, outlined point and the black whiskers provide the 95% CI For each trait, *k* indicates the number of effect sizes, with the number of studies in parentheses.

Overall, our results do not show patterns of intraspecific trait divergence consistent with a POLS within squamate species in response to elevation. With few exceptions, effect sizes were small and non‐significant (Figure 1). Egg size increased significantly towards higher elevations by an average of 11% [CI: 0.7, 22.87] as predicted, while body temperature declined with elevation by an average of 5.3% [CI: 3.45, 7.25], also matching our expectations. On the other hand, we did not find significant differences between low and high elevation populations in adult body size (mean: 3.04% [CI: −2.83, 9.3]), adult female body size (mean: 0.6% [CI: −2.12, 3.25]), neonate body size (mean: 3.35% [CI: −1.71, 8.65]), fecundity (mean: −5.44% [CI: −22.87, 10.51]), mean age (mean: 8% [CI: −13.76, 32.84]), lower critical temperature (mean: −12.18% [CI: −35.93, 7.64]) or upper critical temperature (mean: −0.6% [CI: −2.22, 1]).

Heterogeneity (i.e., *relative within‐and‐between study variance*) was very high in all intercept models with *I* ^*2*^ values above 90%, indicative of a high degree of unexplained variation. Heterogeneity was a result of differences in measurements (*I* ^2^_effect_), methodological differences (*I* ^2^_study_), geographical location of the studies (*I* ^2^_location_), and in some cases variability as a result of phylogenetic relatedness (*I* ^2^_phylo_) or differences between species (*I* ^2^_species_) (Table 1).

#### 3.1.2 Divergence Among Suborders and Families

We found some divergence in trait responses between the two suborders (Lacertilia and Serpentes) and for some families, but differences were generally small and non‐significant. At the suborder level, only snakes showed significant differences between low and high elevation populations with increased adult female body sizes (mean: −6.82% [CI: −13.31, −0.7]) and fecundity (mean: −67.02% [CI: −110.22, −32.57]) at lower elevations, and bigger neonate body sizes at higher elevations (mean: 14.91% [CI: 2.12, 29.3]) (Appendix S3, Table S3, Figures S5 and S6, S8). However, we point out the sample sizes for snakes, which are considerably smaller for snakes than for lizards (Appendix S3, Figures S5 and S6, S8), therefore increasing the probability of false positives. The pattern identified for suborders was mostly mirrored at the family level. Notably, neonate body size increased significantly in high elevation populations of colubrids (mean: 14.56% [CI: 1.81, 28.91]), while fecundity was significantly reduced (mean: −66.36% [CI: −113.4, −29.69]) (Appendix S3, Table S2, Figures S6 and S7). Additionally, body temperature was significantly lower in high elevation populations of Anolidae (mean: −9.85% [CI: −16.41, −3.66]) and Phrynosomatidae (mean: −5.75% [CI: −10.18, −1.51]) (Appendix S3, Table S2, Figure S9).

#### 3.1.3 Effect of Moderators

Elevation range was not a significant moderator of body size differences in any of the life stages analysed (adults, neonates), but differences in adult female body size were positively and significantly associated with increased latitude differences (Table 2). Neonate body size was also significantly moderated by mean latitude (increasing divergence at higher mean latitude), but only in analyses with a non‐significant interaction with elevation range, and not in the single moderator model (Appendix S3, Table S8).

**TABLE 2:** Summary of the moderators explaining the influence of elevation range (elvR) and latitude difference (latD) on the selected mean life‐history and physiological traits.

Moderator	Adult body size			Adult female body size			Neonate body size		
	Est	SE	*p*	Est	SE	*p*	Est	SE	*p*
elvR	−0.013	0.028	0.659	−0.006	0.017	0.727	0.002	0.043	0.959
latD	0.017	0.011	0.122	0.021	0.007	**0.005**	−0.002	0.017	0.904
elvR × latD	−0.011	0.011	0.357	−0.002	0.008	0.808	−0.012	0.022	0.590

Moderator	Fecundity			T_b_		
	Est	SE	*p*	Est	SE	*p*
elvR	−0.123	0.047	**0.012**	−0.049	0.022	**0.032**
latD	0.019	0.019	0.333	0.007	0.006	0.241
elvR × latD	−0.039	0.019	**0.049**	−0.005	0.008	0.526

We found a significant interaction between elevational range and latitudinal difference on differences in fecundity (Table 2). Over much of the parameter space, low elevation populations tended to have greater fecundity than high elevation populations, and this effect increased with increasing elevational range. In contrast, when populations were separated by large latitudinal differences, higher elevation populations tended to have greater fecundity (Appendix S3, Figure S10). However, there were few studies where populations were separated by large differences in latitude (Appendix S3, Figure S11), and so the biological interpretation of this interaction should be treated with some caution.

We then dropped the interaction to examine a reduced model with latitude difference and elevation range as main effects. In this model, elevation was marginally non‐significant (*β* = −0.082, SE = 0.041, *t* = −1.991, *p* = 0.052) with a trend of increasingly greater fecundity for low elevations when controlling for latitudinal differences. Latitudinal difference, in contrast, was significant, with higher elevation populations showing increased fecundity with increasing latitudinal differences (*β* = 0.037, SE = 0.017, *t* = 2.181, *p* = 0.034). Overall, and keeping in mind the smaller number of studies with large latitudinal extents, this suggests that latitude might moderate divergence in fecundity across elevation.

The body temperature difference between low and high‐altitude populations was significantly and negatively influenced by the difference in elevation (Table 2) in analyses with latitude difference but interacted with mean latitude of the population pair when we included it as a moderator (Appendix S3, Table S8). Body temperatures are greater for low elevation populations across almost all combinations of elevational range and mean latitude, whereas higher body temperatures for high elevation populations are only found at high values of mean latitude combined with small elevational differences between low and high populations (Appendix S3, Figure S12).

### 3.2 Trait Variation Along the Elevational Gradient

#### 3.2.1 Overall Effect Size

**FIGURE 2:**
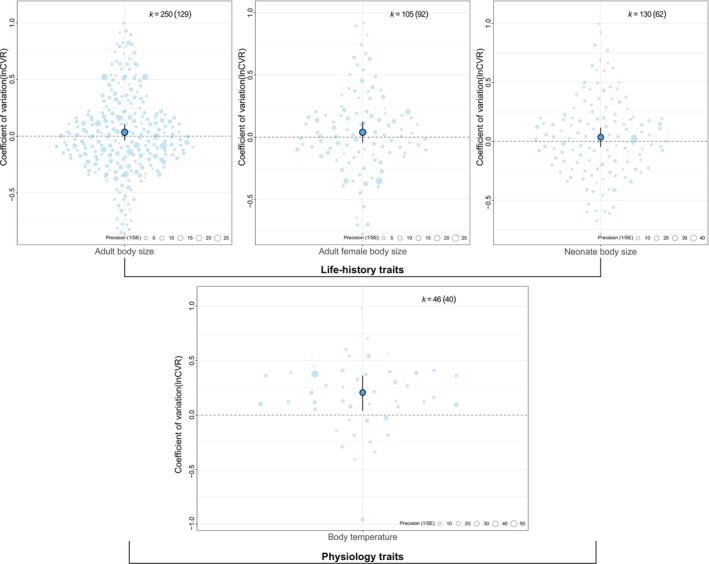
Summary of mean variation (coefficient of variation, *lnCVR*) and 95% confidence intervals for life‐history and physiological traits of squamates in response to elevational differences. Individual effect sizes are displayed as points scaled by precision (1/SE), where larger points indicate more precise estimates. The mean effect is shown as the central, outlined point and the black whiskers provide the 95% CI For each trait, *k* indicates the number of effect sizes, with the number of studies in parentheses.

The results on variation of life‐history and physiology traits completely contradicted our predictions. Body temperature variation was significantly higher in high elevation populations of squamates, on average by 23.24% [CI: 3.97, 46.08] (Figure 2, Table 3), contrary to our expectation of more precise thermoregulators at higher elevations. Moreover, also opposed to our expectations, there was no significant variation across elevational gradients in adult body size (mean: 3.45% [CI: −3.77, 11.18]), adult female body size (mean: 3.97% [CI: −4.5, 12.97]), or fecundity (mean: 3.66% [CI: −4.7, 12.52]) (Figure 2, Table 3).

**TABLE 3:** The coefficient of variation (*β*_0_) modelled for each trait using multi‐level meta‐analyses without moderators. For each trait, the number of studies, number of effect sizes (*k*), standard errors (SE), 95% confidence intervals and significance (*p*) and heterogeneity (*I* ^*2*^) are summarised. In cells lacking *I* ^*2*^ values, the corresponding random factor was not included in the model based on the percentage of heterogeneity explained (see Methods section).

	Trait			
	Adult body size	Adult female body size	Fecundity	*T*_b_
*N*_studies_	129	85	63	40
*k*	250	89	130	46
Initial prediction	↑ variation at higher elevations	↑ variation at higher elevations	↑ variation at higher elevations	↓ variation at high elevations
β_0_	0.034	0.039	0.036	0.209
SE	0.036	0.041	0.041	0.084
*p*	0.343	0.346	0.382	**0.017**
Lower CI	−0.037	−0.044	−0.046	0.039
Upper CI	0.106	0.122	0.118	0.379
*I* ^2^_effect_ (%)	40.5	—	55.31	—
*I* ^2^_study_ (%)	16.0	31.9	—	91.59
*I* ^2^_species_ (%)	—	10.3	—	—
*I* ^2^_location_ (%)	30.5	29.5	23.35	6.02
*I* ^2^_total_ (%)	87.1	71.8	78.67	97.62

Heterogeneity was high in the intercept models for adult body size (*I* ^2^_total_ = 87.1%), fecundity (*I* ^2^_tota*l*_ = 78.67%) and body temperature (*I* ^2^_total_ = 97.62%) and moderate for adult female body size (*I* ^2^_total_ = 71.8%) (Table 3). Variability came mainly from differences in measurements (*I* ^2^_effect_), the methodology used for the studies (*I* ^2^_study_), the location of the studies (*I* ^2^_location_), or was the result of differences between species (*I* ^2^_species_) (Table 3).

#### 3.2.2 Divergence Among Suborders and Families

There were no differences between low and high elevation populations in the two suborders (Appendix S3, Table S5). There was also no significant variation between low and high elevation populations in families for most of the traits analysed, with the exception of adult female body size, where viper populations from higher elevations showed significantly more variation compared to low elevations (mean: 63.88% [CI: 3.97, 158.05]) (Appendix S3, Table S4, Figure S4).

#### 3.2.3 Effect of Moderators

Elevation difference played no significant role in predicting the variation of life‐history or physiology traits (Table 4), nor did latitude difference or mean latitude of the population pairs (Table 4, Appendix S3, Table S9).

**TABLE 4:** Summary of the moderators explaining the influence of elevation range (elvR) and latitude difference (latD) on the variation of life‐history and physiological traits.

Moderator	Adult body size			Adult female body size		
	Est	SE	*p*	Est	SE	*p*
elvR	−0.006	0.058	0.915	−0.023	0.060	0.705
latD	−0.007	0.022	0.744	−0.007	0.028	0.795
elvR × latD	0.002	0.024	0.919	−0.023	0.028	0.414

Moderator	Fecundity			*T*_b_		
	Est	SE	*p*	Est	SE	*p*
elvR	0.058	0.064	0.364	0.075	0.199	0.707
latD	0.035	0.028	0.219	−0.054	0.063	0.405
elvR × latD	0.017	0.029	0.567	0.024	0.061	0.696

## 4 Discussion

Our comprehensive synthesis found no clear evidence for intraspecific elevational trait divergence in life history or physiology consistent with the existence of an overall pace of life syndrome in squamate reptiles. Despite analysing data from 192 studies encompassing 104 species and 9 life‐history and physiological trait categories, we found consistently small and non‐significant effect sizes. This preponderance of negative results challenges the overall applicability of POLS theory within species in the context of environmental gradients.

This is the first comprehensive test of whether individual traits within squamate species diverge along elevational gradients in directions predicted by POLS, providing the first such data for squamates. These negative findings align with evidence that the detectability of POLS‐related trait shifts in response to environmental gradients varies across contexts. While our study tested for trait divergence in the direction predicted by POLS theory (e.g., slower pace of life at higher elevation) and not trait covariation per se, the lack of consistent directional divergence in individual traits suggests that elevation does not reliably generate the selection pressures assumed to drive POLS‐related trait shifts within species. Recent meta‐analyses across various taxa and environmental gradients have similarly shown that the detectability of POLS patterns is highly context dependent, varying with factors such as environmental conditions, sex or species studied (Santostefano et al. 2017; Royauté et al. 2018; Tarka et al. 2018). Our results thus contribute to understanding the conditions under which environmental gradients drive life‐history divergence and highlight the need for more nuanced approaches to understanding life‐history evolution. Indeed, there are scant formal models for predicting trait covariation under POLS (Mathot and Frankenhuis 2018; Laskowski et al. 2021) and there is ambiguity in which traits co‐vary as part of a syndrome or if composite variables should be considered (Araya‐Ajoy et al. 2018; Mathot and Frankenhuis 2018; Royauté et al. 2018; Gopal et al. 2023). Our own synthesis tested predictions derived from a verbal formulation of a POLS hypothesis based on the trade‐offs expected due to varying environmental conditions generated by the elevational gradient.

There are several potential reasons for the apparent lack of a unified elevational POLS within squamate reptile populations that likely reflect the extraordinary ecological and evolutionary diversity within this group. Although squamates are monophyletic and thus share a common evolutionary history, they exhibit a tremendous diversity of shape, function, and ecology that could yield disparate solutions to the shared challenge of adapting to elevation. Indeed, our suborder‐level analyses reveal that lizards and snakes respond fundamentally differently to elevation: snakes showed significant differences in adult female body size, neonate body size, and fecundity between low and high elevation populations, while lizards showed minimal and non‐significant responses across all life‐history traits. This divergence likely reflects the greater ecological and morphological disparity among lizards compared to snakes. Lizards have a great diversity of body shapes and diets, ranging from carnivory to herbivory, even within families, and some groups show increased thermal plasticity through behavioural thermoregulation (Labra et al. 2009; Muñoz and Losos 2018; Refsnider et al. 2018; Plasman et al. 2025). In contrast, snakes are limited in body shape, feeding ecology (all known species are predators) and rely on adjusting the timing of activity for coping with thermal environments (Weatherhead et al. 2012). Shine (1980) suggests that in lizards, reducing current fecundity for future gains in body size offers little benefit, as survivorship is likely the main reproductive cost. In snakes, on the other hand, adult female body size may decrease at higher elevations through a combination of reduced productivity (e.g., Alice Boyle et al. (2016)), restricted trophic breadth and reduced female mobility during pregnancy (Seigel et al. 1987).

Second, environmental complexity might moderate trait covariation. Multivariate environmental variation is often cited as a potential moderator of trait co‐variation, as variation in ecological conditions can induce or mask changes in morphology, life‐history, or physiology, with distinct effects across taxa and life stages (Hämäläinen et al. 2021). While elevation itself does not influence the life‐history and physiology of animals per se, it acts as a proxy for an aggregate of factors (such as temperature, seasonality, precipitation quantity and type, primary productivity, habitat size, predation risk) which affect ectotherms and endotherms differently (reviewed extensively in Alice Boyle et al. (2016), Hille and Cooper (2015), Hodkinson (2005) and Laiolo and Obeso (2017)). This environmental complexity means that elevation creates multiple, potentially conflicting selection pressures rather than a single coherent selective environment that would favour directional trait evolution.

Our results demonstrate this complexity empirically. The elevation‐latitude interaction we detected for fecundity exemplifies how multiple environmental gradients can interact. Our prediction that populations from lower elevations would exhibit greater fecundity, characteristic of a fast pace of life, did not match our results—with the exception of snakes. Although there was a trend towards increased fecundity at lower elevations, the effect was minimal and non‐significant. Instead, fecundity appears to be shaped by an interaction between elevational and latitudinal distance between populations. Similarly, divergence in body temperature was influenced by an interaction between elevational difference and mean latitude. That patterns of trait divergence associated with elevation may interact with latitude is perhaps not surprising as seasonality and annual temperature variation are much greater at higher compared to lower latitudes (Janzen 1967; Ghalambor et al. 2006). Moreover, these non‐linear patterns suggest regional adaptation, consistent with the interpretation that large‐scale ecological variation promotes the evolution of locally adapted traits rather than universal elevational response (Wang and Bradburd 2014).

Gene flow, especially along steep elevational gradients, could also constrain local adaptation (Bachmann et al. (2020); reviewed in Lenormand (2002)). Indeed, Bachmann et al. (2020) found that adaptive divergence in developmental rates in anurans was much weaker—and conversely local maladaptation was stronger—across steeper elevational clines compared to shallower latitudinal clines of comparable magnitude. Our results also show a lack of intraspecific divergence in thermal tolerance traits (CT_min_ and CT_max_), which is consistent with the pattern of gene flow constraining local adaptation. However, recent research (Lange et al. 2025) found that lower and upper critical thermal temperatures predict the cold and warm distribution limits of lizards in the genus *Anolis* along elevational gradients in the Caribbean. This contrast suggests that while there is a thermal physiology‐environment relationship at the species level, which is not subject to the homogenising effect of gene flow, this pattern will not be detected in intraspecific comparisons due to ongoing gene flow between populations at different elevations which prevents local adaptation.

Behaviour could also act to shield morphological and life history traits from selection in response to elevation (i.e., the Bogert effect Huey et al. (2003); Muñoz (2022)). For example, active thermoregulatory behaviour—especially widespread in lizards—could forestall evolutionary divergence of traits such as thermal tolerance across elevations (Huey et al. 2003; Muñoz and Bodensteiner 2019). Parental care, such as nest‐site selection (Shine and Harlow 1993; Shine and Harlow 1996; Telemeco et al. 2009) or maternal basking behaviour (Shine and Harlow 1993) could similarly influence their offspring's early environment and partially shield their phenotypes from selection (Diamond and Martin 2021).

Additionally, variation in resource acquisition, an important driver of life‐history evolution, but one which we did not account for in our analysis due to lack of data, could further obscure POLS patterns across elevation. Resource availability and competition for resources differ dramatically between elevations because lower elevations support higher productivity but also exhibit higher levels of competition (Morin and Chuine 2006; McCain 2010; Alice Boyle et al. 2016). If increased resource acquisition at low elevations is offset by intensified competition and, inversely, reduced competition at high elevations compensates for lower resource availability, this could result in shifts of life‐history strategies, masking or counteracting POLS predictions. Indeed, as variation of life‐history traits is fundamentally linked to resource acquisition rate (van Noordwijk and de Jong 1986), the lack of information regarding resource dynamics across the elevation gradient may contribute to the absence of a detectable syndrome in our analysis.

There were a few traits with responses that significantly differed across elevation both in support of and in contrast to our prior predictions. First, body temperature responses matched our predictions, with all families for which we had data (all lizards) exhibiting lower body temperatures at higher elevations and divergence increasing with greater elevational differences. This pattern recalls the Thermal Behaviour Syndrome (TBS) hypothesis proposed by Goulet et al. (2017), which argues that, given the strong and positive relationship between metabolism and temperature, ectotherms would be situated along a cold‐hot continuum, where individuals would select and perform better at low body temperatures (cold types) or at high body temperatures (hot types) (Goulet et al. 2017). Recent empirical work by Michelangeli et al. (2018) found evidence for a thermal‐behavioural syndrome in delicate skinks ( *Lampropholis delicata* ) and comprehensively demonstrated that thermal type also predicted habitat use, with ‘hot’ types spending more time in warmer, more exposed microhabitats, suggesting that thermal physiology could drive ecological niche partitioning within populations. Unfortunately, our dataset lacks behavioural or metabolic data needed to test this hypothesis. However, the potential for within‐population thermal divergence warrants further investigation, as individuals with different thermal types may respond distinctively to elevational selection pressures. The emergence of thermal patterns despite the absence of life‐history POLS reinforces the argument that the lack of syndrome‐level covariation reflects genuine biological differences rather than methodological limitations.

Our expectations regarding body temperature variation were that squamates would be precise thermoregulators (Díaz and Cabezas‐Díaz 2004; Blouin‐Demers and Nadeau 2005), therefore showing a reduced level of variability at higher elevations, owing to the strong dependence of ectotherm performance with body temperature (Huey and Kingsolver 1993). Instead, our results show that the mean variation of body temperature increases at higher elevations, which would suggest there are increased costs to thermoregulation at higher elevations (Huey and Slatkin 1976), probably related to reduced basking opportunities and the heterogenous and cooler nature of high‐altitude habitats. Alternatively, some squamate species can shift between thermoregulation and thermoconforming depending on ecological conditions (Hertz et al. 1993; Dubiner et al. 2024) and this behaviour flexibility could contribute to increased body temperature variation at high elevations. However, since many factors that could lead to increased variation were not controlled for in this body temperature data (e.g., season, time of day, sex, age, degree of behavioural thermoregulation), we caution that this result should not be overgeneralized. Moreover, increased variation in body temperature could instead be due to the evolution of wider thermal performance curves at higher elevations (Tüzün and Stoks 2018).

Lastly, as predicted, we did find overall support for increased egg size at higher elevations, and similarly an increase in neonate size within snakes at higher elevations. These results, together with the significant declines of fecundity at high versus low elevation in snakes, suggest a trade‐off between offspring investment and fecundity. Several factors may explain this pattern. First, elevation may alter age specific mortality—environments with low adult mortality relative to juvenile mortality are expected to favour larger eggs and smaller clutches and vice versa (Stearns 1992). The colder, shorter growing seasons and lower food availability of high elevations along with higher predation at lower elevations could drive patterns of higher juvenile vs. adult mortality at high elevations and higher adult vs. juvenile mortality at low elevations (Hille and Cooper 2015; Laiolo and Obeso 2017). Supporting this, longevity tended to increase with elevation, although not significantly so. Second, selection for viviparity could also lead to reduced clutch sizes, larger eggs and larger neonates at higher elevations. Viviparous species and populations are more common at higher elevations than oviparous ones, and high elevation oviparous populations tend to retain eggs longer before laying than low elevation populations (Mathies and Andrews 1995; Rodríguez‐Díaz and Braña 2012). The advantages of viviparity and longer egg retention are likely due to the benefits of maternal thermoregulation for embryo development in thermally challenging environments (Packard et al. 1977; Tinkle and Gibbons 1977; Shine 1985). In turn, longer egg retention increases clutch mass via water retention (Vleck 1991), which may favour smaller clutches, reducing negative effects on female mobility (Sinervo et al. 1991). Of course, despite these significant patterns, empirical counter examples exist, with the reverse trade‐off occurring across elevation (e.g., Sinervo (1990a)).

The preponderance of negative results has important theoretical implications that extend beyond squamate biology. The absence of intraspecific elevational POLS challenges fundamental assumptions about how predictable environmental gradients should generate predictable trait syndromes within species. Some authors argue that consistent pace of life is easier to detect across species than within species, as evolution has had more time to produce clear trade‐offs (Agrawal 2020). However, the more immediate constraint on intraspecific divergence may be gene flow, as ongoing genetic exchange between populations at different elevations can homogenise trait variation even under divergent selection (Lenormand 2002). Our results are consistent with this interpretation—we compared populations across a broad taxonomic sample encompassing 104 species and 10 families, yet found no consistent elevational patterns. Interspecific comparisons, which are generally not affected by gene flow and also benefit from the passage of time, may still reveal POLS signals that intraspecific tests cannot detect. Still other authors note that pace of life syndrome differences can be observed between individuals of the same species and between sexes (Dammhahn et al. 2018; Hämäläinen et al. 2018; Royauté et al. 2018; Arnqvist et al. 2022), suggesting that the relevant scale for detecting POLS may be narrower than environmental gradients. The formal development of POLS theory has lagged behind empirical applications (Mathot and Frankenhuis 2018; Laskowski et al. 2021), and our results underscore the need for more nuanced theoretical frameworks that account for phylogenetic constraints, ecological diversity, and context‐dependency in trait evolution.

## 5 Limitations

As part of our literature survey, we tried to assemble as broad a dataset as possible from various sources which were not always related to our own goal; therefore, some aspects may limit the generality of our findings. First, and the most obvious one, is the fact that we did not find enough data on some key traits such as growth rate, survival, metabolic rate, and our dataset does not include behavioural traits. Second, we were limited in our scope by the amount of data we could accrue, since we were looking for low‐high population pairs; as such we could only include 104 species belonging to 10 families, while as of January 2025 there are 11,991 named species of squamates in 76 families. Consequently, our results might not accurately capture patterns of trait divergence if our sample of studies overall or within taxonomic units is unrepresentative. Third, several geographic regions are also underrepresented in our study, and this includes most of the continents of South America, Africa and Oceania. Finally, our results were limited by selecting only studies published in English, which can lead to biases in overall results (Konno et al. 2020) as previous studies have shown that significant portions of literature related to biodiversity were not published in English (Amano et al. 2016).

## 6 Conclusions

Our meta‐analysis suggests that pace of life syndromes do not provide a useful framework for understanding intraspecific squamate responses to elevational gradients, although an elevational POLS signal may still be present in interspecific comparisons, which were beyond the scope of the current study. This negative result is both novel and important, contributing to growing evidence questioning the broad applicability of POLS theory while revealing that life‐history evolution is more complex and context‐dependent than syndrome‐based approaches suggest. Rather than representing a failure to detect existing patterns, our findings reveal fundamental limitations in applying universal syndrome concepts across diverse ecological and evolutionary contexts. Future research should focus on understanding the taxon‐specific and context‐dependent mechanisms that govern trait evolution, embracing the complexity and diversity of life history strategies rather than seeking universal syndromes.

## Author Contributions

**Tiberiu C. Sahlean:** conceptualization, data collection, formal analysis, methodology, validation, visualisation, writing – original draft, writing – review and editing, funding acquisition. **Ryan A. Martin:** conceptualization, formal analysis, methodology, validation, visualisation, writing – original draft, writing – review and editing.

## Funding

This work was supported by Academia Româna, RO1567‐IBB09/2026.

## Conflicts of Interest

The authors declare no conflicts of interest.

## Supporting information


**Data S1:** ele70343‐sup‐0001‐Supinfo.zip.

## Data Availability

Data, Supporting Information and code are archived on OSF at https://osf.io/bn3zd/?view_only=412b2bb442ec490393afe6defcca1faa.

## References

[ele70343-bib-0001] Adolph, S. C. , and W. P. Porter . 1993. “Temperature, Activity, and Lizard Life Histories.” American Naturalist 142: 273–295. 10.1086/285538 19425979

[ele70343-bib-0002] Agrawal, A. A. 2020. “A Scale‐Dependent Framework for Trade‐Offs, Syndromes, and Specialization in Organismal Biology.” Ecology 101: e02924. 31660584 10.1002/ecy.2924

[ele70343-bib-0003] Alice Boyle, W. , B. K. Sandercock , and K. Martin . 2016. “Patterns and Drivers of Intraspecific Variation in Avian Life History Along Elevational Gradients: A Meta‐Analysis.” Biological Reviews 91: 469–482. 25765584 10.1111/brv.12180

[ele70343-bib-0004] Amano, T. , J. P. González‐Varo , and W. J. Sutherland . 2016. “Languages Are Still a Major Barrier to Global Science.” PLoS Biology 14: e2000933. 28033326 10.1371/journal.pbio.2000933 PMC5199034

[ele70343-bib-0005] Angilletta, J. , P. H. Niewiarowski , A. E. Dunham , A. D. Leaché , and W. P. Porter . 2004. “Bergmann's Clines in Ectotherms: Illustrating a Life‐History Perspective With Sceloporine Lizards.” American Naturalist 164: E168. 10.1086/425222 29641924

[ele70343-bib-0006] Araya‐Ajoy, Y. G. , G. H. Bolstad , J. Brommer , V. Careau , N. J. Dingemanse , and J. Wright . 2018. “Demographic Measures of an Individual's “Pace of Life”: Fecundity Rate, Lifespan, Generation Time, or a Composite Variable?” Behavioral Ecology and Sociobiology 72: 75.

[ele70343-bib-0007] Arnqvist, G. , J. Rönn , C. Watson , J. Goenaga , and E. Immonen . 2022. “Concerted Evolution of Metabolic Rate, Economics of Mating, Ecology, and Pace of Life Across Seed Beetles.” Proceedings of the National Academy of Sciences 119: e2205564119. 10.1073/pnas.2205564119 PMC9388118 35943983

[ele70343-bib-0008] Ashton, K. G. 2004. “Sensitivity of Intraspecific Latitudinal Clines of Body Size for Tetrapods to Sampling, Latitude and Body Size.” Integrative and Comparative Biology 44: 403–412. 21676726 10.1093/icb/44.6.403

[ele70343-bib-0009] Ashton, K. G. , and C. R. Feldman . 2003. “Bergmann's Rule in Nonavian Reptiles: Turtles Follow It, Lizards and Snakes Reverse It.” Evolution 57: 1151–1163. 12836831 10.1111/j.0014-3820.2003.tb00324.x

[ele70343-bib-0010] Bachmann, J. C. , A. Jansen van Rensburg , M. Cortazar‐Chinarro , A. Laurila , and J. Van Buskirk . 2020. “Gene Flow Limits Adaptation Along Steep Environmental Gradients.” American Naturalist 195: E67–E86. 10.1086/707209 32097047

[ele70343-bib-0011] Balasubramaniam, P. , and J. T. Rotenberry . 2016. “Elevation and Latitude Interact to Drive Life‐History Variation in Precocial Birds: A Comparative Analysis Using Galliformes.” Journal of Animal Ecology 85: 1528–1539. 27392151 10.1111/1365-2656.12570

[ele70343-bib-0012] Bielby, J. , G. M. Mace , O. R. P. Bininda‐Emonds , et al. 2007. “The Fast‐Slow Continuum in Mammalian Life History: An Empirical Reevaluation.” American Naturalist 169: 748–757. 10.1086/516847 17479461

[ele70343-bib-0013] Binder, T. R. , A. D. M. Wilson , S. M. Wilson , C. D. Suski , J.‐G. J. Godin , and S. J. Cooke . 2016. “Is There a Pace‐Of‐Life Syndrome Linking Boldness and Metabolic Capacity for Locomotion in Bluegill Sunfish?” Animal Behaviour 121: 175–183.

[ele70343-bib-0014] Blouin‐Demers, G. , and P. Nadeau . 2005. “The Cost‐Benefit Model of Thermoregulation Does Not Predict Lizard Thermoregulatory Behavior.” Ecology 86: 560–566.

[ele70343-bib-0015] Bronikowski, A. , and D. Vleck . 2010. “Metabolism, Body Size and Life Span: A Case Study in Evolutionarily Divergent Populations of the Garter Snake ( *Thamnophis elegans* ).” Integrative and Comparative Biology 50: 880–887. 21558247 10.1093/icb/icq132

[ele70343-bib-0016] Bronikowski, A. M. 2000. “Experimental Evidence for the Adaptive Evolution of Growth Rate in the Garter Snake *Thamnophis Elegans* .” Evolution 54: 1760–1767. 11108602 10.1111/j.0014-3820.2000.tb00719.x

[ele70343-bib-0017] Bronikowski, A. M. , and S. J. Arnold . 1999. “The Evolutionary Ecology of Life History Variation in the Garter Snake *Thamnophis Elegans* .” Ecology 80: 2314–2325. 10.1890/08-0850.1 19341142

[ele70343-bib-0018] Buckley, L. B. 2010. “The Range Implications of Lizard Traits in Changing Environments.” Global Ecology and Biogeography 19: 452–464.

[ele70343-bib-0019] Camfield, A. F. , S. F. Pearson , and K. Martin . 2010. “Life History Variation Between High and Low Elevation Subspecies of Horned Larks *Eremophila* spp.” Journal of Avian Biology 41: 273–281.

[ele70343-bib-0020] Dammhahn, M. , N. J. Dingemanse , P. T. Niemelä , and D. Réale . 2018. “Pace‐Of‐Life Syndromes: A Framework for the Adaptive Integration of Behaviour, Physiology and Life History.” Behavioral Ecology and Sociobiology 72: 62.

[ele70343-bib-0021] Diamond, S. E. , and R. A. Martin . 2021. “Buying Time: Plasticity and Population Persistence.” In Phenotypic Plasticity & Evolution: Causes, Consequences, Controversies, edited by D. W. Pfenning , 185–209. CRC Press.

[ele70343-bib-0022] Díaz, J. A. , and S. Cabezas‐Díaz . 2004. “Seasonal Variation in the Contribution of Different Behavioural Mechanisms to Lizard Thermoregulation.” Functional Ecology 18: 867–875.

[ele70343-bib-0023] Dubiner, S. , R. Aguilar , R. O. Anderson , et al. 2024. “A Global Analysis of Field Body Temperatures of Active Squamates in Relation to Climate and Behaviour.” Global Ecology and Biogeography 33: e13808.

[ele70343-bib-0024] Friedrich, J. O. , N. K. J. Adhikari , and J. Beyene . 2008. “The Ratio of Means Method as an Alternative to Mean Differences for Analyzing Continuous Outcome Variables in Meta‐Analysis: A Simulation Study.” BMC Medical Research Methodology 8: 32. 18492289 10.1186/1471-2288-8-32 PMC2430201

[ele70343-bib-0025] Fu, R. , G. Gartlehner , M. Grant , et al. 2011. “Conducting Quantitative Synthesis When Comparing Medical Interventions: AHRQ and the Effective Health Care Program.” Journal of Clinical Epidemiology 64: 1187–1197. 21477993 10.1016/j.jclinepi.2010.08.010

[ele70343-bib-0026] Gangloff, E. J. , M. Chow , V. Leos‐Barajas , S. Hynes , B. Hobbs , and A. M. Sparkman . 2017. “Integrating Behaviour Into the Pace‐Of‐Life Continuum: Divergent Levels of Activity and Information Gathering in Fast‐ and Slow‐Living Snakes.” Behavioural Processes 142: 156–163. 28648696 10.1016/j.beproc.2017.06.006

[ele70343-bib-0027] Garcia‐Porta, J. , I. Irisarri , M. Kirchner , et al. 2019. “Environmental Temperatures Shape Thermal Physiology as Well as Diversification and Genome‐Wide Substitution Rates in Lizards.” Nature Communications 10: 4077. 10.1038/s41467-019-11943-x PMC6733905 31501432

[ele70343-bib-0028] Ghalambor, C. K. , R. B. Huey , P. R. Martin , J. J. Tewksbury , and G. Wang . 2006. “Are Mountain Passes Higher in the Tropics? Janzen's Hypothesis Revisited.” Integrative and Comparative Biology 46: 5–17. 21672718 10.1093/icb/icj003

[ele70343-bib-0029] Gopal, A. C. , K. Alujević , and M. L. Logan . 2023. “Temperature and the Pace of Life.” Behavioral Ecology and Sociobiology 77: 59.

[ele70343-bib-0030] Goulet, C. T. , M. B. Thompson , and D. G. Chapple . 2017. “Repeatability and Correlation of Physiological Traits: Do Ectotherms Have a “Thermal Type”?” Ecology and Evolution 7: 710–719. 28116065 10.1002/ece3.2632 PMC5243194

[ele70343-bib-0031] Hämäläinen, A. , E. Immonen , M. Tarka , and W. Schuett . 2018. “Evolution of Sex‐Specific Pace‐Of‐Life Syndromes: Causes and Consequences.” Behavioral Ecology and Sociobiology 72: 50. 10.1007/s00265-018-2462-1 PMC5856903 29576676

[ele70343-bib-0032] Hämäläinen, A. M. , A. Guenther , S. C. Patrick , and W. Schuett . 2021. “Environmental Effects on the Covariation Among Pace‐Of‐Life Traits.” Ethology 127: 32–44.

[ele70343-bib-0033] Healy, K. , T. H. G. Ezard , O. R. Jones , R. Salguero‐Gómez , and Y. M. Buckley . 2019. “Animal Life History Is Shaped by the Pace of Life and the Distribution of Age‐Specific Mortality and Reproduction.” Nature Ecology & Evolution 3: 1217–1224. 31285573 10.1038/s41559-019-0938-7

[ele70343-bib-0034] Hedges, L. V. , J. Gurevitch , and P. S. Curtis . 1999. “The Meta‐Analysis of Response Ratios in Experimental Ecology.” Ecology 80: 1150–1156.

[ele70343-bib-0035] Hertz, P. E. , R. B. Huey , and R. D. Stevenson . 1993. “Evaluating Temperature Regulation by Field‐Active Ectotherms: The Fallacy of the Inappropriate Question.” American Naturalist 142: 796–818. 10.1086/285573 19425957

[ele70343-bib-0036] Hille, S. M. , and C. B. Cooper . 2015. “Elevational Trends in Life Histories: Revising the Pace‐Of‐Life Framework.” Biological Reviews 90: 204–213. 24673806 10.1111/brv.12106

[ele70343-bib-0037] Hodkinson, I. D. 2005. “Terrestrial Insects Along Elevation Gradients: Species and Community Responses to Altitude.” Biological Reviews 80: 489–513. 16094810 10.1017/s1464793105006767

[ele70343-bib-0040] Huey, R. B. , P. E. Hertz , and B. Sinervo . 2003. “Behavioral Drive Versus Behavioral Inertia in Evolution: A Null Model Approach.” American Naturalist 161: 357–366. 10.1086/346135 12699218

[ele70343-bib-0038] Huey, R. B. , and J. G. Kingsolver . 1993. “Evolution of Resistance to High Temperature in Ectotherms.” American Naturalist 142: S21–S46.

[ele70343-bib-0039] Huey, R. B. , and M. Slatkin . 1976. “Cost and Benefits of Lizard Thermoregulation.” QUARTERLY REVIEW OF BIOLOGY 51: 363–384. 981504 10.1086/409470

[ele70343-bib-0041] Janzen, D. H. 1967. “Why Mountain Passes Are Higher in the Tropics.” American Naturalist 101: 233–249.

[ele70343-bib-0042] Keller, I. , J. M. Alexander , R. Holderegger , and P. J. Edwards . 2013. “Widespread Phenotypic and Genetic Divergence Along Altitudinal Gradients in Animals.” Journal of Evolutionary Biology 26: 2527–2543. 24128377 10.1111/jeb.12255

[ele70343-bib-0043] Konno, K. , M. Akasaka , C. Koshida , et al. 2020. “Ignoring Non‐English‐Language Studies May Bias Ecological Meta‐Analyses.” Ecology and Evolution 10: 6373–6384. 32724519 10.1002/ece3.6368 PMC7381574

[ele70343-bib-0044] Körner, C. 2007. “The Use of ‘Altitude’ in Ecological Research.” Trends in Ecology & Evolution 22: 569–574. 17988759 10.1016/j.tree.2007.09.006

[ele70343-bib-0045] Labra, A. , J. Pienaar , and T. F. Hansen . 2009. “Evolution of Thermal Physiology in *Liolaemus* Lizards: Adaptation, Phylogenetic Inertia, and Niche Tracking.” American Naturalist 174: 204–220. 10.1086/600088 19538089

[ele70343-bib-0046] Laiolo, P. , and J. R. Obeso . 2017. “High Mountain Conservation in a Changing World.” In Life‐History Responses to the Altitudinal Gradient, 253–283. Springer International Publishing.

[ele70343-bib-0047] Lajeunesse, M. J. 2015. “Bias and Correction for the Log Response Ratio in Ecological Meta‐Analysis.” Ecology 96: 2056–2063. 26405731 10.1890/14-2402.1

[ele70343-bib-0048] Lange, Z. K. , B. L. Bodensteiner , D. J. Nicholson , et al. 2025. “Lizard Thermal Physiology Drives Abundance Peaks Along Climate Gradients but Only Weakly Predicts Distributional Limits.” American Naturalist 206: E47–E62. 10.1086/736566 40816254

[ele70343-bib-0049] Laskowski, K. L. , M. Moiron , and P. T. Niemelä . 2021. “Integrating Behavior in Life‐History Theory: Allocation Versus Acquisition?” Trends in Ecology & Evolution 36: 132–138. 33203522 10.1016/j.tree.2020.10.017

[ele70343-bib-0050] Lenormand, T. 2002. “Gene Flow and the Limits to Natural Selection.” Trends in Ecology & Evolution 17: 183–189.

[ele70343-bib-0051] Lourdais, O. , M. Guillon , D. DeNardo , and G. Blouin‐Demers . 2013. “Cold Climate Specialization: Adaptive Covariation Between Metabolic Rate and Thermoregulation in Pregnant Vipers.” Physiology & Behavior 119: 149–155. 23769691 10.1016/j.physbeh.2013.05.041

[ele70343-bib-0052] Lovell, R. S. L. , S. Collins , S. H. Martin , A. L. Pigot , and A. B. Phillimore . 2023. “Space‐For‐Time Substitutions in Climate Change Ecology and Evolution.” Biological Reviews 98: 2243–2270. 37558208 10.1111/brv.13004

[ele70343-bib-0053] Lymburner, A. H. , and G. Blouin‐Demers . 2020. “Changes in Thermal Quality of the Environment Along an Elevational Gradient Affect Investment in Thermoregulation by Yarrow's Spiny Lizards.” Journal of Zoology 312: 133–143.

[ele70343-bib-0054] Martin, L. B., II , D. Hasselquist , and M. Wikelski . 2006. “Investment in Immune Defense Is Linked to Pace of Life in House Sparrows.” Oecologia 147: 565–575. 16450181 10.1007/s00442-005-0314-y

[ele70343-bib-0055] Martin, R. A. , C. R. B. da Silva , M. P. Moore , and S. E. Diamond . 2023. “When Will a Changing Climate Outpace Adaptive Evolution?” WIREs Climate Change 14: e852.

[ele70343-bib-0056] Martin, T. E. 1996. “Life History Evolution in Tropical and South Temperate Birds: What Do we Really Know?” Journal of Avian Biology 27: 263–272.

[ele70343-bib-0057] Mathies, T. , and R. M. Andrews . 1995. “Thermal and Reproductive Biology of High and Low Elevation Populations of the Lizard *Sceloporus scalaris*: Implications for the Evolution of Viviparity.” Oecologia 104: 101–111. 28306919 10.1007/BF00365568

[ele70343-bib-0058] Mathot, K. J. , N. J. Dingemanse , and S. Nakagawa . 2019. “The Covariance Between Metabolic Rate and Behaviour Varies Across Behaviours and Thermal Types: Meta‐Analytic Insights.” Biological Reviews 94: 1056–1074. 30588731 10.1111/brv.12491

[ele70343-bib-0059] Mathot, K. J. , and W. E. Frankenhuis . 2018. “Models of Pace‐Of‐Life Syndromes (POLS): A Systematic Review.” Behavioral Ecology and Sociobiology 72: 41.

[ele70343-bib-0060] McCain, C. M. 2010. “Global Analysis of Reptile Elevational Diversity.” Global Ecology and Biogeography 19: 541–553.

[ele70343-bib-0061] Mesquita, D. O. , G. C. Costa , G. R. Colli , et al. 2016. “Life‐History Patterns of Lizards of the World.” American Naturalist 187: 689–705. 10.1086/686055 27172590

[ele70343-bib-0062] Michelangeli, M. , C. T. Goulet , H. S. Kang , B. B. M. Wong , and D. G. Chapple . 2018. “Integrating Thermal Physiology Within a Syndrome: Locomotion, Personality and Habitat Selection in an Ectotherm.” Functional Ecology 32: 970–981.

[ele70343-bib-0063] Morin, X. , and I. Chuine . 2006. “Niche Breadth, Competitive Strength and Range Size of Tree Species: A Trade‐Off Based Framework to Understand Species Distribution.” Ecology Letters 9: 185–195. 16958884 10.1111/j.1461-0248.2005.00864.x

[ele70343-bib-0064] Muñoz, M. M. 2022. “The Bogert Effect, a Factor in Evolution.” Evolution 76: 49–66. 34676550 10.1111/evo.14388

[ele70343-bib-0065] Muñoz, M. M. , and B. L. Bodensteiner . 2019. “Janzen's Hypothesis Meets the Bogert Effect: Connecting Climate Variation, Thermoregulatory Behavior, and Rates of Physiological Evolution.” Integrative Organismal Biology 1: 1–12. 10.1093/iob/oby002 PMC7671085 33791511

[ele70343-bib-0066] Muñoz, M. M. , and J. B. Losos . 2018. “Thermoregulatory Behavior Simultaneously Promotes and Forestalls Evolution in a Tropical Lizard.” American Naturalist 191: E15–E26. 10.1086/694779 29244559

[ele70343-bib-0067] Nakagawa, S. , M. Lagisz , M. D. Jennions , et al. 2022. “Methods for Testing Publication Bias in Ecological and Evolutionary Meta‐Analyses.” Methods in Ecology and Evolution 13: 4–21.

[ele70343-bib-0068] Nakagawa, S. , M. Lagisz , R. E. O'Dea , et al. 2023. “orchaRd 2.0: An R Package for Visualising Meta‐Analyses With Orchard Plots.” Methods in Ecology and Evolution 14: 2003–2010.

[ele70343-bib-0069] Nakagawa, S. , R. Poulin , K. Mengersen , et al. 2015. “Meta‐Analysis of Variation: Ecological and Evolutionary Applications and Beyond.” Methods in Ecology and Evolution 6: 143–152.

[ele70343-bib-0070] Nakagawa, S. , Y. Yang , E. L. Macartney , R. Spake , and M. Lagisz . 2023. “Quantitative Evidence Synthesis: A Practical Guide on Meta‐Analysis, Meta‐Regression, and Publication Bias Tests for Environmental Sciences.” Environmental Evidence 12: 8. 39294795 10.1186/s13750-023-00301-6 PMC11378872

[ele70343-bib-0071] Noble, D. W. A. , M. Lagisz , R. E. O'dea , and S. Nakagawa . 2017. “Nonindependence and Sensitivity Analyses in Ecological and Evolutionary Meta‐Analyses.” Molecular Ecology 26: 2410–2425. 28133832 10.1111/mec.14031

[ele70343-bib-0072] Olalla‐Tárraga, M. Á. , M. Á. Rodríguez , and B. A. Hawkins . 2006. “Broad‐Scale Patterns of Body Size in Squamate Reptiles of Europe and North America.” Journal of Biogeography 33: 781–793.

[ele70343-bib-0073] Oli, M. K. 2004. “The Fast–Slow Continuum and Mammalian Life‐History Patterns: An Empirical Evaluation.” Basic and Applied Ecology 5: 449–463.

[ele70343-bib-0074] Packard, G. C. , C. R. Tracy , and J. J. Roth . 1977. “The Physiological Ecology of Reptilian Eggs and Embryos, and the Evolution of Viviparity Within the Class Reptilia.” Biological Reviews 52: 71–105. 319843 10.1111/j.1469-185x.1977.tb01346.x

[ele70343-bib-0075] Palacios, M. G. , J. E. Cunnick , and A. M. Bronikowski . 2013. “Complex Interplay of Body Condition, Life History, and Prevailing Environment Shapes Immune Defenses of Garter Snakes in the Wild.” Physiological and Biochemical Zoology 86: 547–558. 23995485 10.1086/672371

[ele70343-bib-0076] Pincheira‐Donoso, D. , and S. Meiri . 2013. “An Intercontinental Analysis of Climate‐Driven Body Size Clines in Reptiles: No Support for Patterns, no Signals of Processes.” Evolutionary Biology 40: 562–578.

[ele70343-bib-0077] Plasman, M. , A. Gonzalez‐Voyer , A. Bautista , and A. H. Díaz De La Vega‐Pérez . 2025. “Flexibility in Thermal Requirements: A Comparative Analysis of the Wide‐Spread Lizard Genus *Sceloporus* .” Integrative Zoology 20: 850–866. 38880782 10.1111/1749-4877.12860

[ele70343-bib-0078] Polverino, G. , F. Santostefano , C. Díaz‐Gil , and T. Mehner . 2018. “Ecological Conditions Drive Pace‐Of‐Life Syndromes by Shaping Relationships Between Life History, Physiology and Behaviour in Two Populations of Eastern Mosquitofish.” Scientific Reports 8: 14673. 30279465 10.1038/s41598-018-33047-0 PMC6168454

[ele70343-bib-0079] Promislow, D. E. L. , and P. H. Harvey . 1990. “Living Fast and Dying Young: A Comparative Analysis of Life‐History Variation Among Mammals.” Journal of Zoology 220: 417–437.

[ele70343-bib-0080] Pustejovsky, J. E. 2018. “Using Response Ratios for Meta‐Analyzing Single‐Case Designs With Behavioral Outcomes.” Journal of School Psychology 68: 99–112. 29861034 10.1016/j.jsp.2018.02.003

[ele70343-bib-0081] R Core Team . 2022. “A Language and Environment for Statistical Computing.” R Foundation for Statistical Computing, Vienna, Austria. https://www.R‐project.org/.

[ele70343-bib-0082] Réale, D. , D. Garant , M. M. Humphries , P. Bergeron , V. Careau , and P.‐O. Montiglio . 2010. “Personality and the Emergence of the Pace‐Of‐Life Syndrome Concept at the Population Level.” Philosophical Transactions of the Royal Society, B: Biological Sciences 365: 4051–4063. 10.1098/rstb.2010.0208 PMC2992747 21078657

[ele70343-bib-0083] Refsnider, J. M. , S. S. Qian , H. M. Streby , et al. 2018. “Reciprocally Transplanted Lizards Along an Elevational Gradient Match Light Environment Use of Local Lizards via Phenotypic Plasticity.” Functional Ecology 32: 1227–1236.

[ele70343-bib-0084] Ricklefs, R. E. 2000. “Lack, Skutch, and Moreau: The Early Development of Life‐History Thinking.” Condor 102: 3–8.

[ele70343-bib-0085] Ricklefs, R. E. , and M. Wikelski . 2002. “The Physiology/Life‐History Nexus.” Trends in Ecology & Evolution 17: 462–468.

[ele70343-bib-0086] Rivera‐Rea, J. , L. Macotela , G. Moreno‐Rueda , et al. 2023. “Thermoregulatory Behavior Varies With Altitude and Season in the Sceloporine Mesquite Lizard.” Journal of Thermal Biology 114: 103539. 37344013 10.1016/j.jtherbio.2023.103539

[ele70343-bib-0087] Rodríguez‐Díaz, T. , and F. Braña . 2012. “Altitudinal Variation in Egg Retention and Rates of Embryonic Development in Oviparous *Zootoca vivipara* Fits Predictions From the Cold‐Climate Model on the Evolution of Viviparity.” Journal of Evolutionary Biology 25: 1877–1887. 22862292 10.1111/j.1420-9101.2012.02575.x

[ele70343-bib-0088] Rohr, D. H. 1997. “Demographic and Life‐History Variation in Two Proximate Populations of a Viviparous Skink Separated by a Steep Altitudinal Gradient.” Journal of Animal Ecology 66: 567–578.

[ele70343-bib-0089] Royauté, R. , M. A. Berdal , C. R. Garrison , and N. A. Dochtermann . 2018. “Paceless Life? A Meta‐Analysis of the Pace‐of‐Life Syndrome Hypothesis.” Behavioral Ecology and Sociobiology 72: 64.

[ele70343-bib-0090] Santostefano, F. , A. J. Wilson , P. T. Niemelä , and N. J. Dingemanse . 2017. “Behavioural Mediators of Genetic Life‐History Trade‐Offs: A Test of the Pace‐Of‐Life Syndrome Hypothesis in Field Crickets.” Proceedings of the Royal Society B: Biological Sciences 284: 20171567. 10.1098/rspb.2017.1567 PMC5647303 28978731

[ele70343-bib-0091] Scharf, I. , A. Feldman , M. Novosolov , et al. 2015. “Late Bloomers and Baby Boomers: Ecological Drivers of Longevity in Squamates and the Tuatara.” Global Ecology and Biogeography 24: 396–405.

[ele70343-bib-0092] Sears, M. W. , and M. J. Angilletta Jr. 2003. “Life‐History Variation in the Sagebrush Lizard: Phenotypic Plasticity or Local Adaptation?” Ecology 84: 1624–1634.

[ele70343-bib-0093] Sears, M. W. , and M. J. Angilletta Jr. 2004. “Body Size Clines in *Sceloporus* Lizards: Proximate Mechanisms and Demographic Constraints.” Integrative and Comparative Biology 44: 433–442. 21676729 10.1093/icb/44.6.433

[ele70343-bib-0094] Seigel, R. A. , M. M. Huggins , and N. B. Ford . 1987. “Reduction in Locomotor Ability as a Cost of Reproduction in Gravid Snakes.” Oecologia 73: 481–485. 28311962 10.1007/BF00379404

[ele70343-bib-0095] Senior, A. M. , W. Viechtbauer , and S. Nakagawa . 2020. “Revisiting and Expanding the Meta‐Analysis of Variation: The Log Coefficient of Variation Ratio.” Research Synthesis Methods 11: 553–567. 32431099 10.1002/jrsm.1423

[ele70343-bib-0096] Shine, R. 1980. ““Costs” of Reproduction in Reptiles.” Oecologia 46: 92–100. 28310632 10.1007/BF00346972

[ele70343-bib-0097] Shine, R. 1985. “The Evolution of Viviparity in Reptiles: An Ecological Analysis.” In Biology of the Reptilia Vol. 15, 607–681. John Wiley & Sons.

[ele70343-bib-0098] Shine, R. 2005. “Life‐History Evolution in Reptiles.” Annual Review of Ecology, Evolution, and Systematics 36: 23–46.

[ele70343-bib-0099] Shine, R. , and P. Harlow . 1993. “Maternal Thermoregulation Influences Offspring Viability in a Viviparous Lizard.” Oecologia 96: 122–127. 28313762 10.1007/BF00318039

[ele70343-bib-0100] Shine, R. , and P. S. Harlow . 1996. “Maternal Manipulation of Offspring Phenotypes via Nest‐Site Selection in an Oviparous Lizard.” Ecology 77: 1808–1817.

[ele70343-bib-0101] Sinervo, B. 1990a. “The Evolution of Maternal Investment in Lizards: An Experimental and Comparative Analysis of Egg Size and Its Effects on Offspring Performance.” Evolution 44: 279–294. 28564384 10.1111/j.1558-5646.1990.tb05198.x

[ele70343-bib-0102] Sinervo, B. 1990b. “Evolution of Thermal Physiology and Growth Rate Between Populations of the Western Fence Lizard ( *Sceloporus occidentalis* ).” Oecologia 83: 228–237. 22160116 10.1007/BF00317757

[ele70343-bib-0103] Sinervo, B. , R. Hedges , and S. C. Adolph . 1991. “Decreased Sprint Speed as A Cost of Reproduction in the Lizard *Sceloporus occidentalis*: Variation Among Populations.” Journal of Experimental Biology 155: 323–336.

[ele70343-bib-0104] Stearns, S. C. 1977. “The Evolution of Life History Traits: A Critique of the Theory and a Review of the Data.” Annual Review of Ecology, Evolution, and Systematics 8: 145–171.

[ele70343-bib-0105] Stearns, S. C. 1992. The Evolution of Life Histories. Oxford University Press.

[ele70343-bib-0106] Tarka, M. , A. Guenther , P. T. Niemelä , S. Nakagawa , and D. W. A. Noble . 2018. “Sex Differences in Life History, Behavior, and Physiology Along a Slow‐Fast Continuum: A Meta‐Analysis.” Behavioral Ecology and Sociobiology 72: 132. 30100667 10.1007/s00265-018-2534-2 PMC6060830

[ele70343-bib-0107] Telemeco, R. S. , M. J. Elphick , and R. Shine . 2009. “Nesting Lizards (*Bassiana duperreyi*) Compensate Partly, but Not Completely, for Climate Change.” Ecology 90: 17–22. 19294908 10.1890/08-1452.1

[ele70343-bib-0108] Tinkle, D. W. , and J. W. Gibbons . 1977. “The Distribution and Evolution of Viviparity in Reptiles.” Miscellaneous Publications‐Museum of Zoology, University of Michigan 154: 1–47.

[ele70343-bib-0109] Turko, A. J. , J. E. Doherty , I. Yin‐Liao , et al. 2019. “Prolonged Survival Out of Water Is Linked to a Slow Pace of Life in a Self‐Fertilizing Amphibious Fish.” Journal of Experimental Biology 222: jeb209270. 31796606 10.1242/jeb.209270

[ele70343-bib-0110] Tüzün, N. , and R. Stoks . 2018. “Evolution of Geographic Variation in Thermal Performance Curves in the Face of Climate Change and Implications for Biotic Interactions.” Current Opinion in Insect Science 29: 78–84. 30551830 10.1016/j.cois.2018.07.004

[ele70343-bib-0111] Uetz, P. , P. Freed , R. Aguilar , F. Reyes , J. Kudera , and J. Hošek . 2024. “The Reptile Database.” http://www.reptile‐database.org.

[ele70343-bib-0112] van Noordwijk, A. J. , and G. de Jong . 1986. “Acquisition and Allocation of Resources: Their Influence on Variation in Life History Tactics.” American Naturalist 128: 137–142.

[ele70343-bib-0113] Verheyen, J. , N. Tüzün , and R. Stoks . 2019. “Using Natural Laboratories to Study Evolution to Global Warming: Contrasting Altitudinal, Latitudinal, and Urbanization Gradients.” Current Opinion in Insect Science 35: 10–19. 31301449 10.1016/j.cois.2019.06.001

[ele70343-bib-0114] Viechtbauer, W. 2010. “Conducting Meta‐Analyses in R With the Metafor Package.” Journal of Statistical Software 36: 1–48.

[ele70343-bib-0115] Vitt, L. J. , and J. P. Caldwell . 2014. Herpetology. An Introductory Biology of Amphibians and Reptiles. Fourth ed. Academic Press.

[ele70343-bib-0116] Vleck, D. 1991. “Water Economy and Solute Regulation of Reptilian and Avian Embryos.” In Egg Incubation: Its Effects on Embryonic Development in Birds and Reptiles, edited by M. W. J. Ferguson , 245–259. Cambridge University Press.

[ele70343-bib-0117] Wang, I. J. , and G. S. Bradburd . 2014. “Isolation by Environment.” Molecular Ecology 23: 5649–5662. 25256562 10.1111/mec.12938

[ele70343-bib-0118] Weatherhead, P. J. , J. H. Sperry , G. L. F. Carfagno , and G. Blouin‐Demers . 2012. “Latitudinal Variation in Thermal Ecology of North American Ratsnakes and Its Implications for the Effect of Climate Warming on Snakes.” Journal of Thermal Biology 37: 273–281.

[ele70343-bib-0119] Wiersma, P. , A. Muñoz‐Garcia , A. Walker , and J. B. Williams . 2007. “Tropical Birds Have a Slow Pace of Life.” Proceedings of the National Academy of Sciences 104: 9340–9345. 10.1073/pnas.0702212104 PMC1890496 17517640

[ele70343-bib-0120] Wikelski, M. , L. Spinney , W. Schelsky , A. Scheuerlein , and E. Gwinner . 2003. “Slow Pace of Life in Tropical Sedentary Birds: A Common‐Garden Experiment on Four Stonechat Populations From Different Latitudes.” Proceedings of the Royal Society of London. Series B: Biological Sciences 270: 2383–2388. 10.1098/rspb.2003.2500 PMC1691521 14667355

