# Supplementary material for: No Evidence for Pace of Life Evolution Along Elevational Gradients in Squamate Reptiles
Source: Ecol Lett. 2026 Feb 23;29(2):e70343. doi: 10.1111/ele.70343 (PMC12928677; doi:10.1111/ele.70343)
Supplement: Supplementary file 1 — Data S1: ele70343‐sup‐0001‐Supinfo.zip. [file ELE-29-0-s001.zip › Appendix S3.docx]

**No evidence for pace of life evolution along elevational gradients in squamate reptiles**

Tiberiu C. SAHLEAN^1, *^, Ryan A. MARTIN^2^

^1^ Institute of Biology Bucharest, Romanian Academy, Bucharest, Romania

^2^ Department of Biology, Case Western Reserve University, Cleveland, Ohio, USA

^*^ Corresponding author: Tiberiu C. SAHLEAN, [tiberiu.sahlean@ibiol.ro](mailto:tiberiu.sahlean@ibiol.ro)

**Supplementary methods**

*Data collection*

We conducted an extensive literature search up to the end of 2024 for empirical field studies that reported trait data (life-history or physiology) for two or more populations of the same species located at different elevations. We initially conducted our search on Google Scholar, since results from the Web of Science are also indexed here, using multiple combinations of search terms (Table S1). Since the relevance of the search is diluted with every page, we only examined records until the results were deemed no longer relevant, and then we moved on to the next combination of keywords. As Scholar displays items which have already been accessed in a distinct color, we used this feature to avoid duplicate records, and the number of articles saved decreased significantly in subsequent inquiries. When new records were deemed relevant, we quickly skimmed the manuscript to check if (*i*) at least two populations located at different elevations were part of the study, (*ii*) summary data, charts or supplementary material were available; when these preconditions were met, we downloaded the reference and the article for a more thorough study later on. As a part of our data collection protocol, we only looked at peer-reviewed articles published in English. Subsequently we also replicated the search on Scopus using the same combinations of keywords (Table S1). The searches resulted in a total of 1916 records which we downloaded to our reference manager. The next step was to discard articles not relevant to our analysis and duplicates, which left us with 369 articles. Afterwards, we also looked at references in the downloaded articles for other records which we might have missed during our database search. This netted us another 24 articles for examination.

Finally, we excluded articles which did not offer summary statistics, usable figures for data extraction, supplementary material or which could not be geographically located in order to obtain elevation or latitudinal data. Articles where summary statistics were obtained from controlled laboratory conditions (e.g. measurements from neonates hatched in captivity) were also discarded, except cases when conditions were purposefully created to mimic those from the species’ natural environment. We created a database where we documented our examination of the remaining references (Appendix S1) and, in the end, we managed to extract data from 224 articles, of which 192 were used in our analyses (after excluding studies without standard deviation, standard errors or sample sizes).

Since our goal was to have as many traits as possible, we extracted all matching data from the articles first, with the aim of organizing them into categories afterwards. When summary statistics from multiple populations were presented in an article, we extracted data from the lowest and highest elevation populations, except cases where two or more populations from the same elevation were very close to each other (<1 km distance between them) in which case data were pooled. In such cases, or in cases where summary statistics were presented separately for various reasons (e.g. broken-down by sex, life stage or season) we used the following formula to obtain a combined mean (Higgins *et al.* 2024): $\mu_{combined}= \frac{((N_{1}*\mu_{1)}+\left( N_{2}*\mu_{2} \right)+\ldots+\left( N_{x}*\mu_{x} \right))}{(N_{1}+N_{2}+\ldots+N_{x})}$ , where *µ_1_*, *µ_2_*_,_ *µ_x_* are the means of the variables and *N_1_*, *N_2_* and *N_x_* are the corresponding sample sizes. Similarly, the pooled standard deviations were calculated using the formula ${SD}_{pooled}= \sqrt{\frac{\left( N_{1}-1 \right)s_{1}^{2}+\left( N_{2}-1 \right)s_{2}^{2}+\ldots+\left( N_{x}-1 \right)s_{x}^{2}}{(N_{1}+N_{2}+\ldots+N_{x}-x)}}$, where *N_1_*, *N_2_* and *N_x_* are the sample sizes of the groups, *s_1_*, *s_2_* through *s_x_* are the corresponding sample sizes and *x* is the number of sample sizes (Higgins *et al.* 2024). When errors were reported in the form of standard errors, we used the following formula to obtain standard deviations: $SD=SE\times\sqrt{N}$, where *SE* is the standard error and *N* is the sample size. In rare cases results were available in the form of median and range, so we used the formulas proposed by (Hozo *et al.* 2005) to obtain means and variances (which we then transformed to standard deviations). The formula for the mean is $\mu= \frac{a+2m+b}{4}$, where *a* and *b* are the low and the high end of the range, while *m* is the median and the formula for obtaining the variance is $s^{2}=\frac{1}{12}\left( \frac{{(a-2m+b)}^{2}}{4}+{(b-a)}^{2} \right)$. When confidence intervals were presented instead of standard deviations, we used the formula proposed by Higgins *et al.* (2024): $SD= \sqrt{N}\times(upper limit-lower limit)/3.92$, and replacing the value 3.92 depending on the corresponding confidence interval and the sample size, using the formula “=tinv(1-0.95, *k*-1) in Microsoft Excel, where *k* is the sample size.

When summary data was not presented in the form of tables or text, but usable figures, we used WebPlotDigitizer (<https://automeris.io/WebPlotDigitizer/>) to extract the data. In some cases, we contacted the authors of the primary papers directly to ask for the summary statistics.

*Meta-analytical methods*

We used the *t*-test instead of the default *z-*test for model coefficients in the *rma.mv* function (metafor package) and selected *dfs = ‘contain*’ to improve the approximation of degrees of freedom, as recommended by Viechtbauer (2010). When conducting meta-analyses, we selected trait categories where there were at least two studies for each family (Valentine *et al.* 2010) and at least ten studies overall. Meta-regressions were produced when there were at least four studies for each family and a minimum of twenty total effect sizes (Fu *et al.* 2011). This applied to meta-analyses and meta-regressions of response ratios as well as for the analysis of the coefficient of variation. Meta-regressions were performed to explore the effect of moderators when heterogeneity was high even if the difference between low and high elevation populations was not significant.

Our resulting database was composed of a heterogenous mixture of summary statistics, with multiple effect sizes extracted from a single study and the same populations based on shared measurements, and a multitude of species spanning the entire squamate clade, all of which can lead to biased estimates if they are not properly controlled (Noble *et al.* 2017; Nakagawa *et al.* 2022; Nakagawa *et al.* 2023b). As a result, we opted for a multi-level meta-analysis, where we tested for including effect size identity, study identity, location identity and species identity as random factors. Random factors were included in the meta-analytical models based on their ability to explain the total amount of heterogeneity (*I^2^*), and we excluded those with an overall contribution of less than 1%.

To account for phylogenetic relatedness we first downloaded a phylogenetic subset from VertLife (https://vertlife.org) webpage, which hosts the results of the study published by Tonini *et al.* (2016). We downloaded one thousand phylogenetic trees corresponding to species in our analyses and transformed them into an ultrametric dichotomous phylogeny in R assuming a , where the tips are equidistant from the root (Page & Holmes 1998). First we produced a consensus tree using the package *phytools*, then we transformed the tree to ultrametric and dichotomous using the *chronos* and *multi2di* functions in the *ape* package (Paradis & Schliep 2018). The branch lengths were used to create a phylogenetic correlation matrix (Cinar *et al.* 2022) following standard Brownian motion assumptions, scaled to have a maximum value of one. Subsequently, we introduced the correlation matrix in the multi-level meta-analysis to account for phylogenetic relatedness among species.

The impact of shared measurements was tested by constructing a variance-covariance matrix and conducting a sensitivity analysis with varying degrees of within-study sampling variance correlation (ρ) and examining the impact on the overall effect size (β_0_), as suggested by Nakagawa *et al.* (2023b). We evaluated multiple values of ρ (0.3, 0.5, 0.7, 0.9) but the results showed none or only minor overall effects (see Appendix S3, Tables S6-S7) so we continued the analyses with the original sampling variances instead of the variance-covariance matrix.

We estimated the degree of variance using the *I^2^* statistic, defined as the percentage of variability in the effect sizes that is not only the result of sampling error, which we then interpreted based on the threshold proposed by Higgins *et al.* (2003), where 25% is considered small, 50% is considered moderate and above 75% is considered a high degree of variance. Since we relied exclusively on multi-level meta-analyses and multi-level meta-regressions, we used the multi-level *I^2^* statistic from the *orchard 2.0* package (Nakagawa *et al.* 2023a), where the total amount of heterogeneity (*I^2^_total_*) is based on several components, such as differences between studies (*I^2^_study_*), differences within studies (*I^2^_effect_*), or the degree of phylogenetic relatedness (*I^2^_phylo_*).

*Moderating variables*

Data regarding the elevation of the populations was extracted directly from the study when such information was available. When the author failed to mention the elevation at which the populations were located but provided geographical coordinates for the populations, we entered the information in our database to use later for extraction of elevation (see below for methodology). In cases where the authors did not provide either elevation or geographic coordinates, we tried to manually georeference the locations based on the information available. If the description of the sites were too vague or could not be located with reasonable precision (~5km error) we discarded the study.

For studies where elevation was not provided but we managed to locate the sites, we extracted elevational data in the form of mean elevation within a certain buffer: for locations with GPS coordinates we used buffers of 1km around the central point, while for locations we manually georeferenced we used buffers of 5km. When we calculated averages for populations located at the same elevation and roughly in the same geographic area, or if the data was summarized by the authors as low – high, we created a minimum bounding geometry between all the population sites and used the centroid as the geographic location and the polygon for obtaining a mean elevation.

After obtaining elevation data for the low and high pairs, we calculated the variable **elevation range** (maximum elevation of the pair – minimum elevation of the pair) which we used as moderator to test if an increased separation in elevation corresponds to an increase in trait differentiation.

Not all populations from our database were located in close proximity to each other, which would lead to skewed results through the effect of latitudinal spacing; for reference, in our analysis of adult body size the location of population pairs ranged from 0 to 12.8° of latitude. Moreover, the effects of elevation do not scale linearly with latitude, which is why temperatures are higher for the same elevation in tropical regions and climatic conditions are more stable for tropical mountains compared to temperate counterparts (Janzen 1967; Ghalambor *et al.* 2006; Muñoz & Bodensteiner 2019). To account for the effect of latitude and the interaction between elevation and latitude, we included (along with elevation) the absolute value of the latitude difference between the low and high elevation populations as a product moderator in our meta-regressions (latitude × elevation).

In addition, there was variability among the extracted studies in the mean latitude of their populations. While most populations were located towards mid-latitude, there were also population pairs located polewards. Consequently, we checked for the influence of mean latitude using a single moderator (mean latitude) meta-regression and also by including mean latitude in the analyses as a covariate with elevation range as main effects along with their interaction.

Variables of latitudinal and elevational differences were log-transformed and scaled before running the meta-regressions, while the absolute value of mean latitude was included as is. Because some of the locations were close together, leading to zero values of latitude distance, which results in infinite values for log-transformed data, and there are different opinions on the best method for dealing with the problem, we opted to test two methods, namely (1) adding a value of one to all data and (2) calculating a value that is the quadrat of the first quantile divided by the third quantile (Stahel 2002). In the end we selected the quartile method which gave us the lower Akaike Information Criterion (AIC) for the meta-regression, but generally the difference between the two methods was less than four ∆ AIC.

*Publication bias*

We performed publication bias tests, in the form of funnel plots for visual inspection (Figures S12-S13), as well as meta-regressions, looking for (a) a small study effect (using Egger’s regression adapted for multilevel models) and (b) a time-lag bias or decline effect (Nakagawa *et al.* 2022; Nakagawa *et al.* 2023b), but only for traits where we also performed meta-regressions. First, we performed a single moderator meta-regression to assess the significance of the effect and then we continued with an all-in analysis to confirm the pattern (Nakagawa *et al.* 2022). For the time-lag bias analysis the publication year was mean-centered. When publication bias was confirmed, the results presented and discussed are the bias-corrected values of the intercept (β_0_) (Nakagawa *et al.* 2023b).

We only detected a time-lag bias for response ratios of neonate body size using a single moderator meta-regression (*t* = -2.379, *p* = 0.0265) and the pattern was confirmed with the all-in multilevel meta-regression (*t* = -2.2342, *p* = 0.0365). We did not perform publication bias tests for the coefficients of variation since we used the same data in the analyses.

Table S1 Search structure for the Google Scholar and Scopus databases, including keyword combinations used, total number of results, number of results consulted, and the number of papers retrieved

| **Keywords** | **No. of results** | **No. of results consulted** | **No. of articles saved** | **No. results**  **Scopus** |
| --- | --- | --- | --- | --- |
| “altitud*” OR “elevation*” AND “life history” AND “lizard” OR “snake” | 24500 | 1000 | 277 | 124 |
| “altitud*” OR “elevation*” AND “age” AND “lizard” OR “snake” | 123000 | 300 | 17 | 99 |
| “altitud*” OR “elevation*” AND “body mass” AND “lizard” OR “snake” | 10800 | 300 | 29 | 49 |
| “altitud*” OR “elevation*” AND “body size” AND “lizard” OR “snake” | 16900 | 300 | 12 | 191 |
| “altitud*” OR “elevation*” AND “reproduc*” AND “lizard” OR “snake” | 3330 | 400 | 10 | 237 |
| “altitud*” OR “elevation*” AND “breeding” AND “lizard” OR “snake” | 41700 | 200 | 3 | 50 |
| “altitud*” OR “elevation*” AND “clutch” AND “lizard” OR “snake” | 10300 | 200 | 3 | 64 |
| “altitud*” OR “elevation*” AND “egg” AND “lizard” OR “snake” | 34000 | 400 | 5 | 100 |
| “altitud*” OR “elevation*” AND “demograph*” AND “lizard” OR “snake” | 87 | 87 | 0 | 54 |
| “altitud*” OR “elevation*” AND “juvenile” AND “lizard” OR “snake” | 30400 | 300 | 6 | 67 |
| “altitud*” OR “elevation*” AND “offspring” AND “lizard” OR “snake” | 23800 | 400 | 13 | 47 |
| “altitud*” OR “elevation*” AND “survival” AND “lizard” OR “snake” | 58500 | 200 | 6 | 100 |
| “altitud*” OR “elevation*” AND “reproduc* success” AND “lizard” OR “snake” | 156 | 156 | 0 | 14 |
| “altitud*” OR “elevation*” AND “life history” AND “snake” | 16200 | 400 | 8 | 21 |
| “altitud*” OR “elevation*” AND “age” AND “snake” | 93200 | 300 | 0 | 48 |
| “altitud*” OR “elevation*” AND “body mass” AND “snake” | 6390 | 200 | 3 | 8 |
| “altitud*” OR “elevation*” AND “body size” AND “snake” | 9640 | 300 | 5 | 26 |
| “altitud*” OR “elevation*” AND “reproduc*” AND “snake” | 2400 | 300 | 0 | 43 |
| “altitud*” OR “elevation*” AND “breeding” AND “snake” | 29000 | 200 | 0 | 14 |
| “altitud*” OR “elevation*” AND “clutch” AND “snake” | 7200 | 300 | 1 | 7 |
| “altitud*” OR “elevation*” AND “age” AND “snake” | 93200 | 200 | 0 | 48 |
| “altitud*” OR “elevation*” AND “egg” AND “snake” | 25500 | 300 | 0 | 18 |
| “altitud*” OR “elevation*” AND “demograph*” AND “snake” | 69 | 69 | 0 | 19 |
| “altitud*” OR “elevation*” AND “juvenile” AND “snake” | 22300 | 200 | 1 | 24 |
| “altitud*” OR “elevation*” AND “offspring” AND “snake” | 18000 | 300 | 2 | 7 |
| “altitud*” OR “elevation*” AND “survival” AND “snake” | 43500 | 200 | 0 | 34 |
| “altitud*” OR “elevation*” AND “reproduc* success” AND “snake” | 108 | 108 | 0 | 2 |

**Articles used in the meta-analyses:**

Addis, E. A., Gangloff, E. J., Palacios, M. G., Carr, K. E., & Bronikowski, A. M. (2017). Merging the “Morphology–Performance–Fitness” Paradigm and Life-History Theory in the Eagle Lake Garter Snake Research Project. Integrative and Comparative Biology, 57(2), 423-435. doi:10.1093/icb/icx079

Adolph, S. C. (1990). Influence of Behavioral Thermoregulation on Microhabitat Use by Two Sceloporus Lizards. Ecology, 71(1), 315-327. doi:https://doi.org/10.2307/1940271

Alba, J. L. J., Mendoza, H. A. P., de la Vega, A. H. D., & Tobón, S. R. S. (2021). ANALYSIS OF NEONATAL GROWTH IN CAPTIVE DUSKY RATTLESNAKES (Crotalus triseriatus) FROM TWO POPULATIONS IN CENTRAL MEXICO. Revista Latinoamericana de Herpetología, 4(2), 65-73.

Alencar, L. R. V., Galdino, C. A. B., & Nascimento, L. B. (2012). Life History Aspects of Oxyrhopus trigeminus (Serpentes: Dipsadidae) from Two Sites in Southeastern Brazil. Journal of Herpetology, 46(1), 9-13, 15.

Altunışık, A., & Eksilmez, H. (2020). Age, growth and survival rate in two populations of Darevskia derjugini (Nikolsky, 1898) from different altitudes (Squamata: Sauria: Lacertidae). Animal Biology, 71(2), 135-149. doi:https://doi.org/10.1163/15707563-bja10025

Álvarez-Ruiz, L., Megía-Palma, R., Reguera, S., Ruiz, S., Zamora-Camacho, F. J., Figuerola, J., & Moreno-Rueda, G. (2018). Opposed elevational variation in prevalence and intensity of endoparasites and their vectors in a lizard. Current Zoology, 64(2), 197-204. doi:10.1093/cz/zoy002

Anderson, R. O., Alton, L. A., White, C. R., & Chapple, D. G. (2022). Ecophysiology of a small ectotherm tracks environmental variation along an elevational cline. Journal of Biogeography, 49(2), 405-415. doi:https://doi.org/10.1111/jbi.14311

Andrews, R. M., Méndez-de la Cruz, F. R., Cruz, M. V.-S., & Rodríguez-Romero, F. (1999). Field and Selected Body Temperatures of the Lizards Sceloporus aeneus and Sceloporus bicanthalis. Journal of Herpetology, 33(1), 93-100. doi:10.2307/1565547

Andrews, R. M., Qualls, C. P., & Rose, B. R. (1997). Effects of Low Temperature on Embryonic Development of Sceloporus Lizards. Copeia, 1997(4), 827-833. doi:10.2307/1447300

Arribas, O. J. (2009). Morphological variability of the Cantabro-Pyrenean populations of Zootoca vivipara (JACQUIN, 1787) with description of a new subspecies. Herpetozoa, 21(3/4), 123-146.

Artacho, P., Saravia, J., Perret, S., Bartheld, J. L., & Le Galliard, J.-F. (2017). Geographic variation and acclimation effects on thermoregulation behavior in the widespread lizard Liolaemus pictus. Journal of Thermal Biology, 63, 78-87. doi:https://doi.org/10.1016/j.jtherbio.2016.11.001

ATKINS, N., SWAIN, R., WAPSTRA, E., & JONES, S. M. (2007). Late stage deferral of parturition in the viviparous lizard Niveoscincus ocellatus (Gray 1845): implications for offspring quality and survival. Biological Journal of the Linnean Society, 90(4), 735-746. doi:10.1111/j.1095-8312.2007.00770.x

Atkins, Z. S., Clemann, N., Chapple, D. G., Edwards, A. M., Sinsch, U., Hantzschmann, A. M., . . . Robert, K. A. (2020). Demographic and life history variation in two sky-island populations of an endangered alpine lizard. Journal of Zoology, 310(1), 34-44. doi:https://doi.org/10.1111/jzo.12728

Avella, I., Calvete, J. J., Sanz, L., Wüster, W., Licata, F., Quesada-Bernat, S., . . . Martínez-Freiría, F. (2022). Interpopulational variation and ontogenetic shift in the venom composition of Lataste's viper (Vipera latastei, Boscá 1878) from northern Portugal. Journal of Proteomics, 263, 104613. doi:https://doi.org/10.1016/j.jprot.2022.104613

Ballinger, R. E. (1973). Comparative Demography of Two Viviparous Iguanid Lizards (Sceloporus Jarrovi and Sceloporus Poinsetti). Ecology, 54(2), 269-283. doi:https://doi.org/10.2307/1934336

Ballinger, R. E. (1979). Intraspecific Variation in demography and Life History of the Lizard, Sceloporus Jarrovi, Along an Altitudinal Gradient in Southeastern Arizona. Ecology, 60(5), 901-909. doi:https://doi.org/10.2307/1936858

Ballinger, R. E., Smith, G. R., & Nietfeldt, J. W. (1996). Elevational Variation in Age at Maturity in Sceloporus Jarrovi: An Experimental Evaluation. The Southwestern Naturalist, 41(2), 179-182.

Bashey, F., & Dunham, A. E. (1997). Elevational Variation in the Thermal Constraints on and Microhabitat Preferences of the Greater Earless Lizard Cophosaurus texanus. Copeia, 1997(4), 725-737. doi:10.2307/1447290

Beaupre, S. J. (1993). An Ecological Study of Oxygen Consumption in the Mottled Rock Rattlesnake, Crotalus lepidus lepidus, and the Black-Tailed Rattlesnake, Crotalus molossus molossus, from Two Populations. Physiological Zoology, 66(3), 437-454. doi:10.1086/physzool.66.3.30163702

Beaupre, S. J. (1995). Comparative Ecology of the Mottled Rock Rattlesnake, Crotalus lepidus, in Big Bend National Park. Herpetologica, 51(1), 45-56.

Beaupre, S. J. (1995). Effects of Geographically Variable Thermal Environment on Bioenergetics of Mottled Rock Rattlesnakes. Ecology, 76(5), 1655-1665. doi:https://doi.org/10.2307/1938166

Beaupre, S. J. (1996). Field Metabolic Rate, Water Flux, and Energy Budgets of Mottled Rock Rattlesnakes, Crotalus lepidus, from Two Populations. Copeia, 1996(2), 319-329. doi:10.2307/1446847

Belasen, A., Brock, K., Li, B., Chremou, D., Valakos, E., Pafilis, P., . . . Foufopoulos, J. (2017). Fine with heat, problems with water: microclimate alters water loss in a thermally adapted insular lizard. Oikos, 126(3), 447-457. doi:https://doi.org/10.1111/oik.03712

Beltrán, I., Durand, V., Loiseleur, R., & Whiting, M. J. (2020). Effect of early thermal environment on the morphology and performance of a lizard species with bimodal reproduction. Journal of Comparative Physiology B, 190(6), 795-809. doi:10.1007/s00360-020-01312-2

Beltrán, I., Loiseleur, R., Durand, V., & Whiting, M. J. (2020). Effects of early thermal environment on the behavior and learning of a lizard with bimodal reproduction. Behavioral Ecology and Sociobiology, 74(6), 73. doi:10.1007/s00265-020-02849-6

Beltrán, I., Perry, C., Degottex, F., & Whiting, M. J. (2021). Behavioral Thermoregulation by Mothers Protects Offspring from Global Warming but at a Cost. Physiological and Biochemical Zoology, 94(5), 302-318. doi:10.1086/715976

Benabib, M. (1994). Reproduction and Lipid Utilization of Tropical Populations of Sceloporus variabilis. Herpetological Monographs, 8, 160-180. doi:10.2307/1467079

Benabib, M., & Congdon, J. D. (1992). Metabolic and Water-Flux Rates of Free-Ranging Tropical Lizards Sceloporus variabilis. Physiological Zoology, 65(4), 788-802. doi:10.1086/physzool.65.4.30158539

Beser, N., Ilgaz, Ç., Kumlutaş, Y., Candan, K., Güçlü, Ö., & Üzüm, N. (2020). Age and growth in two populations of danford’s lizard, anatololacerta danfordi (Günther, 1876), from the eastern mediterranean. Turkish Journal of Zoology, 44(2), 173-180. doi:10.3906/zoo-1909-39

Beuchat, C. A. (1986). Reproductive Influences on the Thermoregulatory Behavior of a Live-Bearing Lizard. Copeia, 1986(4), 971-979. doi:10.2307/1445294

Beuchat, C. A., & Ellner, S. (1987). A Quantitative Test of Life History Theory: Thermoregulation by a Viviparous Lizard. Ecological Monographs, 57(1), 45-60. doi:https://doi.org/10.2307/1942638

Bock, B. C., Ortega, A. M., Zapata, A. M., & Páez, V. P. (2009). Microgeographic body size variation in a high elevation Andean anole (Anolis mariarum; Squamata, Polychrotidae). Revista de Biología Tropical, 57(4), 1253-1262.

Bodensteiner, B. L., Gangloff, E. J., Kouyoumdjian, L., Muñoz, M. M., & Aubret, F. (2021). Thermal–metabolic phenotypes of the lizard Podarcis muralis differ across elevation, but converge in high-elevation hypoxia. Journal of Experimental Biology, 224(24). doi:10.1242/jeb.243660

Bronikowski, A., & Vleck, D. (2010). Metabolism, Body Size and Life Span: A Case Study in Evolutionarily Divergent Populations of the Garter Snake (Thamnophis elegans). Integrative and Comparative Biology, 50(5), 880-887. doi:10.1093/icb/icq132

Bronikowski, A. M. (2000). Experimental evidence for the adaptive evolution of growth rate in the garter snake Thamnophis elegans. Evolution, 54(5), 1760-1767. doi:10.1111/j.0014-3820.2000.tb00719.x

Bronikowski, A. M., & Arnold, S. J. (1999). THE EVOLUTIONARY ECOLOGY OF LIFE HISTORY VARIATION IN THE GARTER SNAKE THAMNOPHIS ELEGANS. Ecology, 80(7), 2314-2325. doi:https://doi.org/10.1890/0012-9658(1999)080[2314:TEEOLH]2.0.CO;2

Brown, C. K., & Ruby, D. E. (1977). Sex-Associated Variation in the Frequencies of Tail Autotomy in Sceloporus jarrovi (Sauria: Iguanidae) at Different Elevations. Herpetologica, 33(3), 380-387.

Brown, R. P. (1996). Thermal Biology of the Gecko Tarentola boettgeri: Comparisons among Populations from Different Elevations within Gran Canaria. Herpetologica, 52(3), 396-405.

Brusch, G. A., IV, Gavira, R. S. B., Viton, R., Dupoué, A., Leroux-Coyau, M., Meylan, S., . . . Lourdais, O. (2020). Additive effects of temperature and water availability on pregnancy in a viviparous lizard. Journal of Experimental Biology, 223(19). doi:10.1242/jeb.228064

BUCKLEY, C. R., IRSCHICK, D. J., & ADOLPH, S. C. (2009). The contributions of evolutionary divergence and phenotypic plasticity to geographic variation in the western fence lizard, Sceloporus occidentalis. Biological Journal of the Linnean Society, 99(1), 84-98. doi:10.1111/j.1095-8312.2009.01346.x

Buckley, C. R., Jackson, M., Youssef, M., Irschick, D. J., & Adolph, S. C. (2007). Testing the persistence of phenotypic plasticity after incubation in the western fence lizard, Sceloporus occidentalis. Evolutionary Ecology Research, 9(1), 169-183.

Bula, P. A., Wright, L. K., & Zani, P. A. (2015). Geographic variation in lizard hind-limb morphology in relation to predation: no evidence for an evolutionary basis. Evolutionary Ecology Research, 16(8), 663-687.

Bülbül, U., Kurnaz, M., Eroğlu, A. İ., Koç, H., & Kutrup, B. (2016). Age and growth of the red-belied lizard, Darevskia parvula. Animal Biology, 66(1), 81-95. doi:https://doi.org/10.1163/15707563-00002489

Bülbül, U., Kurnaz, M., Eroğlu, A. İ., Koç, H., & Kutrup, B. (2016). Body size and age structure of the endangered Clark’s lizard (Darevskia clarkorum) populations from two different altitudes in Turkey. Amphibia-Reptilia, 37(4), 450-456. doi:https://doi.org/10.1163/15685381-00003073

Burraco, P., Comas, M., Reguera, S., Zamora-Camacho, F. J., & Moreno-Rueda, G. (2020). Telomere length mirrors age structure along a 2200-m altitudinal gradient in a Mediterranean lizard. Comparative Biochemistry and Physiology Part A: Molecular & Integrative Physiology, 247, 110741. doi:https://doi.org/10.1016/j.cbpa.2020.110741

Cadby, C. D., Jones, S. M., & Wapstra, E. (2011). Potentially adaptive effects of maternal nutrition during gestation on offspring phenotype of a viviparous reptile. Journal of Experimental Biology, 214(24), 4234-4239. doi:10.1242/jeb.057349

Cadby, C. D., Jones, S. M., & Wapstra, E. (2014). Geographical differences in maternal basking behaviour and offspring growth rate in a climatically widespread viviparous reptile. Journal of Experimental Biology, 217(7), 1175-1179. doi:10.1242/jeb.089953

CADBY, C. D., WHILE, G. M., HOBDAY, A. J., ULLER, T., & WAPSTRA, E. (2010). Multi-scale approach to understanding climate effects on offspring size at birth and date of birth in a reptile. Integrative Zoology, 5(2), 164-175. doi:https://doi.org/10.1111/j.1749-4877.2010.00201.x

Calderón-Espinosa, M. L., Andrews, R. M., & Méndez de la Cruz, F. R. (2006). EVOLUTION OF EGG RETENTION IN THE SCELOPORUS SPINOSUS GROUP: EXPLORING THE ROLE OF PHYSIOLOGICAL, ENVIRONMENTAL, AND PHYLOGENETIC FACTORS. Herpetological Monographs, 20(1), 147-158. doi:10.1655/0733-1347(2007)20[147:Eoerit]2.0.Co;2

Caldwell, A. J., While, G. M., & Wapstra, E. (2017). Plasticity of thermoregulatory behaviour in response to the thermal environment by widespread and alpine reptile species. Animal Behaviour, 132, 217-227. doi:https://doi.org/10.1016/j.anbehav.2017.07.025

Caley, M. J., & Schwarzkopf, L. (2004). COMPLEX GROWTH RATE EVOLUTION IN A LATITUDINALLY WIDESPREAD SPECIES. Evolution, 58(4), 862-869. doi:10.1111/j.0014-3820.2004.tb00417.x

Candan, K. (2021). Body size and age structure of Lacerta agilis LINNAEUS, 1758 (Reptilia: Lacertidae) from two different populations in Turkey. Biological Diversity and Conservation, 14(3), 505-510.

Capula, M., Filippi, E., Luiselli, L., Aguilar, J. R., & Rugiero, L. (2000). Body Size and Some Demographic Characteristics in Two Populations of Coluber viridiflavus in the Countryside of Rome. Mus. reg. Sci. nat. Torino, 435-438.

Capula, M., & Luiselli, L. (2002). Feeding strategies of Elaphe longissima from contrasting Mediterranean habitats in central Italy. Italian Journal of Zoology, 69(2), 153-156. doi:10.1080/11250000209356453

Capula, M., Luiselli, L., & Rugiero, L. (1995). Ecological correlates of reproductive mode in reproductively bimodal snakes of the genus Coronella. Vie et Milieu, 45(2), 167-175.

Cardona-Botero, V. E., Lara-Reséndiz, R. A., Woolrich-Piña, G. A., Pineda, E., Lira-Noriega, A., & Gadsden, H. (2023). Seasonal and elevational variation in thermal ecology of the crevice-dwelling knob-scaled lizard Xenosaurus fractus from central-eastern Mexico. Journal of Thermal Biology, 112, 103432. doi:https://doi.org/10.1016/j.jtherbio.2022.103432

Cardozo, G., & Chiaraviglio, M. (2011). Phenotypic plasticity of life history traits in relation to reproductive strategies in Boa constrictor occidentalis. Evolutionary Ecology, 25(5), 1163-1177. doi:10.1007/s10682-011-9465-y

Cecchetto, N. R., Medina, S. M., & Ibargüengoytía, N. R. (2020). Running performance with emphasis on low temperatures in a Patagonian lizard, Liolaemus lineomaculatus. Scientific Reports, 10(1), 14732. doi:10.1038/s41598-020-71617-3

Chabaud, C., Berroneau, M., Berroneau, M., Dupoué, A., Guillon, M., Viton, R., . . . Le Galliard, J. F. (2022). Climate aridity and habitat drive geographical variation in morphology and thermo-hydroregulation strategies of a widespread lizard species. Biological Journal of the Linnean Society, 137(4), 667-685. doi:10.1093/biolinnean/blac114

Chamorro-Vargas, C. T., Perez-Rojas, S., Garcia, U. S. R., Rodríguez Rodríguez, J. D., Castillo-Rivera, J., & Méndez-Galeano, M. Á. (2021). Living in a cold tropical mountain: do the microhabitat use and activity pattern change with elevation in the high-Andean lizard Stenocercus trachycephalus (Squamata: Tropiduridae)? Papéis Avulsos de Zoologia, 61, e20216170.

Claunch, N. M., Nix, E., Royal, A. E., Burgos, L. P., Corn, M., DuBois, P. M., . . . Taylor, E. N. (2021). Body size impacts critical thermal maximum measurements in lizards. Journal of Experimental Zoology Part A: Ecological and Integrative Physiology, 335(1), 96-107. doi:https://doi.org/10.1002/jez.2410

Comas, M. (2020). Body condition, sex and elevation in relation to mite parasitism in a high mountain gecko. Journal of Zoology, 310(4), 298-305. doi:https://doi.org/10.1111/jzo.12751

Comas, M., Reguera, S., Zamora-Camacho, F. J., & Moreno-Rueda, G. (2019). Age structure of a lizard along an elevational gradient reveals nonlinear lifespan patterns with altitude. Current Zoology, 66(4), 373-382. doi:10.1093/cz/zoz063

Combrink, L. L., Bronikowski, A. M., Miller, D. A. W., & Sparkman, A. M. (2021). Current and time-lagged effects of climate on innate immunity in two sympatric snake species. Ecology and evolution, 11(7), 3239-3250. doi:https://doi.org/10.1002/ece3.7273

Conover, A. E., Cook, E. G., Boronow, K. E., & Muñoz, M. M. (2015). Effects of Ectoparasitism on Behavioral Thermoregulation in the Tropical lizards Anolis cybotes (Squamata: Dactyloidae) and Anolis armouri (Squamata: Dactyloidae). Breviora, 545(1), 1-13, 13.

Cree, A., & Guillette, L. J. (1995). Biennial Reproduction with a Fourteen-Month Pregnancy in the Gecko Hoplodactylus maculatus from Southern New Zealand. Journal of Herpetology, 29(2), 163-173. doi:10.2307/1564553

Cree, A., & Hare, K. M. (2016). Reproduction and Life History of New Zealand Lizards. In D. G. Chapple (Ed.), New Zealand Lizards (pp. 169-206). Cham: Springer International Publishing.

Crnobrnja-Isailovic, J., & Aleksic, I. (2004). Clutch size in two Central Balkan populations of European common lizard Lacerta vivipara. Biota, 5, 5-10.

Crowley, S. R. (1985). Insensitivity to Desiccation of Sprint Running Performance in the Lizard, Sceloporus undulatus. Journal of Herpetology, 19(1), 171-174. doi:10.2307/1564437

Crowley, S. R. (1985). Thermal sensitivity of sprint-running in the lizard Sceloporus undulatus: support for a conservative view of thermal physiology. Oecologia, 66(2), 219-225. doi:10.1007/BF00379858

Cruz-Elizalde, R., & Ramírez-Bautista, A. (2016). Reproductive cycles and reproductive strategies among populations of the Rose-bellied Lizard Sceloporus variabilis (Squamata: Phrynosomatidae) from central Mexico. Ecology and evolution, 6(6), 1753-1768. doi:https://doi.org/10.1002/ece3.1998

Cruz-Elizalde, R., Ramírez-Bautista, A., Hernández-Salinas, U., Diáz-Marín, C. A., Marshall, J. C., Sites, J. W., . . . Berriozabal-Islas, C. (2023). Variation in body size and reproductive characteristics among chromosomal races of the Sceloporus grammicus complex in Mexico. Biological Journal of the Linnean Society, 138(4), 392-412. doi:10.1093/biolinnean/blac150

Cruz-Elizalde, R., Ramírez-Bautista, A., & Lozano, A. (2017). Sexual size dimorphism among populations of the rose-bellied lizard Sceloporus variabilis (Squamata: Phrynosomatidae) from high and low elevations in Mexico. Herpetological Journal, 27(3).

Cruz-Elizalde, R., Ramírez-Bautista, A., Rosas Pacheco, L. F., Lozano, A., & Rodríguez-Romero, F. d. J. (2020). Sexual dimorphism in size and shape among populations of the lizard Sceloporus variabilis (Squamata: Phrynosomatidae). Zoology, 140, 125781. doi:https://doi.org/10.1016/j.zool.2020.125781

Čubrić, T., Bonnet, X., & Crnobrnja‐Isailović, J. (2023). Body size and body condition in the nose-horned viper (Vipera ammodytes): effects of sex and populations. Herpetozoa, 36, 53-58.

Cunningham, G. D., While, G. M., Olsson, M., Ljungström, G., & Wapstra, E. (2020). Degrees of change: between and within population variation in thermal reaction norms of phenology in a viviparous lizard. Ecology, 101(10), e03136. doi:https://doi.org/10.1002/ecy.3136

Cunningham, G. D., While, G. M., & Wapstra, E. (2017). Climate and sex ratio variation in a viviparous lizard. Biology Letters, 13(5), 20170218. doi:doi:10.1098/rsbl.2017.0218

Dayananda, B., Gray, S., Pike, D., & Webb, J. K. (2016). Communal nesting under climate change: fitness consequences of higher incubation temperatures for a nocturnal lizard. Global change biology, 22(7), 2405-2414. doi:https://doi.org/10.1111/gcb.13231

Dayananda, B., Ibargüengoytía, N., Whiting, M. J., & Webb, J. K. (2017). Effects of pregnancy on body temperature and locomotor performance of velvet geckos. Journal of Thermal Biology, 65, 64-68. doi:https://doi.org/10.1016/j.jtherbio.2017.02.005

Deme, G. G., Hao, X., Ma, L., Sun, B., & Du, W. (2022). Elevational variation in reproductive strategy of a widespread lizard: High-elevation females lay fewer but larger eggs. Asian Herpetol. Res, 198-204.

Díaz, J. A. (1997). Ecological correlates of the thermal quality of an ectotherm’s habitat: a comparison between two temperate lizard populations. Functional Ecology, 11(1), 79-89. doi:https://doi.org/10.1046/j.1365-2435.1997.00058.x

Díaz, J. A., Iraeta, P., & Monasterio, C. (2006). Seasonality provokes a shift of thermal preferences in a temperate lizard, but altitude does not. Journal of Thermal Biology, 31(3), 237-242. doi:https://doi.org/10.1016/j.jtherbio.2005.10.001

Díaz, J. A., Iraeta, P., Verdú-Ricoy, J., Siliceo, I., & Salvador, A. (2012). Intraspecific Variation of Reproductive Traits in a Mediterranean Lizard: Clutch, Population, and Lineage Effects. Evolutionary Biology, 39(1), 106-115. doi:10.1007/s11692-011-9144-5

Diego-Rasilla, F. J. (2003). Influence of predation pressure on the escape behaviour of Podarcis muralis lizards. Behavioural Processes, 63(1), 1-7. doi:https://doi.org/10.1016/S0376-6357(03)00026-3

Dissanayake, D. S. B., Holleley, C. E., Deakin, J. E., & Georges, A. (2021). High elevation increases the risk of Y chromosome loss in Alpine skink populations with sex reversal. Heredity, 126(5), 805-816. doi:10.1038/s41437-021-00406-z

Dissanayake, D. S. B., Holleley, C. E., & Georges, A. (2021). Effects of natural nest temperatures on sex reversal and sex ratios in an Australian alpine skink. Scientific Reports, 11(1), 20093. doi:10.1038/s41598-021-99702-1

Domínguez-Godoy, M. A., Gómez-Campos, J. E., Hudson, R., & Díaz de la Vega-Pérez, A. H. (2020). Lower Predation with Increasing Altitude in the Mesquite Lizard Sceloporus grammicus. Western North American Naturalist, 80(4), 441-451, 411.

Domínguez-Godoy, M. A., Hudson, R., Pérez-Mendoza, H. A., Ancona, S., & Díaz de la Vega-Pérez, A. H. (2020). Living on the edge: Lower thermal quality but greater survival probability at a high altitude mountain for the mesquite lizard (Sceloporus grammicus). Journal of Thermal Biology, 94, 102757. doi:https://doi.org/10.1016/j.jtherbio.2020.102757

DOODY, J. S. (2009). Superficial lizards in cold climates: Nest site choice along an elevational gradient. Austral Ecology, 34(7), 773-779. doi:https://doi.org/10.1111/j.1442-9993.2009.01983.x

Doody, J. S., Guarino, E., Georges, A., Corey, B., Murray, G., & Ewert, M. (2006). Nest site choice compensates for climate effects on sex ratios in a lizard with environmental sex determination. Evolutionary Ecology, 20(4), 307-330. doi:10.1007/s10682-006-0003-2

DU, W., ROBBINS, T. R., WARNER, D. A., LANGKILDE, T., & SHINE, R. (2014). Latitudinal and seasonal variation in reproductive effort of the eastern fence lizard (Sceloporus undulatus). Integrative Zoology, 9(3), 360-371. doi:https://doi.org/10.1111/1749-4877.12072

Du, W.-G., Elphick, M., & Shine, R. (2010). Thermal regimes during incubation do not affect mean selected temperatures of hatchling lizards (Bassiana duperreyi, Scincidae). Journal of Thermal Biology, 35(1), 47-51. doi:https://doi.org/10.1016/j.jtherbio.2009.10.007

Du, W.-G., Ji, X., Zhang, Y.-P., Xu, X.-F., & Shine, R. (2005). Identifying sources of variation in reproductive and life-history traits among five populations of a Chinese lizard (Takydromus septentrionalis, Lacertidae). Biological Journal of the Linnean Society, 85(4), 443-453. doi:10.1111/j.1095-8312.2005.00508.x

Dubey, S., Sinsch, U., Dehling, M. J., Chevalley, M., & Shine, R. (2013). Population demography of an endangered lizard, the Blue Mountains Water Skink. BMC Ecology, 13(1), 4. doi:10.1186/1472-6785-13-4

DuBois, P. M., Shea, T. K., Claunch, N. M., & Taylor, E. N. (2017). Effects of oxygen on responses to heating in two lizard species sampled along an elevational gradient. Journal of Thermal Biology, 68, 170-176. doi:https://doi.org/10.1016/j.jtherbio.2017.02.008

Dunham, A. E. (1982). Demographic and Life-History Variation among Populations of the Iguanid Lizard Urosaurus ornatus: Implications for the Study of Life-History Phenomena in Lizards. Herpetologica, 38(1), 208-221.

Dupoué, A., Rutschmann, A., Le Galliard, J. F., Clobert, J., Blaimont, P., Sinervo, B., . . . Meylan, S. (2018). Reduction in baseline corticosterone secretion correlates with climate warming and drying across wild lizard populations. Journal of Animal Ecology, 87(5), 1331-1341. doi:10.1111/1365-2656.12843

Dupoué, A., Sorlin, M., Richard, M., Le Galliard, J. F., Lourdais, O., Clobert, J., & Aubret, F. (2020). Mother-offspring conflict for water and its mitigation in the oviparous form of the reproductively bimodal lizard, Zootoca vivipara. Biological Journal of the Linnean Society, 129(4), 888-900. doi:10.1093/biolinnean/blaa012

Dyugmedzhiev, A., Naumov, B., & Tzankov, N. (2021). Thermal ecology of the Nose-Horned Viper (Vipera ammodytes (Linnaeus, 1758)) under natural conditions. North-Western Journal of Zoology, 17(1).

Dyugmedzhiev, A. V., Popgeorgiev, G. S., Tzankov, N. D., & Naumov, B. Y. (2020). Population estimates of the Nose-horned Viper Vipera ammodytes (Linnaeus, 1758)(Reptilia: Viperidae) from five populations in Bulgaria. Acta Zoologica Bulgarica, 72(3), 397-407.

Eroğlu, A. İ., Bülbül, U., Kurnaz, M., & Odabaş, Y. (2018). Age and growth of the common wall lizard, Podarcis muralis (Laurenti, 1768). Animal Biology, 68(2), 147-159. doi:https://doi.org/10.1163/15707563-17000019

Ferguson, G. W., & Talent, L. G. (1993). Life-history traits of the lizard Sceloporus undulatus from two populations raised in a common laboratory environment. Oecologia, 93(1), 88-94. doi:10.1007/BF00321196

Fierro-Estrada, N., González González, Y. G., Miles, D. B., Martínez Gómez, M., García, A., Salgado-Ugarte, I. H., & Méndez de la Cruz, F. R. (2019). Thermoregulation of the lizard Barisia imbricata at altitudinal extremes. Amphibia-Reptilia, 40(3), 349-360. doi:https://doi.org/10.1163/15685381-20191155

Filippakopoulou, A., Santos, X., Feriche, M., Pleguezuelos, J. M., & Llorente, G. A. (2014). Effect of prey availability on growth-rate trajectories of an aquatic predator, the viperine snake Natrix maura. Basic and Applied Herpetology, 28, 35-50.

Fischer, J., & Lindenmayer, D. B. (2005). The sensitivity of lizards to elevation: A case study from south-eastern Australia. Diversity and Distributions, 11(3), 225-233. doi:https://doi.org/10.1111/j.1366-9516.2005.00139.x

Fornasiero, S., Bonnet, X., Dendi, F., & Zuffi, M. (2016). Growth, longevity and age at maturity in the European whip snakes, Hierophis viridiflavus and H. carbonarius. Acta Herpetologica, 11(2), 135-149.

Fornasiero, S., Dendi, F., Bresciani, E., Cecchinelli, E., & Zuffi, M. (2011). The scent of the others: chemical recognition in two distinct populations of the European whip snake, Hierophis viridiflavus. Amphibia-Reptilia, 32(1), 39-47. doi:https://doi.org/10.1163/017353710X541850

Fox, S. F., Perea-Fox, S., & Franco, R. C. (1994). Development of the Tail Autotomy Adaptation in Lizards under Disparate Levels of Predation at High and Low Elevations in Mexico. The Southwestern Naturalist, 39(4), 311-322.

Gabirot, M., Balleri, A., López, P., & Martín, J. (2013). Differences in Thermal Biology Between Two Morphologically Distinct Populations of Iberian Wall Lizards Inhabiting Different Environments. Annales Zoologici Fennici, 50(4), 225-236, 212.

Galoyan, E., Bolshakova, A., Abrahamyan, M., Petrosyan, R., Komarova, V., Viсtor, S., & Marine, A. (2019). Natural history of Valentin’s rock lizard (Darevskia valentini) in Armenia. Zoological research, 40(4), 277.

Gangloff, E. J., Chow, M., Leos-Barajas, V., Hynes, S., Hobbs, B., & Sparkman, A. M. (2017). Integrating behaviour into the pace-of-life continuum: Divergent levels of activity and information gathering in fast- and slow-living snakes. Behavioural Processes, 142, 156-163. doi:https://doi.org/10.1016/j.beproc.2017.06.006

Gangloff, E. J., Holden, K. G., Telemeco, R. S., Baumgard, L. H., & Bronikowski, A. M. (2016). Hormonal and metabolic responses to upper temperature extremes in divergent life-history ecotypes of a garter snake. Journal of Experimental Biology, 219(18), 2944-2954. doi:10.1242/jeb.143107

Gangloff, E. J., Manes, M. B., Schwartz, T. S., Robert, K. A., Huebschman, N., & Bronikowski, A. M. (2021). Multiple Paternity in Garter Snakes With Evolutionarily Divergent Life Histories. Journal of Heredity, 112(6), 508-518. doi:10.1093/jhered/esab043

Gangloff, E. J., Schwartz, T. S., Klabacka, R., Huebschman, N., Liu, A.-Y., & Bronikowski, A. M. (2020). Mitochondria as central characters in a complex narrative: Linking genomics, energetics, pace-of-life, and aging in natural populations of garter snakes. Experimental Gerontology, 137, 110967. doi:https://doi.org/10.1016/j.exger.2020.110967

Gangloff, E. J., Sparkman, A. M., Holden, K. G., Corwin, C. J., Topf, M., & Bronikowski, A. M. (2017). Geographic variation and within-individual correlations of physiological stress markers in a widespread reptile, the common garter snake (Thamnophis sirtalis). Comparative Biochemistry and Physiology Part A: Molecular & Integrative Physiology, 205, 68-76. doi:https://doi.org/10.1016/j.cbpa.2016.12.019

Gangloff, E. J., Vleck, D., & Bronikowski, A. M. (2015). Developmental and Immediate Thermal Environments Shape Energetic Trade-Offs, Growth Efficiency, and Metabolic Rate in Divergent Life-History Ecotypes of the Garter Snake Thamnophis elegans. Physiological and Biochemical Zoology, 88(5), 550-563. doi:10.1086/682239

Gao, J., Wei, Z., & Jin, Y. (2024). The impact of elevation and prediction of climate change on an ultra high-elevation ectotherm. Ecology and evolution, 14(9). doi:10.1002/ece3.70186

Garcia, C. M., & Drummond, H. (1990). Population Differences in Fish-Capturing Ability of the Mexican Aquatic Garter Snake (Thamnophis melanogaster). Journal of Herpetology, 24(4), 412-416. doi:10.2307/1565061

García-Rosales, A., Cruz-Elizalde, R., Ramírez-Bautista, A., & Hernández-Camacho, N. (2024). Caudal autotomy among populations of the lizard Sceloporus variabilis (Squamata: Phrynosomatidae) in contrasting environments. Acta Zoologica, 105(3), 366-376. doi:https://doi.org/10.1111/azo.12478

García-Rosales, A., Cruz-Elizalde, R., Ramírez-Bautista, A., & Mata-Silva, V. (2019). Feeding ecology of two populations of Sceloporus minor (Squamata: Phrynosomatidae) inhabiting contrasting environments in central Mexico. Salamandra, 55(2).

Gilbert, A. L., & Miles, D. B. (2019). Spatiotemporal variation in thermal niches suggests lability rather than conservatism of thermal physiology along an environmental gradient. Biological Journal of the Linnean Society, 128(2), 263-277. doi:10.1093/biolinnean/blz093

Goldberg, S. R. (1974). Reproduction in Mountain and Lowland Populations of the Lizard Sceloporus occidentalis. Copeia, 1974(1), 176-182. doi:10.2307/1443021

Gomez, L., Larsen, K. W., & Gregory, P. T. (2015). Contrasting Patterns of Migration and Habitat Use in Neighboring Rattlesnake Populations. Journal of Herpetology, 49(3), 371-376, 376.

González-Morales, J. C., Beamonte-Barrientos, R., Bastiaans, E., Guevara-Fiore, P., Quintana, E., & Fajardo, V. (2017). A Mountain or a Plateau? Hematological Traits Vary Nonlinearly with Altitude in a Highland Lizard. Physiological and Biochemical Zoology, 90(6), 638-645. doi:10.1086/694833

González-Morales, J. C., Fajardo, V., de la Vega-Pérez, A. H. D., Barrios-Montiel, R., Quintana, E., Moreno-Rueda, G., . . . Bastiaans, E. (2023). Elevation and blood traits in the mesquite lizard: Are patterns repeatable between mountains? Comparative Biochemistry and Physiology Part A: Molecular & Integrative Physiology, 276, 111338. doi:https://doi.org/10.1016/j.cbpa.2022.111338

González-Morales, J. C., Quintana, E., Díaz-Albiter, H., Guevara-Fiore, P., & Fajardo, V. (2015). Is erythrocyte size a strategy to avoid hypoxia in Wiegmann’s Torquate Lizards (Sceloporus torquatus)? Field evidence. Canadian Journal of Zoology, 93(5), 377-382. doi:10.1139/cjz-2014-0265

González-Morales, J. C., Rivera-Rea, J., Moreno-Rueda, G., Bastiaans, E., Castro-López, M., & Fajardo, V. (2021). Fast and dark: The case of Mezquite lizards at extreme altitude. Journal of Thermal Biology, 102, 103115. doi:https://doi.org/10.1016/j.jtherbio.2021.103115

González-Morales, J. C., Rivera-Rea, J., Moreno-Rueda, G., Bastiaans, E., Díaz-Albiter, H., Díaz de la Vega-Pérez, A. H., . . . Fajardo, V. (2020). To be small and dark is advantageous for gaining heat in mezquite lizards, Sceloporus grammicus (Squamata: Phrynosomatidae). Biological Journal of the Linnean Society, 132(1), 93-103. doi:10.1093/biolinnean/blaa176

González-Morales, J. C., Rivera-Rea, J., Moreno-Rueda, G., Plasman, M., Quintana, E., & Bastiaans, E. (2024). Seasonal and altitudinal variation in dorsal skin reflectance and thermic rates in a high-altitude montane lizard. International Journal of Biometeorology. doi:10.1007/s00484-024-02677-7

Goode, M., & Parker, M. R. (2011). Microgeographic variation in Tiger Rattlesnake ecology and life history: the importance of long-term, natural history-based, multiple-population research. Reptiles & Amphibians, 18(2), 84-91.

Grant, B. W., & Dunham, A. E. (1990). Elevational Covariation in Environmental Constraints and Life Histories of the Desert Lizard Sceloporus Merriami. Ecology, 71(5), 1765-1776. doi:https://doi.org/10.2307/1937584

Gregory, P. T., & Larsen, K. W. (1993). Geographic Variation in Reproductive Characteristics among Canadian Populations of the Common Garter Snake (Thamnophis sirtalis). Copeia, 1993(4), 946-958. doi:10.2307/1447072

Gregory, P. T., & Larsen, K. W. (1996). Are There Any Meaningful Correlates of Geographic Life-History Variation in the Garter Snake, Thamnophis sirtalis? Copeia, 1996(1), 183-189. doi:10.2307/1446954

Güizado-Rodríguez, M. A., Casas-Andreu, G., López-Alcaide, S., & Cruz-Aviña, J. R. (2024). Inter-population thermal ecology of the Mexican endemic whiptail lizard Aspidoscelis costatus costatus (Cope, 1878). Herpetology Notes, 17, 101-107.

Gül, S., Özdemir, N., Avci, A., Kumlutaş, Y., & Ilgaz, C. (2015). Altitudinal effects on the life history of the Anatolian lizard (Apathya cappadocica, Werner 1902) from southeastern Anatolia, Turkey. Turkish Journal of Zoology, 39(3), 507-512.

Gül, S., Özdemir, N., Kumlutaş, Y., Durmuş, S. H., & Ilgaz, Ç. (2015). Age structure and body size variation in populations of Darevskia bithynica (Méhely, 1909)(Reptilia: Lacertidae) from different altitudes in north-western Turkey.

Gül, S., Özdemir, N., Kumlutaş, Y., & Ilgaz, C. (2014). Age structure and body size in three populations of Darevskia rudis (Bedriaga, 1886) from different altitudes. Herpetozoa, 26, 151-158.

Gutiérrez, J. A., Krenz, J. D., & Ibargüengoytía, N. R. (2010). Effect of altitude on thermal responses of Liolaemus pictus argentinus in Argentina. Journal of Thermal Biology, 35(7), 332-337. doi:https://doi.org/10.1016/j.jtherbio.2010.07.001

Gutiérrez, J. A., Piantoni, C., & Ibargüengoytía, N. (2013). Altitudinal effects on life history parameters in populations of Liolaemus pictus argentinus (Sauria: Liolaemidae). Acta Herpetologica, 8, 9-17.

Gvoždík, L. (2002). To heat or to save time? Thermoregulation in the lizard Zootoca vivipara (Squamata: Lacertidae) in different thermal environments along an altitudinal gradient. Canadian Journal of Zoology, 80(3), 479-492. doi:10.1139/z02-015

Gvoždík, L., & Castilla, A. M. (2001). A Comparative Study of Preferred Body Temperatures and Critical Thermal Tolerance Limits among Populations of Zootoca vivipara (Squamata: Lacertidae) along an Altitudinal Gradient. Journal of Herpetology, 35(3), 486-492. doi:10.2307/1565967

Haenel, G. (2011). Effects of Habitat on Clutch Size of Ornate Tree Lizards, Urosaurus ornatus. Western North American Naturalist, 71(2), 247-256, 210.

Han, J., Guo, R., Li, J., Guan, C., Chen, Y., & Zhao, W. (2016). Organ Mass Variation in a Toad Headed Lizard Phrynocephalus vlangalii in Response to Hypoxia and Low Temperature in the Qinghai-Tibet Plateau, China. Plos One, 11(9), e0162572. doi:10.1371/journal.pone.0162572

Hao, X., Wang, C.-X., Han, X.-Z., Wang, Y., Zhang, Q., Zhang, F.-S., . . . Du, W.-G. (2021). A reciprocal egg-swap experiment reveals sources of variation in developmental success among populations of a desert lizard. Oecologia, 196(1), 27-35. doi:10.1007/s00442-021-04903-0

Hernández-Amparan, S., Sainz-Mellado, I., Hernández-Salinas, U., & López-González, C. (2021). TESTING BERGMANN'S RULE IN THE WIDESPREAD MEXICAN LIZARD ANOLIS NEBULOSUS (SQUAMATA: DACTYLOIDAE). The Southwestern Naturalist, 65(1), 1-8, 8.

Hernández-Salinas, U., Ramírez-Bautista, A., Leyte-Manrique, A., & Smith, G. R. (2010). Reproduction and Sexual Dimorphism in Two Populations of Sceloporus grammicus (Sauria: Phrynosomatidae) from Hidalgo, Mexico. Herpetologica, 66(1), 12-22, 11.

Herrando-Pérez, S., Ferri-Yáñez, F., Monasterio, C., Beukema, W., Gomes, V., Belliure, J., . . . Araújo, M. B. (2019). Intraspecific variation in lizard heat tolerance alters estimates of climate impact. Journal of Animal Ecology, 88(2), 247-257. doi:https://doi.org/10.1111/1365-2656.12914

Hertz, P. E. (1980). Responses to Dehydration in Anolis Lizards Sampled along Altitudinal Transects. Copeia, 1980(3), 440-446. doi:10.2307/1444519

Hertz, P. E. (1981). Adaptation to altitude in two West Indian anoles (Reptilia. Journal of Zoology, 195(1), 25-37. doi:https://doi.org/10.1111/j.1469-7998.1981.tb01891.x

Hertz, P. E. (1992). Temperature Regulation in Puerto Rican Anolis Lizards: A Field Test Using Null Hypotheses. Ecology, 73(4), 1405-1417. doi:10.2307/1940686

Hertz, P. E., Arce-Hernandez, A., Ramirez-Vazquez, J., Tirado-Rivera, W., & Vazquez-Vives, L. (1979). Geographical variation of heat sensitivity and water loss rates in the tropical lizard, Anolis gundlachi. Comparative Biochemistry and Physiology Part A: Physiology, 62(4), 947-953. doi:https://doi.org/10.1016/0300-9629(79)90033-1

Hertz, P. E., & Huey, R. B. (1981). Compensation for Altitudinal Changes in the Thermal Environment by Some Anolis Lizards on Hispaniola. Ecology, 62(3), 515-521. doi:https://doi.org/10.2307/1937714

Hertz, P. E., Huey, R. B., & Nevo, E. (1983). Homage to Santa Anita: Thermal Sensitivity of Sprint Speed in Agamid Lizards. Evolution, 37(5), 1075-1084. doi:10.2307/2408420

Hertz, P. E., & Nevo, E. (1981). THERMAL BIOLOGY OF FOUR ISRAELI AGAMID LIZARDS IN EARLY SUMMER. Israel Journal of Zoology, 30(4), 190-210. doi:10.1080/00212210.1981.10688514

Heulin, B., Ghielmi, S., Vogrin, N., Surget-Groba, Y., & Guillaume, C. P. (2002). Variation in eggshell characteristics and in intrauterine egg retention between two oviparous clades of the lizard lacerta vivipara: insight into the oviparity–viviparity continuum in squamates. Journal of Morphology, 252(3), 255-262. doi:https://doi.org/10.1002/jmor.1103

Heulin, B., Kirsten, O.-L., & David, M. (1997). Demography of a Bimodal Reproductive Species of Lizard (Lacerta vivipara): Survival and Density Characteristics of Oviparous Populations. Herpetologica, 53(4), 432-444.

Hileman, E. T., King, R. B., Adamski, J. M., Anton, T. G., Bailey, R. L., Baker, S. J., . . . Yagi, A. (2017). Climatic and geographic predictors of life history variation in Eastern Massasauga (Sistrurus catenatus): A range-wide synthesis. Plos One, 12(2), e0172011. doi:10.1371/journal.pone.0172011

Hill, P., While, G. M., Burridge, C. P., Ezaz, T., Munch, K. L., McVarish, M., & Wapstra, E. (2022). Sex reversal explains some, but not all, climate-mediated sex ratio variation within a viviparous reptile. Proceedings of the Royal Society B: Biological Sciences, 289(1976), 20220689. doi:doi:10.1098/rspb.2022.0689

Hodgson, M. J., & Schwanz, L. E. (2019). Drop it like it's hot: Interpopulation variation in thermal phenotypes shows counter-gradient pattern. Journal of Thermal Biology, 83, 178-186. doi:https://doi.org/10.1016/j.jtherbio.2019.05.016

Holden, K. G., Gangloff, E. J., Miller, D. A. W., Hedrick, A. R., Dinsmore, C., Basel, A., . . . Bronikowski, A. M. (2022). Over a decade of field physiology reveals life-history specific strategies to drought in garter snakes Thamnophis elegans. Proceedings of the Royal Society B: Biological Sciences, 289(1967), 20212187. doi:doi:10.1098/rspb.2021.2187

Horváthová, T., Cooney, C. R., Fitze, P. S., Oksanen, T. A., Jelić, D., Ghira, I., . . . Jandzik, D. (2013). Length of activity season drives geographic variation in body size of a widely distributed lizard. Ecology and evolution, 3(8), 2424-2442. doi:https://doi.org/10.1002/ece3.613

Hu, Q., Lin, Y., Qiu, X., Fu, J., & Qi, Y. (2022). High-elevation adaptation of motion visual display modifications in the toad-headed agamid lizards (Phrynocephalus). Asian Herpetological Research, 13(1), 53-63F.

Hu, Y.-C., Lu, H.-L., Cheng, K.-M., Luo, L.-G., & Zeng, Z.-G. (2019). Thermal dependence of feeding performance and resting metabolic expenditure in different altitudinal populations of toad-headed lizards. Journal of Thermal Biology, 80, 16-20. doi:https://doi.org/10.1016/j.jtherbio.2019.01.001

Huey, R. B., & Dunham, A. E. (1987). Repeatability of Locomotor Performance in Natural Populations of the Lizard Sceloporus merriami. Evolution, 41(5), 1116-1120. doi:10.2307/2409195

Huey, R. B., Dunham, A. E., Overall, K. L., & Newman, R. A. (1990). Variation in Locomotor Performance in Demographically Known Populations of the Lizard Sceloporus merriami. Physiological Zoology, 63(5), 845-872. doi:10.1086/physzool.63.5.30152617

Ibargüengoytía, N. R., Acosta, J. C., Boretto, J. M., Villavicencio, H. J., Marinero, J. A., & Krenz, J. D. (2008). Field thermal biology in Phymaturus lizards: Comparisons from the Andes to the Patagonian steppe in Argentina. Journal of Arid Environments, 72(9), 1620-1630. doi:https://doi.org/10.1016/j.jaridenv.2008.03.018

Iraeta, P., Monasterio, C., Salvador, A., & Díaz, J. A. (2006). Mediterranean Hatchling Lizards Grow Faster at Higher Altitude: A Reciprocal Transplant Experiment. Functional Ecology, 20(5), 865-872.

IRAETA, P., MONASTERIO, C., SALVADOR, A., & DÍAZ, J. A. (2011). Sexual dimorphism and interpopulation differences in lizard hind limb length: locomotor performance or chemical signalling? Biological Journal of the Linnean Society, 104(2), 318-329. doi:10.1111/j.1095-8312.2011.01739.x

Iraeta, P., Salvador, A., & Díaz, J. A. (2008). A reciprocal transplant study of activity, body size, and winter survivorship in juvenile lizards from two sites at different altitude. Écoscience, 15(3), 298-304. doi:10.2980/15-3-3119

Iraeta, P., Salvador, A., & Díaz, J. A. (2012). Effects of Caudal Autotomy on Postnatal Growth Rates of Hatchling Psammodromus algirus. Journal of Herpetology, 46(3), 342-345, 344.

Iraeta, P., Salvador, A., & Díaz, J. A. (2013). Life-history traits of two Mediterranean lizard populations: a possible example of countergradient covariation. Oecologia, 172(1), 167-176. doi:10.1007/s00442-012-2492-8

Iraeta, P., Salvador, A., Monasterio, C., & Díaz, J. (2010). Effects of gravidity on the locomotor performance and escape behaviour of two lizard populations: the importance of habitat structure. Behaviour, 147(1), 133-150. doi:https://doi.org/10.1163/000579509X12512773678411

Jameson, E. W., Heusner, A. A., & Lem, D. (1980). Seasonal, sexual and altitudinal variations in stomach content and ingested fat in Sceloporus occidentalis ( Sierra Nevada, California). Journal of Herpetology, 14(3), 255-261. doi:10.2307/1563547

Jaramillo-Alba, J. L., Díaz de la Vega-Pérez, A. H., Bucio-Jiménez, L. E., Méndez-De la Cruz, F. R., & Pérez-Mendoza, H. A. (2020). Comparative thermal ecology parameters of the mexican dusky rattlesnake (Crotalus triseriatus). Journal of Thermal Biology, 92, 102695. doi:https://doi.org/10.1016/j.jtherbio.2020.102695

JI, X., & WANG, Z.-W. (2005). Geographic variation in reproductive traits and trade-offs between size and number of eggs of the Chinese cobra (Naja atra). Biological Journal of the Linnean Society, 85(1), 27-40. doi:10.1111/j.1095-8312.2005.00470.x

Jiang, Z.-W., Ma, L., Mi, C.-R., & Du, W.-G. (2021). Effects of hypoxia on the thermal physiology of a high-elevation lizard: implications for upslope-shifting species. Biology Letters, 17(3), 20200873. doi:doi:10.1098/rsbl.2020.0873

Jin, Y., Liu, N., & Li, J. (2007). Elevational variation in body size of Phrynocephalus vlangalii in the North Qinghai-Xizang (Tibetan) Plateau. Belgian Journal of Zoology, 137(2), 197.

Jin, Y., Y C Brandt, D., Li, J., Wo, Y., Tong, H., & Shchur, V. (2020). Elevation as a selective force on mitochondrial respiratory chain complexes of the Phrynocephalus lizards in the Tibetan plateau. Current Zoology, 67(2), 191-199. doi:10.1093/cz/zoaa056

Jin, Y.-T., & Liu, N.-F. (2007). Altitudinal variation in reproductive strategy of the toad-headed lizard, Phrynocephalus vlangalii in North Tibet Plateau (Qinghai). Amphibia-Reptilia, 28(4), 509-515. doi:https://doi.org/10.1163/156853807782152507

Jovanović Glavaš, O., Kolarić, A., Eross, M., & Jelić, D. (2018). Morphology and reproduction of the Snake-eyed Skink (Ablepharus kitaibelii Bibron & Bory De Saint-Vincent, 1833) in the western most parts of its range. North-Western Journal of Zoology, 14(2).

Kidov, A., Kondratova, T., Ivolga, R., & Lyapkov, S. (2023). Age Structure, Growth, and Reproduction of the Twin-Striped Skink (Ablepharus bivittatus, Reptilia, Scincidae) in the Talysh Mountains (Ardabil Province, Iran). Biology Bulletin, 50(9), 2271-2277.

Kouyoumdjian, L., Gangloff, E. J., Souchet, J., Cordero, G. A., Dupoué, A., & Aubret, F. (2019). Transplanting gravid lizards to high elevation alters maternal and embryonic oxygen physiology, but not reproductive success or hatchling phenotype. Journal of Experimental Biology, 222(14). doi:10.1242/jeb.206839

Kubisch, E., Piantoni, C., Williams, J., Scolaro, A., Navas, C. A., & Ibargüengoytía, N. R. (2012). Do Higher Temperatures Increase Growth in the Nocturnal Gecko Homonota darwini (Gekkota: Phyllodactylidae)? A Skeletochronological Assessment Analyzed at Temporal and Geographic Scales. Journal of Herpetology, 46(4), 587-595, 589.

KURNAZ, M., BÜLBÜL, U., EROğLU, A. İ., UZUN, F., KOÇ, H., & KUTRUP, B. (2018). Age and growth of the Artvin Lizard, Darevskia derjugini (NIKOLSKY, 1898), in Turkey. Herpetozoa, 30, 147-158.

Kylie A. Robert, & Anne M. Bronikowski. (2010). Evolution of Senescence in Nature: Physiological Evolution in Populations of Garter Snake with Divergent Life Histories. The American Naturalist, 175(2), 147-159. doi:10.1086/649595

Lauren B. Buckley. (2008). Linking Traits to Energetics and Population Dynamics to Predict Lizard Ranges in Changing Environments. The American Naturalist, 171(1), E1-E19. doi:10.1086/523949

LeMaster, M. P., & Mason, R. T. (2003). Pheromonally Mediated Sexual Isolation Among Denning Populations of Red-Sided Garter Snakes, Thamnophis sirtalis parietalis. Journal of Chemical Ecology, 29(4), 1027-1043. doi:10.1023/A:1022900304056

Lemos-Espinal, J. A., & Ballinger, R. E. (1995). Comparative thermal ecology of the high-altitude lizard Sceloporus grammicus on the eastern slope of the Iztaccihuatl Volcano, Puebla, Mexico. Canadian Journal of Zoology, 73(12), 2184-2191. doi:10.1139/z95-258

Lemos-Espinal, J. A., & Ballinger, R. E. (1995). Ecology of growth of the high altitude lizard Sceloporus grammicus on the eastern slope of Iztaccihuatl volcano, Puebla, México. Transactions of the Nebraska Academy of Sciences and Affiliated Societies, 22, 77-85.

Lemos-Espinal, J. A., Ballinger, R. E., & Smith, G. R. (1998). COMPARATIVE DEMOGRAPHY OF THE HIGH-ALTITUDE LIZARD, SCELOPORUS GRAMMICUS (PHRYNOSOMATIDAE), ON THE IZTACCIHUATL VOLCANO, PUEBLA, MÉXICO. The Great Basin Naturalist, 58(4), 375-379.

Li, H., Qu, Y.-F., Ding, G.-H., & Ji, X. (2011). Life-History Variation with Respect to Experienced Thermal Environments in the Lizard, Eremias multiocellata (Lacertidae). Zoological Science, 28(5), 332-338, 337.

Li, J., Zhou, R., & Liu, N. (2014). Life-history variation among three populations of the toad-headed lizard Phrynocephalus vlangalii along an elevation gradient on the northeastern Tibetan Plateau. The Herpetological Journal, 24(1), 17-23.

LI, X., WU, P., MA, L., HUEBNER, C., SUN, B., & LI, S. (2020). Embryonic and post-embryonic responses to high-elevation hypoxia in a low-elevation lizard. Integrative Zoology, 15(4), 338-348. doi:https://doi.org/10.1111/1749-4877.12441

Liang, T., Zhou, L., He, W., Xiao, L., & Shi, L. (2018). Variations in the reproductive strategies of three populations of Phrynocephalus helioscopus in China. PeerJ, 6, e5705. doi:10.7717/peerj.5705

Llanos-Garrido, A., Díaz, J. A., Pérez-Rodríguez, A., & Arriero, E. (2017). Variation in male ornaments in two lizard populations with contrasting parasite loads. Journal of Zoology, 303(3), 218-225. doi:https://doi.org/10.1111/jzo.12478

Llewelyn, J., Macdonald, S. L., Hatcher, A., Moritz, C., & Phillips, B. L. (2016). Intraspecific variation in climate-relevant traits in a tropical rainforest lizard. Diversity and Distributions, 22(10), 1000-1012. doi:https://doi.org/10.1111/ddi.12466

LLEWELYN, J., MACDONALD, S. L., MORITZ, C., MARTINS, F., HATCHER, A., & PHILLIPS, B. L. (2018). Adjusting to climate: Acclimation, adaptation and developmental plasticity in physiological traits of a tropical rainforest lizard. Integrative Zoology, 13(4), 411-427. doi:https://doi.org/10.1111/1749-4877.12309

Lu, H.-L., Lin, Z.-H., Li, H., & Ji, X. (2014). Geographic variation in hatchling size in an oviparous skink: effects of maternal investment and incubation thermal environment. Biological Journal of the Linnean Society, 113(1), 283-296. doi:10.1111/bij.12322

Lu, H.-L., Xu, C.-X., Jin, Y.-T., Hero, J.-M., & Du, W.-G. (2018). Proximate causes of altitudinal differences in body size in an agamid lizard. Ecology and evolution, 8(1), 645-654. doi:https://doi.org/10.1002/ece3.3686

Lu, H.-L., Xu, C.-X., Zeng, Z.-G., & Du, W.-G. (2018). Environmental causes of between-population difference in growth rate of a high-altitude lizard. BMC Ecology, 18(1), 37. doi:10.1186/s12898-018-0194-8

Luiselli, L., Filippi, E., & Capula, M. (2005). Geographic variation in diet composition of the grass snake (Natrix natrix) along the mainland and an island of Italy: the effects of habitat type and interference with potential competitors. The Herpetological Journal, 15(4), 221-230.

Luiselli, L., & Zimmermann, P. (1997). Thermal ecology and reproductive cyclicity of the snake Natrix tessellata in south-eastern Austria and central Italy: a comparative study. Amphibia-Reptilia, 18(4), 383-396. doi:https://doi.org/10.1163/156853897X00431

Luiselli, L., & Zuffi, M. A. (2002). Female life-history traits of the aspic viper (Vipera aspis) and sand viper (V. ammodytes) from the Mediterranean region. Biology of the vipers. Eagle Mountain Publication, Eagle Mountain, Utah, 279-284.

Ma, L., Liu, P., Su, S., Luo, L.-G., Zhao, W.-G., & Ji, X. (2019). Life-history consequences of local adaptation in lizards: Takydromus wolteri (Lacertidae) as a model organism. Biological Journal of the Linnean Society, 127(1), 88-99. doi:10.1093/biolinnean/blz024

Marion, K. R., & Sexton, O. J. (1971). The Reproductive Cycle of the Lizard Sceloporus malachiticus in Costa Rica. Copeia, 1971(3), 517-526. doi:10.2307/1442449

Martín, J., Javier Zamora-Camacho, F., Reguera, S., López, P., & Moreno-Rueda, G. (2017). Variations in chemical sexual signals of Psammodromus algirus lizards along an elevation gradient may reflect altitudinal variation in microclimatic conditions. The Science of Nature, 104(3), 16. doi:10.1007/s00114-017-1442-z

Martin, W. H. (2002). Life history constraints on the timber rattlesnake (Crotalus horridus) at its climatic limits. Biology of the Vipers, 285-306.

Mathies, T., & Andrews, R. M. (1995). Thermal and reproductive biology of high and low elevation populations of the lizard Sceloporus scalaris: implications for the evolution of viviparity. Oecologia, 104(1), 101-111. doi:10.1007/BF00365568

McMillan, D. M., Irschick, D. J., & Rees, B. B. (2011). Geographic Variation in the Effects of Heat Exposure on Maximum Sprint Speed and Hsp70 Expression in the Western Fence Lizard Sceloporus occidentalis. Physiological and Biochemical Zoology, 84(6), 573-582. doi:10.1086/662385

Medel, R. G. (1992). Costs and benefits of tail loss: assessing economy of autotomy in two lizard species of central Chile. Revista chilena de historia natural, 65, 357-361.

Medina, M., Gutierrez, J., Scolaro, A., & Ibargüengoytía, N. (2009). Thermal responses to environmental constraints in two populations of the oviparous lizard Liolaemus bibronii in Patagonia, Argentina. Journal of Thermal Biology, 34(1), 32-40. doi:https://doi.org/10.1016/j.jtherbio.2008.10.001

Medina, M., & Ibargüengoytía, N. R. (2010). How do viviparous and oviparous lizards reproduce in Patagonia? A comparative study of three species of Liolaemus. Journal of Arid Environments, 74(9), 1024-1032. doi:https://doi.org/10.1016/j.jaridenv.2010.02.004

Medina, M., Scolaro, A., Méndez-De la Cruz, F., Sinervo, B., & Ibargüengoytía, N. (2011). Thermal relationships between body temperature and environment conditions set upper distributional limits on oviparous species. Journal of Thermal Biology, 36(8), 527-534. doi:https://doi.org/10.1016/j.jtherbio.2011.09.005

Meister, B., Ursenbacher, S., & Baur, B. (2012). Grass Snake Population Differentiation over Different Geographic Scales. Herpetologica, 68(1), 134-145. doi:10.1655/herpetologica-d-11-00036.1

Mendez-de la Cruz, F. R., Guillette, L. J., & Cruz, M. V.-S. (1993). Differential Atresia of Ovarian Follicles and its Effect on the Clutch Size of Two Populations of the Viviparous Lizard Sceloporus mucronatus. Functional Ecology, 7(5), 535-540. doi:10.2307/2390129

Méndez-Galeano, M. A., Paternina-Cruz, R. F., & Calderón-Espinosa, M. L. (2020). The highest kingdom of Anolis: Thermal biology of the Andean lizard Anolis heterodermus (Squamata: Dactyloidae) over an elevational gradient in the Eastern Cordillera of Colombia. Journal of Thermal Biology, 89, 102498. doi:https://doi.org/10.1016/j.jtherbio.2019.102498

Meshaka, W. J. E., & Delis, P. R. (2010). Clutch sizes in two populations of the Eastern Garter Snake (Thamnophis sirtalis) in Pennsylvania. Reptiles & Amphibians, 17(4), 206-220.

Miller, D. A., Clark, W. R., Arnold, S. J., & Bronikowski, A. M. (2011). Stochastic population dynamics in populations of western terrestrial garter snakes with divergent life histories. Ecology, 92(8), 1658-1671. doi:https://doi.org/10.1890/10-1438.1

Miranda-Calle, A. B., Pacheco, L. F., Aparicio, J., & Méndez-De la Cruz, F. R. (2021). Thermoregulation of Liolaemus aparicioi (Iguania: Liolaemidae) along a 1000 m elevational gradient in La Paz Valley, La Paz, Bolivia. Journal of Thermal Biology, 99, 102940. doi:https://doi.org/10.1016/j.jtherbio.2021.102940

Molina-Borja, M. (2003). Sexual Dimorphism of Gallotia atlantica atlantica and Gallotia atlantica mahoratae (Lacertidae) from the Eastern Canary Islands. Journal of Herpetology, 37(4), 769-772, 764.

Monasterio, C., Salvador, A., Iraeta, P., & Díaz, J. A. (2009). The effects of thermal biology and refuge availability on the restricted distribution of an alpine lizard. Journal of Biogeography, 36(9), 1673-1684. doi:https://doi.org/10.1111/j.1365-2699.2009.02113.x

Monasterio, C., Verdú-Ricoy, J., Salvador, A., & Díaz, J. A. (2016). Living at the edge: lower success of eggs and hatchlings at lower elevation may shape range limits in an alpine lizard. Biological Journal of the Linnean Society, 118(4), 829-841. doi:10.1111/bij.12766

Monney, J.-C., Luiselli, L., & Capula, M. (1995). Correlates of melanism in a population of adders (Vipera berus) from the Swiss Alps and comparisons with other alpine populations. Amphibia-Reptilia, 16(4), 323-330. doi:https://doi.org/10.1163/156853895X00406

Moreno-Rueda, G., González-Granda, L. G., Reguera, S., Zamora-Camacho, F. J., & Melero, E. (2019). Crypsis Decreases with Elevation in a Lizard. Diversity, 11(12), 236.

Moreno-Rueda, G., Melero, E., Reguera, S., Zamora-Camacho, F. J., & Álvarez-Benito, I. (2017). Prey availability, prey selection, and trophic niche width in the lizard Psammodromus algirus along an elevational gradient. Current Zoology, 64(5), 603-613. doi:10.1093/cz/zox077

Moreno-Rueda, G., Reguera, S., Zamora-Camacho, F. J., & Comas, M. (2021). Inter-Individual Differences in Ornamental Colouration in a Mediterranean Lizard in Relation to Altitude, Season, Sex, Age, and Body Traits. Diversity, 13(4), 158.

Muñoz, M. M., & Losos, J. B. (2018). Thermoregulatory Behavior Simultaneously Promotes and Forestalls Evolution in a Tropical Lizard. The American Naturalist, 191(1), E15-E26. doi:10.1086/694779

Muñoz, M. M., Stimola, M. A., Algar, A. C., Conover, A., Rodriguez, A. J., Landestoy, M. A., . . . Losos, J. B. (2014). Evolutionary stasis and lability in thermal physiology in a group of tropical lizards. Proceedings of the Royal Society B: Biological Sciences, 281(1778), 20132433. doi:doi:10.1098/rspb.2013.2433

Niewiarowski, P. H. (2001). Energy Budgets, Growth Rates, and Thermal Constraints: Toward an Integrative Approach to the Study of Life‐History Variation. The American Naturalist, 157(4), 421-433. doi:10.1086/319321

Niewiarowski, P. H., & Roosenburg, W. (1993). Reciprocal Transplant Reveals Sources of Variation in Growth Rates of the Lizard Sceloporus Undulatus. Ecology, 74(7), 1992-2002. doi:10.2307/1940842

Nogueira, C., Sawaya, R. J., & Martins, M. (2003). Ecology of the Pitviper, Bothrops moojeni, in the Brazilian Cerrado. Journal of Herpetology, 37(4), 653-659, 657.

Orlova, V., Kuranova, V., & Bulakhova, N. (2003). Some aspects of reproductive biology of Zootoca vivipara (Jacquin, 1787) in the Asian part of its area. HERPETOLOGIA PETROPOLITANA, 201.

Ortega, J., López, P., & Martín, J. (2015). Altitudinally divergent adult phenotypes in Iberian wall lizards are not driven by egg differences or hatchling growth rates. Oecologia, 177(2), 357-366. doi:10.1007/s00442-014-3185-2

Ortega, J., López, P., & Martín, J. (2017). Environmental drivers of growth rates in Guadarrama wall lizards: a reciprocal transplant experiment. Biological Journal of the Linnean Society, 122(2), 340-350. doi:10.1093/biolinnean/blx068

Ortega, J., Martín, J., Crochét, P. A., Löpez, P., & Clobert, J. (2019). Seasonal and interpopulational phenotypic variation in morphology and sexual signals of Podarcis liolepis lizards. Plos One, 14(3). doi:10.1371/journal.pone.0211686

Overall, K. L., Vitt, L., & Pianka, E. (1994). Lizard egg environments. Lizard ecology: historical and experimental perspectives, 51-72.

Palacios, M. G., & Bronikowski, A. M. (2017). Immune variation during pregnancy suggests immune component-specific costs of reproduction in a viviparous snake with disparate life-history strategies. Journal of Experimental Zoology Part A: Ecological and Integrative Physiology, 327(8), 513-522. doi:https://doi.org/10.1002/jez.2137

Palacios, M. G., Cunnick, J. E., & Bronikowski, A. M. (2013). Complex Interplay of Body Condition, Life History, and Prevailing Environment Shapes Immune Defenses of Garter Snakes in the Wild. Physiological and Biochemical Zoology, 86(5), 547-558. doi:10.1086/672371

Palacios, M. G., Sparkman, A. M., & Bronikowski, A. M. (2011). Developmental plasticity of immune defence in two life-history ecotypes of the garter snake, Thamnophis elegans – a common-environment experiment. Journal of Animal Ecology, 80(2), 431-437. doi:https://doi.org/10.1111/j.1365-2656.2010.01785.x

Palacios, M. G., Sparkman, A. M., & Bronikowski, A. M. (2012). Corticosterone and pace of life in two life-history ecotypes of the garter snake Thamnophis elegans. General and Comparative Endocrinology, 175(3), 443-448. doi:https://doi.org/10.1016/j.ygcen.2011.11.042

Patterson, J. W. (1990). Female reproductive cycles in two subspecies of the tropical lizard Mabuya striata. Oecologia, 84(2), 232-237. doi:10.1007/BF00318277

PATTERSON, J. W. (1991). Emergence, basking behaviour, mean selected temperature and critical thermal minimum in high and low altitude subspecies of the tropical lizard Mabuya striata. African Journal of Ecology, 29(4), 330-339. doi:https://doi.org/10.1111/j.1365-2028.1991.tb00470.x

Perera, A., Pérez-Mellado, V., Carretero, M. A., & Harris, D. J. (2006). Variation between populations in the diet of the mediterranean lizard Lacerta Perspicillata. Herpetological Journal, 16(2), 107-113.

Pérez-Mendoza, H. A., Sanabria-Tobón, S. R., Jaramillo-Alba, J. L., Solano-Zavaleta, I., Vázquez-Vega, L. F., & de la Vega-Pérez, A. H. D. (2018). Reproductive Traits of Dusky Rattlesnakes (Crotalus triseriatus) in Central Mexico. Journal of Herpetology, 52(1), 6-11. doi:10.1670/16-003

Pérez-Mendoza, H. A., & Zúñiga-Vega, J. J. (2014). A test of the fast–slow continuum model of life-history variation in the lizard Sceloporus grammicus. Evolutionary Ecology Research, 16(3), 235-248.

Pérez-Mendoza, H. A., Zúñiga-Vega, J. J., Martorell, C., Zurita-Gutiérrez, Y. H., Solano-Zavaleta, I., Hernández-Rosas, A. L., & Molina-Moctezuma, A. (2014). Patterns of spatio-temporal variation in the survival rates of a viviparous lizard: the interacting effects of sex, reproductive trade-offs, aridity, and human-induced disturbance. Population Ecology, 56(4), 605-618. doi:10.1007/s10144-014-0447-0

Perold, V., Ferguson, J. W. H., Verburgt, L., & Malherbe, J. B. (2021). Are high elevation crag lizards sensitive to climate change? Austral Ecology, 46(3), 359-373. doi:https://doi.org/10.1111/aec.12991

Perry, C., Sarraude, T., Billet, M., Minot, E., Gangloff, E. J., & Aubret, F. (2024). Sex-dependent shifts in body size and condition along replicated elevational gradients in a montane colonising ectotherm, the common wall lizard (Podarcis muralis). Oecologia, 206(3-4), 335-346. doi:10.1007/s00442-024-05634-8

Pettersen, A. K., Ruuskanen, S., Nord, A., Nilsson, J. F., Miñano, M. R., Fitzpatrick, L. J., . . . Uller, T. (2023). Population divergence in maternal investment and embryo energy use and allocation suggests adaptive responses to cool climates. Journal of Animal Ecology, 92(9), 1771-1785. doi:10.1111/1365-2656.13971

Pinzón-Barrera, C., Suárez-Ayala, N., Carrillo-Chávez, L. M., Camacho-González, C. A., Calderón-Espinosa, M. L., & Pinto-Sánchez, N. R. (2024). Unveiling Critical Thermal Limits of Anolis tolimensis (Squamata, Anolidae) across an Elevational Landscape. Current Herpetology, 43(1), 115-134. doi:10.5358/hsj.43.115

Plasman, M., Bautista, A., & Díaz de la Vega-Pérez, A. H. (2022). Avoiding the effects of translocation on the estimates of the metabolic rates across an elevational gradient. Journal of Comparative Physiology B, 192(5), 659-668. doi:10.1007/s00360-022-01448-3

PLASMAN, M., BAUTISTA, A., McCUE, M. D., & DÍAZ DE LA VEGA-PÉREZ, A. H. (2020). Resting metabolic rates increase with elevation in a mountain-dwelling lizard. Integrative Zoology, 15(5), 363-374. doi:https://doi.org/10.1111/1749-4877.12434

Prival, D. B., & Schroff, M. J. (2012). A 13-year study of a northern population of twin-spotted rattlesnakes (Crotalus pricei): growth, reproduction, survival, and conservation. Herpetological Monographs, 26(1), 1-18.

Punzo, F. (2007). Life history, demography, diet and habitat associations in the southwestern earless lizard, Cophosaurus texanus scitulus from northern and southern limits of its geographical range. Amphibia-Reptilia, 28(1), 65-76. doi:https://doi.org/10.1163/156853807779798974

QU, Y.-F., LI, H., GAO, J.-F., & JI, X. (2011). Geographical variation in reproductive traits and trade-offs between size and number of eggs in the king ratsnake, Elaphe carinata. Biological Journal of the Linnean Society, 104(3), 701-709. doi:10.1111/j.1095-8312.2011.01749.x

Qualls, C. P., & Andrews, R. M. (1999). Maternal body volume constrains water uptake by lizard eggs in utero. Functional Ecology, 13(6), 845-851. doi:https://doi.org/10.1046/j.1365-2435.1999.00374.x

QUALLS, C. P., & ANDREWS, R. M. (2008). Cold climates and the evolution of viviparity in reptiles: cold incubation temperatures produce poor-quality offspring in the lizard, Sceloporus virgatus. Biological Journal of the Linnean Society, 67(3), 353-376. doi:10.1111/j.1095-8312.1999.tb01939.x

QUALLS, F. J., & SHINE, R. (1997). Geographic variation in ‘costs of reproduction’ in the scincid lizard Lampropholis guichenoti. Functional Ecology, 11(6), 757-763. doi:https://doi.org/10.1046/j.1365-2435.1997.00150.x

Qualls, F. J., & Shine, R. (1998). Geographic variation in lizard phenotypes: importance of the incubation environment. Biological Journal of the Linnean Society, 64(4), 477-491. doi:10.1111/j.1095-8312.1998.tb00345.x

Radder, R. S. (2006). An overview of geographic variation in the life history traits of the tropical agamid lizard, Calotes versicolor. Current Science, 91(10), 1354-1363.

Ramírez-Bautista, A., Cruz-Elizalde, R., Hernández-Salinas, U., Lozano, A., & Grummer, J. A. (2017). Reproductive trait variation in the Sceloporus scalaris species group (Squamata: Phrynosomatidae) from the Transvolcanic Belt, Mexico. Biological Journal of the Linnean Society, 122(4), 838-849. doi:10.1093/biolinnean/blx100

Ramírez-Bautista, A., Cruz-Elizalde, R., Stephenson, B. P., & Hernández-Salinas, U. (2022). Geographic variation in female body size and clutch size of the lizard Sceloporus variabilis (Squamata: Phrynosomatidae) in Mexico. Acta Zoologica, 103(3), 307-315. doi:https://doi.org/10.1111/azo.12372

Ramírez-Bautista, A., Jiménez-Cruz, E., & Marshall, J. C. (2004). COMPARATIVE LIFE HISTORY FOR POPULATIONS OF THE SCELOPORUS GRAMMICUS COMPLEX (SQUAMATA: PHRYNOSOMATIDAE). Western North American Naturalist, 64(2), 175-183.

Ramírez-Bautista, A., Leyte-Manrique, A., Marshall, J. C., & Smith, G. R. (2011). Effects of Elevation on Litter-Size Variation Among Lizard Populations in the Sceloporus grammicus Complex (Phrynosomatidae) in Mexico. Western North American Naturalist, 71(2), 215-221, 217.

Ramirez-Bautista, A., & Pavon, N. P. (2009). Sexual dimorphism and reproductive cycle in the arboreal spiny lizard Sceloporus formosus Wiegmann (Squamata: Phrynosomatidae) from central Oaxaca, Mexico. Revista chilena de historia natural, 82(4), 553-563.

Ramírez-Bautista, A., Stephenson, B. P., Serrano Muñoz, C., Cruz-Elizalde, R., & Hernández-Salinas, U. (2014). Reproduction and sexual dimorphism in two populations of the polymorphic spiny lizard Sceloporus minor from Hidalgo, México. Acta Zoologica, 95(4), 397-408. doi:https://doi.org/10.1111/azo.12037

Refsnider, J. M., Qian, S. S., Streby, H. M., Carter, S. E., Clifton, I. T., Siefker, A. D., & Vazquez, T. K. (2018). Reciprocally transplanted lizards along an elevational gradient match light environment use of local lizards via phenotypic plasticity. Functional Ecology, 32(5), 1227-1236. doi:https://doi.org/10.1111/1365-2435.13071

Refsnider, J. M., Vazquez, T. K., Clifton, I. T., Jayawardena, D. M., & Heckathorn, S. A. (2021). Cellular and whole-organism effects of prolonged versus acute heat stress in a montane, desert lizard. Journal of Experimental Zoology Part A: Ecological and Integrative Physiology, 335(1), 126-135. doi:https://doi.org/10.1002/jez.2426

Reguera, S., Zamora-Camacho, F. J., Melero, E., García-Mesa, S., Trenzado, C. E., Cabrerizo, M. J., . . . Moreno-Rueda, G. (2015). Ultraviolet radiation does not increase oxidative stress in the lizard Psammodromus algirus along an elevational gradient. Comparative Biochemistry and Physiology Part A: Molecular & Integrative Physiology, 183, 20-26. doi:https://doi.org/10.1016/j.cbpa.2014.12.015

Reguera, S., Zamora-Camacho, F. J., & Moreno-Rueda, G. (2014). The lizard Psammodromus algirus (Squamata: Lacertidae) is darker at high altitudes. Biological Journal of the Linnean Society, 112(1), 132-141. doi:10.1111/bij.12250

Reguera, S., Zamora-Camacho, F. J., Trenzado, C. E., Sanz, A., & Moreno-Rueda, G. (2014). Oxidative stress decreases with elevation in the lizard Psammodromus algirus. Comparative Biochemistry and Physiology Part A: Molecular & Integrative Physiology, 172, 52-56. doi:https://doi.org/10.1016/j.cbpa.2014.02.018

Rivera-Rea, J., Macotela, L., Moreno-Rueda, G., Suárez-Varón, G., Bastiaans, E., Quintana, E., & González-Morales, J. C. (2023). Thermoregulatory behavior varies with altitude and season in the sceloporine mesquite lizard. Journal of Thermal Biology, 114, 103539. doi:https://doi.org/10.1016/j.jtherbio.2023.103539

Rock, J., Andrews, R. M., & Cree, A. (2000). Effects of Reproductive Condition, Season, and Site on Selected Temperatures of a Viviparous Gecko. Physiological and Biochemical Zoology, 73(3), 344-355. doi:10.1086/316741

Rock, J., & Cree, A. (2003). INTRASPECIFIC VARIATION IN THE EFFECT OF TEMPERATURE ON PREGNANCY IN THE VIVIPAROUS GECKO HOPLODACTYLUS MACULATUS. Herpetologica, 59(1), 8-22. doi:10.1655/0018-0831(2003)059[0008:Iviteo]2.0.Co;2

Rodríguez‐Díaz, T., & Braña, F. (2012). Altitudinal variation in egg retention and rates of embryonic development in oviparous Zootoca vivipara fits predictions from the cold‐climate model on the evolution of viviparity. Journal of Evolutionary Biology, 25(9), 1877-1887. doi:10.1111/j.1420-9101.2012.02575.x

Rohr, D. H. (1997). Demographic and Life-History Variation in Two Proximate Populations of a Viviparous Skink Separated by a Steep Altitudinal Gradient. Journal of Animal Ecology, 66(4), 567-578. doi:10.2307/5950

Roitberg, E. S., & Smirina, E. M. (2006). Age, body size and growth of Lacerta agilis boemica and L. strigata: a comparative study of two closely related lizard species based on skeletochronology. The Herpetological Journal, 16(2), 133-148.

Roitberg, E. S., Smirina, E. M., & Koltsov, N. (2006). Adult body length and sexual size dimorphism in Lacerta agilis boemica (Reptilia, Lacertidae): between-year and interlocality variation. Mainland and insular lacertid lizards: A mediterranean perspective, 175-187.

Rojas-González, I., Jones, C., Zúñiga-Vega, J., & Lemos-Espinal, J. (2008). Demography of Xenosaurus platyceps (Squamata: Xenosauridae): a comparison between tropical and temperate populations. Amphibia-Reptilia, 29(2), 245-256. doi:https://doi.org/10.1163/156853808784124992

Rojas-González, R. I., Lemos-Espinal, J. A., & Smith, G. R. (2022). Individual growth of the Flathead Knob-scaled Lizard, Xenosaurus platyceps, from tropical and temperate populations. Biotropica, 54(5), 1217-1225. doi:https://doi.org/10.1111/btp.13144

Rojas-González, R. I., Zúñiga-Vega, J. J., & Lemos-Espinal, J. A. (2008). Reproductive Variation of the Lizard Xenosaurus platyceps: Comparing Two Populations of Contrasting Environments. Journal of Herpetology, 42(2), 332-336, 335.

Ruby, D. E., & Baird, D. I. (1994). Intraspecific Variation in Behavior: Comparisons between Populations at Different Altitudes of the Lizard Sceloporus jarrovi. Journal of Herpetology, 28(1), 70-78. doi:10.2307/1564683

Ruby, D. E., & Dunham, A. E. (1987). Variation in home range size along an elevational gradient in the iguanid lizard Sceloporus merriami. Oecologia, 71(3), 473-480. doi:10.1007/BF00378723

Rugiero, L., Capula, M., Persichetti, D., Luiselli, L., & Angelici, F. (2000). Life-history and diet of two populations of Natrix maura (Reptilia, Colubridae) from contrasted habitats in Sardinia. Miscel· lània Zoològica, 41-51.

Rutschmann, A., Rozen-Rechels, D., Dupoué, A., Blaimont, P., de Villemereuil, P., Miles, D. B., . . . Clobert, J. (2020). Climate dependent heating efficiency in the common lizard. Ecology and evolution, 10(15), 8007-8017. doi:https://doi.org/10.1002/ece3.6241

Sannolo, M., Civantos, E., Martín, J., & Carretero, M. A. (2020). Variation in field body temperature and total evaporative water loss along an environmental gradient in a diurnal ectotherm. Journal of Zoology, 310(3), 221-231. doi:https://doi.org/10.1111/jzo.12744

Scali, S. (2011). Ecological comparison of the dice snake (Natrix tessellata) and the viperine snake (Natrix maura) in northern Italy. Mertensiella, 18, 131-144.

Schwarzkopf, L., Caley, M. J., & Kearney, M. R. (2016). One lump or two? Explaining a major latitudinal transition in reproductive allocation in a viviparous lizard. Functional Ecology, 30(8), 1373-1383.

Sears, M. W. (2005). Geographic variation in the life history of the sagebrush lizard: the role of thermal constraints on activity. Oecologia, 143(1), 25-36. doi:10.1007/s00442-004-1767-0

Sears, M. W. (2005). Resting metabolic expenditure as a potential source of variation in growth rates of the sagebrush lizard. Comparative Biochemistry and Physiology Part A: Molecular & Integrative Physiology, 140(2), 171-177. doi:https://doi.org/10.1016/j.cbpb.2004.12.003

Sears, M. W., & Angilletta Jr., M. J. (2003). Life-history variation in the sagebrush lizard: Phenotypic plasticity or local adaptation? Ecology, 84(6), 1624-1634. doi:https://doi.org/10.1890/0012-9658(2003)084[1624:LVITSL]2.0.CO;2

Seddon, R. J., & Hews, D. K. (2016). Phenotypic correlates of melanization in two Sceloporus occidentalis (Phrynosomatidae) populations: Behavior, androgens, stress reactivity, and ectoparasites. Physiology & Behavior, 163, 70-80. doi:https://doi.org/10.1016/j.physbeh.2016.04.039

Seddon, R. J., & Hews, D. K. (2016). Populations of the Lizard, Sceloporus occidentalis, that Differ in Melanization have Different Rates of Wound Healing. Journal of Experimental Zoology Part A: Ecological Genetics and Physiology, 325(8), 491-500. doi:https://doi.org/10.1002/jez.2033

Seddon, R. J., & Hews, D. K. (2017). Correlates of melanization in multiple high- and low-elevation populations of the lizard, Sceloporus occidentalis: Behavior, hormones, and parasites. Journal of Experimental Zoology Part A: Ecological and Integrative Physiology, 327(8), 481-492. doi:https://doi.org/10.1002/jez.2133

Serén, N., Megía-Palma, R., Simčič, T., Krofel, M., Guarino, F. M., Pinho, C., . . . Carretero, M. A. (2023). Functional responses in a lizard along a 3.5-km altitudinal gradient. Journal of Biogeography, 50(12), 2042-2056. doi:https://doi.org/10.1111/jbi.14711

Shine, R. (1979). Activity Patterns in Australian Elapid Snakes (Squamata: Serpentes: Elapidae). Herpetologica, 35(1), 1-11.

Shine, R. (1987). Intraspecific Variation in Thermoregulation, Movements and Habitat Use by Australian Blacksnakes, Pseudechis porphyriacus (Elapidae). Journal of Herpetology, 21(3), 165-177. doi:10.2307/1564479

Shine, R. (1999). Egg‐laying reptiles in cold climates: determinants and consequences of nest temperatures in montane lizards. Journal of Evolutionary Biology, 12(5), 918-926. doi:10.1046/j.1420-9101.1999.00093.x

Shine, R. (2002). An empirical test of the `predictability' hypothesis for the evolution of viviparity in reptiles. Journal of Evolutionary Biology, 15(4), 553-560. doi:10.1046/j.1420-9101.2002.00420.x

SHINE, R., ELPHICK, M. J., & BARROTT, E. G. (2003). Sunny side up: lethally high, not low, nest temperatures may prevent oviparous reptiles from reproducing at high elevations. Biological Journal of the Linnean Society, 78(3), 325-334. doi:10.1046/j.1095-8312.2003.00140.x

Sinervo, B. (1990). The evolution of maternal investment in lizards: An experimental and comparative analysis of egg size and its effects on offspring performance. Evolution, 44(2), 279-294. doi:10.1111/j.1558-5646.1990.tb05198.x

Sinervo, B., & Adolph, S. C. (1994). Growth Plasticity and Thermal Opportunity in Sceloporus Lizards. Ecology, 75(3), 776-790. doi:https://doi.org/10.2307/1941734

Sinervo, B., Hedges, R., & Adolph, S. C. (1991). Decreased Sprint Speed as A Cost of Reproduction in the Lizard Sceloporus cccidentals: Variation Among Populations. Journal of Experimental Biology, 155(1), 323-336. doi:10.1242/jeb.155.1.323

Sinervo, B., & Losos, J. B. (1991). Walking the Tight Rope: Arboreal Sprint Performance Among Sceloporus Occidentalis Lizard Populations. Ecology, 72(4), 1225-1233. doi:https://doi.org/10.2307/1941096

Smith, G. R., & Ballinger, R. E. (1994). Temperature Relationships in the High-Altitude Viviparous Lizard, Sceloporus jarrovi. The American Midland Naturalist, 131(1), 181-189. doi:10.2307/2426621

Smith, G. R., & Ballinger, R. E. (1994). Temporal and Spatial Variation in Individual Growth in the Spiny Lizard, Sceloporus jarrovi. Copeia, 1994(4), 1007-1013. doi:10.2307/1446724

Smith, G. R., & Ballinger, R. E. (1994). Thermal tolerance in the tree lizard (Urosaurus ornatus) from a desert population and a low montane population. Canadian Journal of Zoology, 72(11), 2066-2069. doi:10.1139/z94-276

Smith, G. R., & Ballinger, R. E. (1995). Temperature Relationships of the Tree Lizard, Urosaurus ornatus, from Desert and Low-Elevation Montane Populations in the Southwestern USA. Journal of Herpetology, 29(1), 126-129. doi:10.2307/1565097

Smith, G. R., Ballinger, R. E., & Nietfeldt, J. W. (1994). Elevational Variation of Growth Rates in Neonate Sceloporus jarrovi: An Experimental Evaluation. Functional Ecology, 8(2), 215-218. doi:10.2307/2389904

Smith, G. R., Lemos-Espinal, J. A., & Ballinger, R. E. (2003). Body size, sexual dimorphism, and clutch size in two populations of the lizard Sceloporus ochoteranae. The Southwestern Naturalist, 48(1), 123-126.

Smith, S., & Shine, R. (1997). Intraspecific variation in reproductive mode within the scincid lizard Saiphos equalis. Australian Journal of Zoology, 45(5), 435-445.

Sorci, G., Clobert, J., & Belichon, S. (1996). Phenotypic Plasticity of Growth and Survival in the Common Lizard Lacerta vivipara. Journal of Animal Ecology, 65(6), 781-790. doi:10.2307/5676

SOUCHET, J., GANGLOFF, E. J., MICHELI, G., BOSSU, C., TROCHET, A., BERTRAND, R., . . . AUBRET, F. (2020). High-elevation hypoxia impacts perinatal physiology and performance in a potential montane colonizer. Integrative Zoology, 15(6), 544-557. doi:https://doi.org/10.1111/1749-4877.12468

Souchet, J., Josserand, A., Darnet, E., Le Chevalier, H., Trochet, A., Bertrand, R., . . . Gangloff, E. J. (2023). Embryonic and juvenile snakes (Natrix maura, Linnaeus 1758) compensate for high elevation hypoxia via shifts in cardiovascular physiology and metabolism. Journal of Experimental Zoology Part A: Ecological and Integrative Physiology, 339(10), 1102-1115. doi:https://doi.org/10.1002/jez.2756

Souri, A. M., & Rastegar-Pouyani, N. (2017). Morphological characters, antioxidant defences and oxidative stress in the lizard Ophisops elegans at different altitudes. Bilharean Biol, 12(2), 97-101.

Sparkman, A. M., & Palacios, M. G. (2009). A test of life-history theories of immune defence in two ecotypes of the garter snake, Thamnophis elegans. Journal of Animal Ecology, 78(6), 1242-1248. doi:https://doi.org/10.1111/j.1365-2656.2009.01587.x

Sparkman, A. M., Vleck, C. M., & Bronikowski, A. M. (2009). Evolutionary ecology of endocrine-mediated life-history variation in the garter snake Thamnophis elegans. Ecology, 90(3), 720-728. doi:https://doi.org/10.1890/08-0850.1

Stewart, J. R. (1979). The Balance between Number and Size of Young in the Live Bearing Lizard Gerrhonotus coeruleus. Herpetologica, 35(4), 342-350.

Strijbosch, H., Helmer, W., & Scholte, P. T. (1989). Distribution and ecology of lizards in the Greek province of Evros. Amphibia-Reptilia, 10(2), 151-174. doi:https://doi.org/10.1163/156853889X00188

Šukalo, G., Đorđević, S., Gvozdenović, S., Simović, A., Anđelković, M., Blagojević, V., & Tomović, L. (2014). Intra-and inter-population variability of food preferences of two Natrix species on the Balkan Peninsula. Herpetological Conservation and Biology, 9(1), 123-136.

Sun, B.-J., Li, S.-R., Xu, X.-F., Zhao, W.-G., Luo, L.-G., Ji, X., & Du, W.-G. (2013). Different mechanisms lead to convergence of reproductive strategies in two lacertid lizards (Takydromus wolteri and Eremias argus). Oecologia, 172(3), 645-652. doi:10.1007/s00442-012-2524-4

Tanaka, K., & Mori, A. (2010). Reproductive characteristics of Elaphe quadrivirgata (Serpentes: Colubridae) from ecologically dissimilar main island and island populations. Journal of Natural History, 45(3-4), 211-226. doi:10.1080/00222933.2010.522262

Telemeco, R. S. (2014). Immobile and Mobile Life-History Stages Have Different Thermal Physiologies in a Lizard. Physiological and Biochemical Zoology, 87(2), 203-215. doi:10.1086/674959

TELEMECO, R. S., RADDER, R. S., BAIRD, T. A., & SHINE, R. (2010). Thermal effects on reptile reproduction: adaptation and phenotypic plasticity in a montane lizard. Biological Journal of the Linnean Society, 100(3), 642-655. doi:10.1111/j.1095-8312.2010.01439.x

Tinkle, D. W., & Ballinger, R. E. (1972). Sceloporus undulatus: A Study of the Intraspecific Comparative Demography of a Lizard. Ecology, 53(4), 570-584. doi:https://doi.org/10.2307/1934772

Tracy, C. R. (1999). DIFFERENCES IN BODY SIZE AMONG CHUCKWALLA (SAUROMALUS OBESUS) POPULATIONS. Ecology, 80(1), 259-271. doi:https://doi.org/10.1890/0012-9658(1999)080[0259:DIBSAC]2.0.CO;2

Tracy, Christopher R., & Diamond, J. (2005). Regulation of Gut Function Varies with Life‐History Traits in Chuckwallas (Sauromalus obesus: Iguanidae). Physiological and Biochemical Zoology, 78(4), 469-481. doi:10.1086/430232

Trochet, A., Dupoué, A., Souchet, J., Bertrand, R., Deluen, M., Murarasu, S., . . . Aubret, F. (2018). Variation of preferred body temperatures along an altitudinal gradient: A multi-species study. Journal of Thermal Biology, 77, 38-44. doi:https://doi.org/10.1016/j.jtherbio.2018.08.002

Tsuji, J. S. (1988). Seasonal Profiles of Standard Metabolic Rate of Lizards (Sceloporus occidentalis) in Relation to Latitude. Physiological Zoology, 61(3), 230-240.

Üzüm, N., Ilgaz, Ç., Avcı, A., Candan, K., Güler, H., & Kumlutaş, Y. (2018). Comparison of the body size and age structure of Lebanon lizard, Phoenicolacerta laevis (Gray, 1838) at different altitudes in Turkey. Vertebrate Zoology, 68, 83-90.

Valdecantos, S., Martínez, V., Lobo, F., & Cruz, F. B. (2013). Thermal biology of Liolaemus lizards from the high Andes: Being efficient despite adversity. Journal of Thermal Biology, 38(3), 126-134. doi:https://doi.org/10.1016/j.jtherbio.2012.12.002

Valdéz-González, M. A., & Ramírez-Bautista, A. (2002). Reproductive Characteristics of the Spiny Lizards, Sceloporus horridus and Sceloporus spinosus (Squamata: Phrynosomatidae) from México. Journal of Herpetology, 36(1), 36-43. doi:10.2307/1565799

Valencia-Flores, E., Venegas-Barrera, C. S., Fajardo, V., & Manjarrez, J. (2019). Microgeographic variation in body condition of three Mexican garter snakes in central Mexico. PeerJ, 7, e6601. doi:10.7717/peerj.6601

Van Damme, R., Bauwens, D., Castilla, A. M., & Verheyen, R. F. (1989). Altitudinal variation of the thermal biology and running performance in the lizard Podarcis tiliguerta. Oecologia, 80(4), 516-524. doi:10.1007/BF00380076

Van Damme, R., Bauwens, D., & Verheyen, R. F. (1990). Evolutionary Rigidity of Thermal Physiology: The Case of the Cool Temperate Lizard Lacerta vivipara. Oikos, 57(1), 61-67. doi:10.2307/3565737

Vega-Pérez, A. H. D. d. l., Barrios-Montiel, R., Jiménez-Arcos, V. H., Bautista, A., & Bastiaans, E. (2019). High-mountain altitudinal gradient influences thermal ecology of the Mesquite Lizard (Sceloporus grammicus). Canadian Journal of Zoology, 97(8), 659-668. doi:10.1139/cjz-2018-0263

Verdú-Ricoy, J., Iraeta, P., Salvador, A., & Díaz, J. A. (2014). Phenotypic responses to incubation conditions in ecologically distinct populations of a lacertid lizard: a tale of two phylogeographic lineages. Journal of Zoology, 292(3), 184-191. doi:https://doi.org/10.1111/jzo.12091

Vergilov, V. S., Necheva, V. G., & Zlatkov, B. P. (2018). Reproduction of Snake-eyed Skink Ablepharus kitaibelii (Bibron & Bory de Saint-Vincent, 1833)(Squamata: Scincidae) in Bulgaria. Acta Zoologica Bulgarica, 70(4), 507-516.

Villagrán–Santa Cruz, M., Hernández-Gallegos, O., & Méndez–de la Cruz, F. R. (2009). Reproductive cycle of the lizard Sceloporus mucronatus with comments on intraspecifific geographic variation. Western North American Naturalist, 69(4), 437-446.

Vinegar, M. B. (1975). Demography of the Striped Plateau Lizard, Sceloporus Virgatus. Ecology, 56(1), 172-182. doi:https://doi.org/10.2307/1935309

Vinegar, M. B. (1975). Life History Phenomena in Two Populations of the Lizard Sceloporus undulatus in Southwestern New Mexico. The American Midland Naturalist, 93(2), 388-402. doi:10.2307/2424171

Wang, C.-F., Du, Y., Guo, K., & Ji, X. (2024). Species-Specific Seasonal Shifts in Reproductive Allocation in the Southern Grass Lizard, Takydromus sexlineatus (Lacertidae). Animals, 14(8), 1167.

Wang, Y., Ji, W., Zhao, W., Yu, N., & Liu, N. (2011). Geographic variation in clutch and egg size for the lizard Phrynocephalus przewalskii (Squamata: Agamidae). Asian Herpetological Research, 2(2), 97-102.

Wang, Y., Li, S.-R., Pei, M.-Y., Wu, D.-Y., & Du, W.-G. (2021). Population origin, maternal effects, and hydric conditions during incubation determine embryonic and offspring survival in a desert-dwelling lizard. Oecologia, 196(2), 341-352. doi:10.1007/s00442-021-04932-9

Wang, Z., Xia, Y., & Ji, X. (2011). Clutch Frequency Affects the Offspring Size-Number Trade-Off in Lizards. Plos One, 6(1), e16585. doi:10.1371/journal.pone.0016585

Wapstra, E., & Swain, R. (2001). Geographic and Annual Variation in Life-History Traits in a Temperate Zone Australian Skink. Journal of Herpetology, 35(2), 194-203. doi:10.2307/1566108

Wapstra, E., Swain, R., Jones, S. M., & O'Reilly, J. (1999). Geographic and annual variation in reproductive cycles in the Tasmanian spotted snow skink, Niveoscincus ocellatus (Squamata : Scincidae). Australian Journal of Zoology, 47(6), 539-550. doi:https://doi.org/10.1071/ZO99038

Wapstra, E., Swain, R., & O'Reilly, J. M. (2001). Geographic Variation in Age and Size at Maturity in a Small Australian Viviparous Skink. Copeia, 2001(3), 646-655, 610.

Weathers, W. W., & White, F. N. (1972). Hematological Observations on Populations of the Lizard Sceloporus occidentalis from Sea Level and Altitude. Herpetologica, 28(2), 172-175.

Wei‐Guo Du, Daniel A. Warner, Tracy Langkilde, Travis Robbins, & Richard Shine. (2010). The Physiological Basis of Geographic Variation in Rates of Embryonic Development within a Widespread Lizard Species. The American Naturalist, 176(4), 522-528. doi:10.1086/656270

Wilson, B. S. (1991). Latitudinal Variation in Activity Season Mortality Rates of the Lizard Uta Stansburiana. Ecological Monographs, 61(4), 393-414. doi:https://doi.org/10.2307/2937048

Wu, Q., Dang, W., Hu, Y.-C., & Lu, H.-L. (2018). Altitude influences thermal ecology and thermal sensitivity of locomotor performance in a toad-headed lizard. Journal of Thermal Biology, 71, 136-141. doi:https://doi.org/10.1016/j.jtherbio.2017.11.005

Wu, Q., Richard, M., Rutschmann, A., Miles, D. B., & Clobert, J. (2019). Environmental variation mediates the prevalence and co-occurrence of parasites in the common lizard, Zootoca vivipara. BMC Ecology, 19(1), 44. doi:10.1186/s12898-019-0259-3

Yıldırım, E., Kumlutaş, Y., Candan, K., & Ilgaz, Ç. (2021). The study on the relationships between the age structure and body size of the Bridled Skink, Heremites vittatus,(Oliver, 1804) from different altitudes in Turkey. Journal of the Institute of Science and Technology, 11(2), 906-915.

Yu, W., Zhu, Z., Zhao, X., Cui, S., Liu, Z., & Zeng, Z. (2022). Altitudinal variation in life-history features of a Qinghai-Tibetan Plateau lizard. Current Zoology, 69(3), 284-293. doi:10.1093/cz/zoac052

Žagar, A., Simčič, T., Dajčman, U., & Megía-Palma, R. (2022). Parasitemia and elevation as predictors of hemoglobin concentration and antioxidant capacity in two sympatric lizards. Comparative Biochemistry and Physiology Part A: Molecular & Integrative Physiology, 270, 111233. doi:https://doi.org/10.1016/j.cbpa.2022.111233

Zamora-Camacho, F. J., Reguera, S., & Moreno-Rueda, G. (2015). Does tail autotomy affect thermoregulation in an accurately thermoregulating lizard? Lessons from a 2200-m elevational gradient. Journal of Zoology, 297(3), 204-210. doi:https://doi.org/10.1111/jzo.12266

Zamora-Camacho, F. J., Reguera, S., & Moreno-Rueda, G. (2016). Elevational variation in body-temperature response to immune challenge in a lizard. PeerJ, 4, e1972.

Zamora-Camacho, F. J., Reguera, S., & Moreno-Rueda, G. (2016). Thermoregulation in the lizard Psammodromus algirus along a 2200-m elevational gradient in Sierra Nevada (Spain). International Journal of Biometeorology, 60(5), 687-697. doi:10.1007/s00484-015-1063-1

Zamora‐Camacho, F. J., Reguera, S., & Moreno‐Rueda, G. (2014). Bergmann's Rule rules body size in an ectotherm: heat conservation in a lizard along a 2200‐metre elevational gradient. Journal of Evolutionary Biology, 27(12), 2820-2828. doi:10.1111/jeb.12546

Zamora-Camacho, F. J., Reguera, S., Rubiño-Hispán, M. V., & Moreno-Rueda, G. (2014). Effects of Limb Length, Body Mass, Gender, Gravidity, and Elevation on Escape Speed in the Lizard Psammodromus algirus. Evolutionary Biology, 41(4), 509-517. doi:10.1007/s11692-014-9285-4

Zamora-Camacho, F. J., Reguera, S., Rubiño-Hispán, M. V., & Moreno-Rueda, G. (2014). Eliciting an immune response reduces sprint speed in a lizard. Behavioral Ecology, 26(1), 115-120. doi:10.1093/beheco/aru170

Zamora-Camacho, F. J., Rubiño-Hispán, M. V., Reguera, S., & Moreno-Rueda, G. (2015). Thermal dependence of sprint performance in the lizard Psammodromus algirus along a 2200-meter elevational gradient: Cold-habitat lizards do not perform better at low temperatures. Journal of Thermal Biology, 52, 90-96. doi:https://doi.org/10.1016/j.jtherbio.2015.06.003

Zeng, Z., Liu, Z., Wei, J., Zhang, X., & Du, W. (2022). Desertification drives the shift in egg size-number trade-off in an agamid lizard. Asian Herpetological Research, 13(4), 284-291.

Zeng, Z.-G., Zhao, J.-M., & Sun, B.-J. (2013). Life history variation among geographically close populations of the toad-headed lizard (Phrynocephalus przewalskii): Exploring environmental and physiological associations. Acta Oecologica, 51, 28-33. doi:https://doi.org/10.1016/j.actao.2013.05.004

Zhang, W., Li, N., Tang, X., Liu, N., & Zhao, W. (2018). Changes in intestinal microbiota across an altitudinal gradient in the lizard Phrynocephalus vlangalii. Ecology and evolution, 8(9), 4695-4703. doi:https://doi.org/10.1002/ece3.4029

Zhang, X., Men, S., Jia, L., Tang, X., Storey, K. B., Niu, Y., & Chen, Q. (2023). Comparative metabolomics analysis reveals high-altitude adaptations in a toad-headed viviparous lizard, Phrynocephalus vlangalii. Frontiers in Zoology, 20(1), 35. doi:10.1186/s12983-023-00513-z

Zhang, Y., Liang, S., He, J., Bai, Y., Niu, Y., Tang, X., . . . Chen, Q. (2015). Oxidative stress and antioxidant status in a lizard Phrynocephalus vlangalii at different altitudes or acclimated to hypoxia. Comparative Biochemistry and Physiology Part A: Molecular & Integrative Physiology, 190, 9-14. doi:https://doi.org/10.1016/j.cbpa.2015.08.013

ZUFFI, M. A. L., GENTILLI, A., CECCHINELLI, E., PUPIN, F., BONNET, X., FILIPPI, E., . . . FASOLA, M. (2009). Geographic variation of body size and reproductive patterns in Continental versus Mediterranean asp vipers, Vipera aspis. Biological Journal of the Linnean Society, 96(2), 383-391. doi:10.1111/j.1095-8312.2008.01124.x


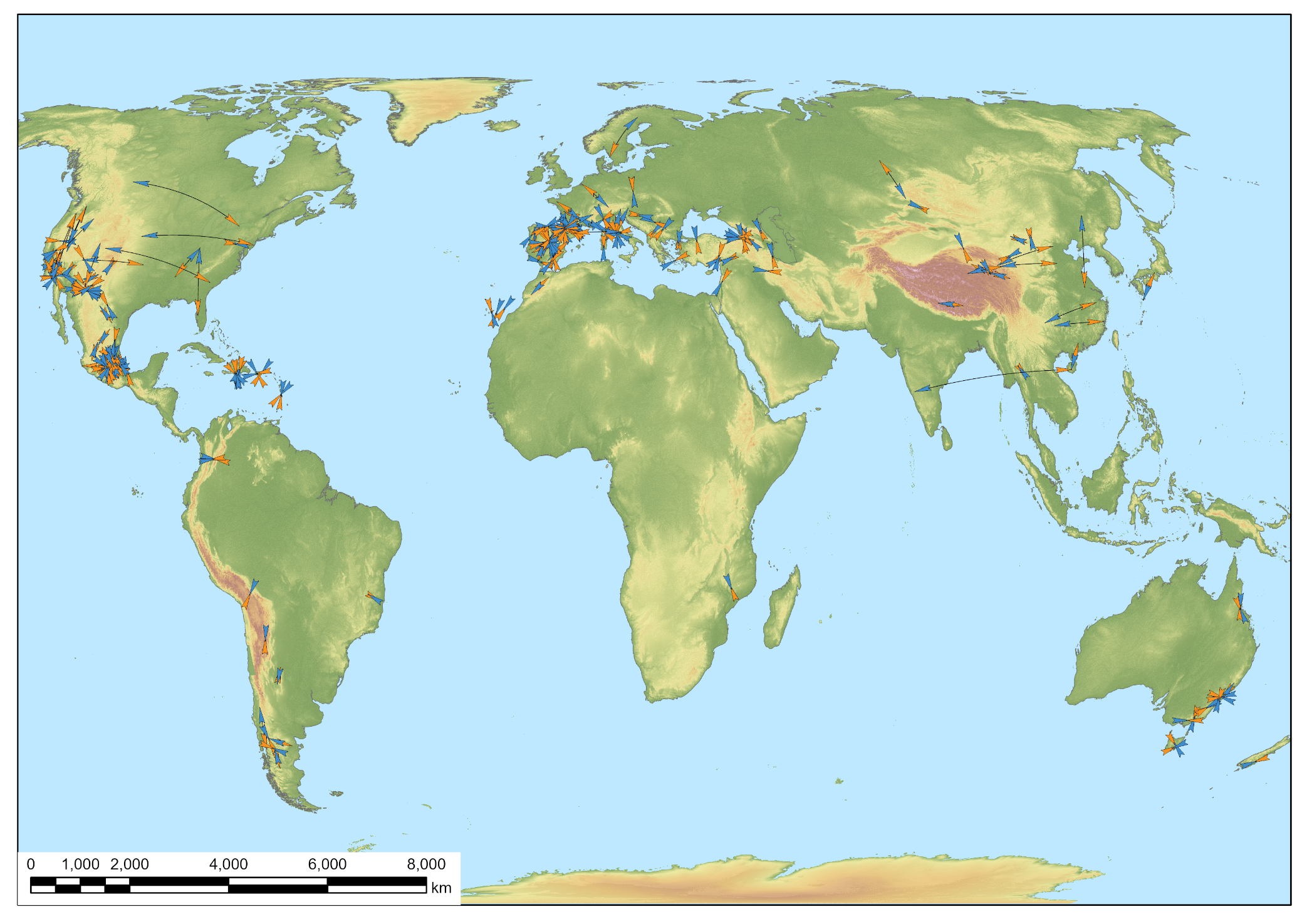


Figure S1 Location of low-high population pairs for which we extracted data used in the meta-analyses (the orange arrowhead ▼ is for the low populations and the blue arrowhead ▲ is for the high population)

Table S2 Summary of differences in mean response ratios (Est) between low and high populations from squamate families included in the meta-analyses

| **Family** | **Adult body size** | | |  | **Adult female body size** | | |  | **Neonate body size** | | |
| --- | --- | --- | --- | --- | --- | --- | --- | --- | --- | --- | --- |
|  | **Est** | **SE** | ***p*** |  | **Est** | **SE** | ***p*** |  | **Est** | **SE** | ***p*** |
| Agamidae | 0.175 | 0.139 | 0.212 |  | 0.025 | 0.036 | 0.706 |  | 0.071 | 0.051 | 0.182 |
| Anolidae | 0.117 | 0.167 | 0.704 |  | - | - | - |  | - | - | - |
| Lacertidae | 0.063 | 0.100 | 0.630 |  | 0.011 | 0.018 | 0.623 |  | -0.007 | 0.040 | 0.856 |
| Phrynosomatidae | 0.012 | 0.129 | 0.922 |  | 0.017 | 0.022 | 0.451 |  | - | - | - |
| Scincidae | -0.026 | 0.106 | 0.807 |  | 0.065 | 0.040 | 0.113 |  | 0.054 | 0.052 | 0.307 |
| Colubridae | 0.017 | 0.119 | 0.883 |  | -0.064 | 0.034 | 0.068 |  | 0.136 | 0.056 | **0.026** |
| Viperidae | -0.026 | 0.142 | 0.853 |  | -0.075 | 0.059 | 0.210 |  | - | - | - |

| **Family** | **Fecundity** | | |  | **T_b_** | | |
| --- | --- | --- | --- | --- | --- | --- | --- |
|  | **Est** | **SE** | ***p*** |  | **Est** | **SE** | ***p*** |
| Agamidae | 0.042 | 0.087 | 0.628 |  | - | - | **-** |
| Anolidae | - | - | - |  | -0.094 | 0.028 | **0.003** |
| Lacertidae | 0.073 | 0.061 | 0.240 |  | -0.043 | 0.022 | 0.068 |
| Liolaemidae | - | - | - |  | -0.026 | 0.026 | 0.330 |
| Phrynosomatidae | 0.032 | 0.059 | 0.593 |  | -0.057 | 0.020 | **0.009** |
| Scincidae | 0.023 | 0.095 | 0.814 |  | - | - | - |
| Colubridae | -0.509 | 0.122 | **<0.001** |  | - | - | - |
| Viperidae | - | - | **-** |  | - | - | - |

Table S3 Summary of differences in mean response ratios (Est) between low and high populations from the two squamate suborders (lizards and snakes)

| **Suborder** | **Adult body size** | | |  | **Adult female body size** | | |
| --- | --- | --- | --- | --- | --- | --- | --- |
|  | **Est** | **SE** | ***p*** |  | **Est** | **SE** | ***p*** |
| Lacertilia | 0.044 | 0.040 | 0.276 |  | 0.019 | 0.012 | 0.120 |
| Serpentes | 0.005 | 0.068 | 0.939 |  | -0.066 | 0.029 | **0.028** |

| **Suborder** | **Neonate body size** | | |  | **Fecundity** | | |
| --- | --- | --- | --- | --- | --- | --- | --- |
|  | **Est** | **SE** | ***p*** |  | **Est** | **SE** | ***p*** |
| Lacertilia | 0.014 | 0.024 | 0.555 |  | 0.044 | 0.032 | 0.179 |
| Serpentes | 0.139 | 0.057 | **0.023** |  | -0.513 | 0.115 | **<0.001** |

Table S4 Summary of differences in the coefficient of variation (Est) between low and high populations from squamate families included in the meta-analyses

| **Family** | **Adult body size** | | |  | **Adult female body size** | | |
| --- | --- | --- | --- | --- | --- | --- | --- |
|  | **Est** | **SE** | ***p*** |  | **Est** | **SE** | ***p*** |
| Agamidae | -0.058 | 0.113 | 0.611 |  | 0.092 | 0.135 | 0.684 |
| Anolidae | 0.166 | 0.237 | 0.484 |  | - | - | - |
| Lacertidae | 0.013 | 0.059 | 0.829 |  | 0.079 | 0.068 | 0.253 |
| Phrynosomatidae | 0.096 | 0.069 | 0.164 |  | -0.005 | 0.082 | 0.953 |
| Scincidae | -0.075 | 0.111 | 0.498 |  | -0.087 | 0.147 | 0.558 |
| Colubridae | 0.025 | 0.106 | 0.811 |  | -0.078 | 0.127 | 0.545 |
| Viperidae | 0.295 | 0.184 | 0.113 |  | 0.494 | 0.226 | **0.034** |

| **Family** | **Fecundity** | | |  | **T_b_** | | |
| --- | --- | --- | --- | --- | --- | --- | --- |
|  | **Est** | **SE** | ***p*** |  | **Est** | **SE** | ***p*** |
| Agamidae | 0.134 | 0.101 | 0.192 |  | - | - | - |
| Anolidae | - | - | - |  | 0.421 | 0.227 | 0.074 |
| Lacertidae | -0.054 | 0.074 | 0.471 |  | 0.081 | 0.198 | 0.686 |
| Liolaemidae | - | - | - |  | 0.208 | 0.244 | 0.401 |
| Phrynosomatidae | 0.011 | 0.074 | 0.885 |  | 0.194 | 0.186 | 0.305 |
| Scincidae | 0.095 | 0.113 | 0.407 |  | - | - | - |
| Colubridae | 0.021 | 0.154 | 0.891 |  | - | - | - |

Table S5 Summary of differences in the coefficient of variation (Est) between low and high populations from the two squamate suborders (lizards and snakes)

| **Suborder** | **Adult body size** | | |  | **Adult female body size** | | |  | **Fecundity** | | |
| --- | --- | --- | --- | --- | --- | --- | --- | --- | --- | --- | --- |
|  | **Est** | **SE** | ***p*** |  | **Est** | **SE** | ***p*** |  | **Est** | **SE** | ***p*** |
| Lacertilia | 0.026 | 0.040 | 0.522 |  | 0.036 | 0.046 | 0.428 |  | 0.024 | 0.043 | 0.580 |
| Serpentes | 0.089 | 0.091 | 0.331 |  | 0.061 | 0.111 | 0.548 |  | 0.022 | 0.153 | 0.885 |

Table S6 Sensitivity analysis for the within-study sampling variance correlation for mean effect sizes

| **Mean effect size** | | | | | |
| --- | --- | --- | --- | --- | --- |
| **Adult body size** | | | | | |
| **Correlation (ρ)** | **Overall effect (β_0_)** | **S.E.** | ***p*-value** | **Lower CI** | **Upper CI** |
| 0.3 | 0.025 | 0.020 | 0.217 | -0.015 | 0.056 |
| 0.5 | 0.027 | 0.020 | 0.196 | -0.014 | 0.067 |
| 0.7 | 0.028 | 0.021 | 0.184 | -0.013 | 0.068 |
| 0.9 | 0.028 | 0.021 | 0.180 | -0.013 | 0.069 |

| **Adult female body size** | | | | | |
| --- | --- | --- | --- | --- | --- |
| **Correlation (ρ)** | **Overall effect (β_0_)** | **S.E.** | ***p*-value** | **Lower CI** | **Upper CI** |
| 0.3 | 0.007 | 0.011 | 0.545 | -0.015 | 0.029 |
| 0.5 | 0.007 | 0.011 | 0.545 | -0.016 | 0.029 |
| 0.7 | 0.007 | 0.011 | 0.545 | -0.016 | 0.029 |
| 0.9 | 0.007 | 0.011 | 0.545 | -0.016 | 0.030 |

| **Neonate body size** | | | | | |
| --- | --- | --- | --- | --- | --- |
| **Correlation (ρ)** | **Overall effect (β_0_)** | **S.E.** | ***p*-value** | **Lower CI** | **Upper CI** |
| 0.3 | 0.037 | 0.027 | 0.178 | -0.017 | 0.091 |
| 0.5 | 0.037 | 0.027 | 0.176 | -0.017 | 0.091 |
| 0.7 | 0.037 | 0.027 | 0.174 | -0.017 | 0.091 |
| 0.9 | 0.037 | 0.027 | 0.172 | -0.017 | 0.091 |

| **Mean age** | | | | | |
| --- | --- | --- | --- | --- | --- |
| **Correlation (ρ)** | **Overall effect (β_0_)** | **S.E.** | ***p*-value** | **Lower CI** | **Upper CI** |
| 0.3 | 0.032 | 0.054 | 0.564 | -0.083 | 0.146 |
| 0.5 | 0.032 | 0.054 | 0.564 | -0.083 | 0.146 |
| 0.7 | 0.032 | 0.054 | 0.564 | -0.083 | 0.146 |
| 0.9 | 0.032 | 0.054 | 0.564 | -0.083 | 0.146 |

| **Egg size** | | | | | |
| --- | --- | --- | --- | --- | --- |
| **Correlation (ρ)** | **Overall effect (β_0_)** | **S.E.** | ***p*-value** | **Lower CI** | **Upper CI** |
| 0.3 | 0.094 | 0.051 | 0.084 | -0.014 | 0.203 |
| 0.5 | 0.094 | 0.051 | 0.085 | -0.015 | 0.203 |
| 0.7 | 0.095 | 0.051 | 0.085 | -0.015 | 0.204 |
| 0.9 | 0.095 | 0.051 | 0.085 | -0.015 | 0.204 |

| **Fecundity** | | | | | |
| --- | --- | --- | --- | --- | --- |
| **Correlation (ρ)** | **Overall effect (β_0_)** | **S.E.** | ***p*-value** | **Lower CI** | **Upper CI** |
| 0.3 | 0.001 | 0.038 | 0.978 | -0.073 | 0.076 |
| 0.5 | 0.001 | 0.038 | 0.981 | -0.074 | 0.075 |
| 0.7 | 0.001 | 0.038 | 0.984 | -0.074 | 0.075 |
| 0.9 | 0.001 | 0.038 | 0.987 | -0.074 | 0.075 |

| **Body temperature** | | | | | |
| --- | --- | --- | --- | --- | --- |
| **Correlation (ρ)** | **Overall effect (β_0_)** | **S.E.** | ***p*-value** | **Lower CI** | **Upper CI** |
| 0.3 | -0.052 | 0.009 | <0.001 | -0.071 | -0.034 |
| 0.5 | -0.053 | 0.009 | <0.001 | -0.071 | -0.034 |
| 0.7 | -0.053 | 0.009 | <0.001 | -0.071 | -0.035 |
| 0.9 | -0.053 | 0.009 | <0.001 | -0.071 | -0.035 |

| **Lower critical temperature** | | | | | |
| --- | --- | --- | --- | --- | --- |
| **Correlation (ρ)** | **Overall effect (β_0_)** | **S.E.** | ***p*-value** | **Lower CI** | **Upper CI** |
| 0.3 | -0.059 | 0.049 | 0.236 | -0.160 | 0.041 |
| 0.5 | -0.061 | 0.051 | 0.240 | -0.165 | 0.043 |
| 0.7 | -0.060 | 0.052 | 0.258 | -0.167 | 0.047 |
| 0.9 | -0.057 | 0.053 | 0.294 | -0.166 | 0.052 |

| **Upper critical temperature** | | | | | |
| --- | --- | --- | --- | --- | --- |
| **Correlation (ρ)** | **Overall effect (β_0_)** | **S.E.** | ***p*-value** | **Lower CI** | **Upper CI** |
| 0.3 | -0.007 | 0.006 | 0.246 | -0.020 | 0.005 |
| 0.5 | -0.007 | 0.006 | 0.239 | -0.020 | 0.005 |
| 0.7 | -0.008 | 0.006 | 0.232 | -0.021 | 0.005 |
| 0.9 | -0.008 | 0.006 | 0.224 | -0.021 | 0.005 |

Table S7 Sensitivity analysis for the within-study sampling variance correlation for coefficient of variation

| **Coefficient of variation** | | | | | |
| --- | --- | --- | --- | --- | --- |
| **Adult body size** | | | | | |
| **Correlation (ρ)** | **Overall effect (β_0_)** | **S.E.** | ***p*-value** | **Lower CI** | **Upper CI** |
| 0.3 | 0.033 | 0.038 | 0.378 | -0.041 | 0.107 |
| 0.5 | 0.032 | 0.039 | 0.411 | -0.045 | 0.109 |
| 0.7 | 0.030 | 0.042 | 0.464 | -0.051 | 0.112 |
| 0.9 | 0.028 | 0.045 | 0.538 | -0.061 | 0.117 |

| **Adult female body size** | | | | | |
| --- | --- | --- | --- | --- | --- |
| **Correlation (ρ)** | **Overall effect (β_0_)** | **S.E.** | ***p*-value** | **Lower CI** | **Upper CI** |
| 0.3 | 0.040 | 0.043 | 0.345 | -0.044 | 0.125 |
| 0.5 | 0.041 | 0.044 | 0.345 | -0.045 | 0.128 |
| 0.7 | 0.042 | 0.045 | 0.347 | -0.046 | 0.130 |
| 0.9 | 0.043 | 0.046 | 0.352 | -0.048 | 0.133 |

| **Fecundity** | | | | | |
| --- | --- | --- | --- | --- | --- |
| **Correlation (ρ)** | **Overall effect (β_0_)** | **S.E.** | ***p*-value** | **Lower CI** | **Upper CI** |
| 0.3 | 0.041 | 0.040 | 0.302 | -0.037 | 0.120 |
| 0.5 | 0.044 | 0.039 | 0.262 | -0.034 | 0.122 |
| 0.7 | 0.045 | 0.041 | 0.269 | -0.035 | 0.126 |
| 0.9 | 0.044 | 0.043 | 0.308 | -0.041 | 0.130 |

| **Body temperature** | | | | | |
| --- | --- | --- | --- | --- | --- |
| **Correlation (ρ)** | **Overall effect (β_0_)** | **S.E.** | ***p*-value** | **Lower CI** | **Upper CI** |
| 0.3 | 0.209 | 0.084 | 0.016 | 0.040 | 0.378 |
| 0.5 | 0.209 | 0.084 | 0.016 | 0.040 | 0.378 |
| 0.7 | 0.210 | 0.084 | 0.016 | 0.041 | 0.379 |
| 0.9 | 0.210 | 0.084 | 0.016 | 0.041 | 0.379 |

Table S8 Summary of moderators examining the importance of mean latitude (mLat) in single-moderator meta-regression of mean effect size and in interaction with elevation range (elvR) for all traits where meta-regressions were performed

| **Moderator↓** | **Adult body size** | | | | | | |
| --- | --- | --- | --- | --- | --- | --- | --- |
|  | **Mean latitude only model** | | |  | **Mean latitude × elevation range** | | |
|  | **Est** | **SE** | ***p*** |  | **Est** | **SE** | ***p*** |
| elvR | - | - | **-** |  | -0.081 | 0.098 | 0.407 |
| mLat | -0.000 | 0.002 | 0.901 |  | 0.001 | 0.003 | 0.728 |
| elvR*mLat | - | - | **-** |  | 0.002 | 0.003 | 0.398 |
| **Moderator↓** | **Adult female body size** | | | | | | |
|  | **Mean latitude only model** | | |  | **Mean latitude × elevation range** | | |
|  | **Est** | **SE** | ***p*** |  | **Est** | **SE** | ***p*** |
| elvR | - | - | **-** |  | -0.017 | 0.058 | 0.777 |
| mLat | 0.002 | 0.001 | 0.163 |  | 0.002 | 0.002 | 0.170 |
| elvR*mLat | - | - | **-** |  | 0.000 | 0.002 | 0.869 |
| **Moderator↓** | **Neonate body size** | | | | | | |
|  | **Mean latitude only model** | | |  | **Mean latitude × elevation range** | | |
|  | **Est** | **SE** | ***p*** |  | **Est** | **SE** | ***p*** |
| elvR | - | - | **-** |  | -0.283 | 0.186 | 0.105 |
| mLat | 0.005 | 0.003 | 0.079 |  | 0.008 | 0.003 | **0.015** |
| elvR*mLat | - | - | **-** |  | 0.008 | 0.005 | 0.130 |
| **Moderator↓** | **Fecundity** | | | | | | |
|  | **Mean latitude only model** | | |  | **Mean latitude × elevation range** | | |
|  | **Est** | **SE** | ***p*** |  | **Est** | **SE** | ***p*** |
| elvR | - | - | **-** |  | 0.109 | 0.167 | 0.515 |
| mLat | -0.002 | 0.005 | 0.657 |  | -0.004 | 0.005 | 0.448 |
| elvR*mLat | - | - | **-** |  | -0.006 | 0.005 | 0.233 |
| **Moderator↓** | **Body temperature** | | | | | | |
|  | **Mean latitude only model** | | |  | **Mean latitude × elevation range** | | |
|  | **Est** | **SE** | ***p*** |  | **Est** | **SE** | ***p*** |
| elvR | - | - | **-** |  | 0.096 | 0.052 | 0.073 |
| mLat | 0.002 | 0.001 | **0.033** |  | 0.001 | 0.001 | 0.103 |
| elvR*mLat | - | - | **-** |  | -0.003 | 0.001 | **0.026** |

Table S9 Summary of moderators examining the importance of mean latitude (mLat) in single-moderator meta-regression of effect size variation and in interaction with elevation range (elvR) for all traits where meta-regressions were performed

| **Moderator↓** | **Adult body size variation** | | | | | | |
| --- | --- | --- | --- | --- | --- | --- | --- |
|  | **Mean latitude only model** | | |  | **Mean latitude × elevation range** | | |
|  | **Est** | **SE** | ***p*** |  | **Est** | **SE** | ***p*** |
| elvR | - | - | **-** |  | -0.236 | 0.197 | 0.232 |
| mLat | -0.002 | 0.004 | 0.669 |  | 0.001 | 0.004 | 0.849 |
| elvR*mLat | - | - | **-** |  | 0.006 | 0.005 | 0.238 |
| **Moderator↓** | **Adult female body size variation** | | | | | | |
|  | **Mean latitude only model** | | |  | **Mean latitude × elevation range** | | |
|  | **Est** | **SE** | ***p*** |  | **Est** | **SE** | ***p*** |
| elvR | - | - | **-** |  | -0.234 | 0.211 | 0.270 |
| mLat | 0.002 | 0.005 | 0.720 |  | 0.004 | 0.005 | 0.421 |
| elvR*mLat | - | - | **-** |  | 0.007 | 0.006 | 0.241 |
| **Moderator↓** | **Fecundity variation** | | | | | | |
|  | **Mean latitude only model** | | |  | **Mean latitude × elevation range** | | |
|  | **Est** | **SE** | ***p*** |  | **Est** | **SE** | ***p*** |
| elvR | - | - | **-** |  | -0.279 | 0.239 | 0.248 |
| mLat | -0.002 | 0.005 | 0.735 |  | 0.002 | 0.005 | 0.725 |
| elvR*mLat | - | - | **-** |  | 0.009 | 0.007 | 0.191 |
| **Moderator↓** | **Body temperature variation** | | | | | | |
|  | **Mean latitude only model** | | |  | **Mean latitude × elevation range** | | |
|  | **Est** | **SE** | ***p*** |  | **Est** | **SE** | ***p*** |
| elvR | - | - | **-** |  | -0.013 | 0.579 | 0.982 |
| mLat | -0.013 | 0.008 | 0.120 |  | -0.014 | 0.009 | 0.131 |
| elvR*mLat | - | - | **-** |  | -0.000 | 0.016 | 0.983 |


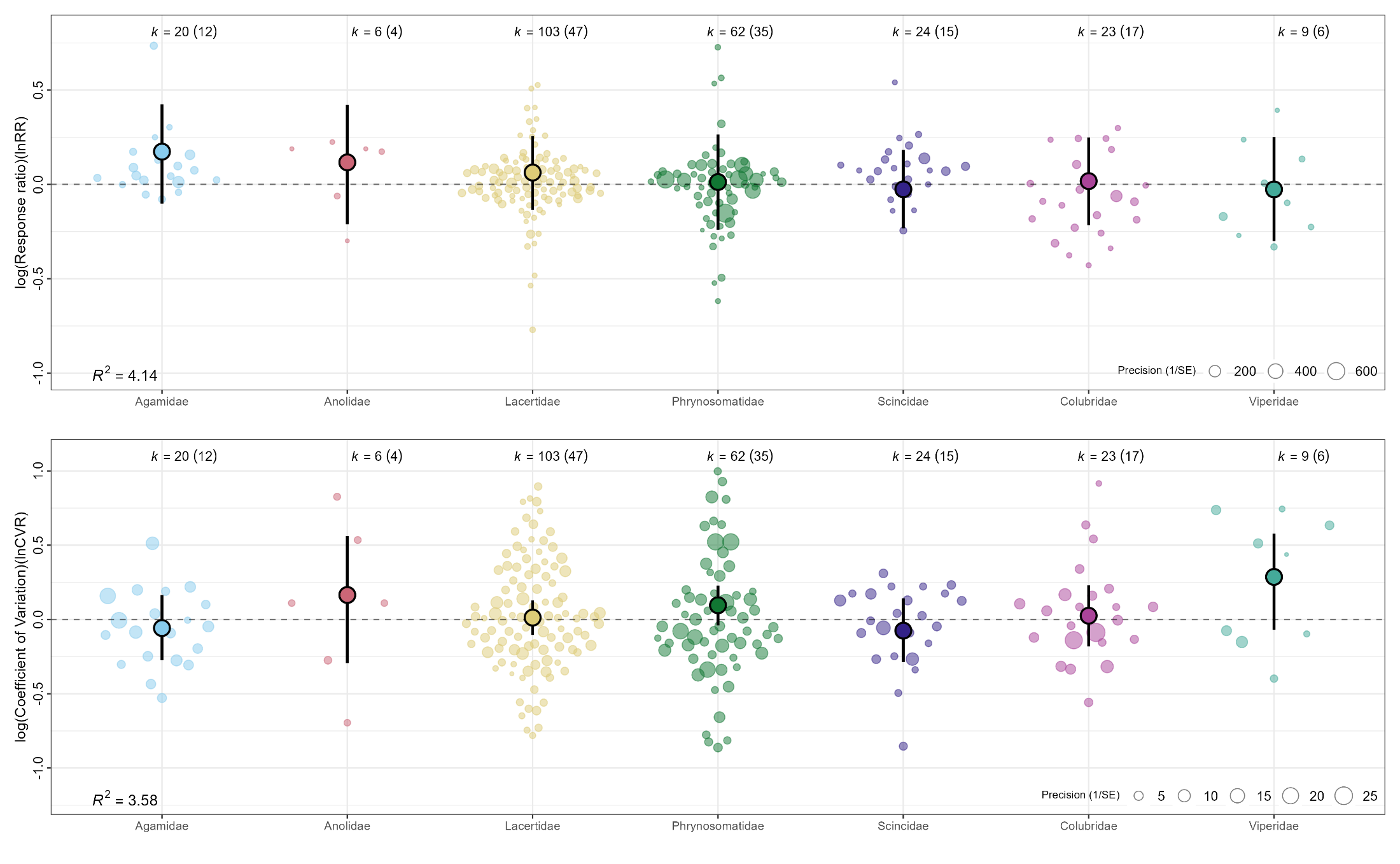


Figure S2 Plots for differences in mean and variation of adult body size (with confidence intervals in black lines) between low and high elevation populations in squamate families


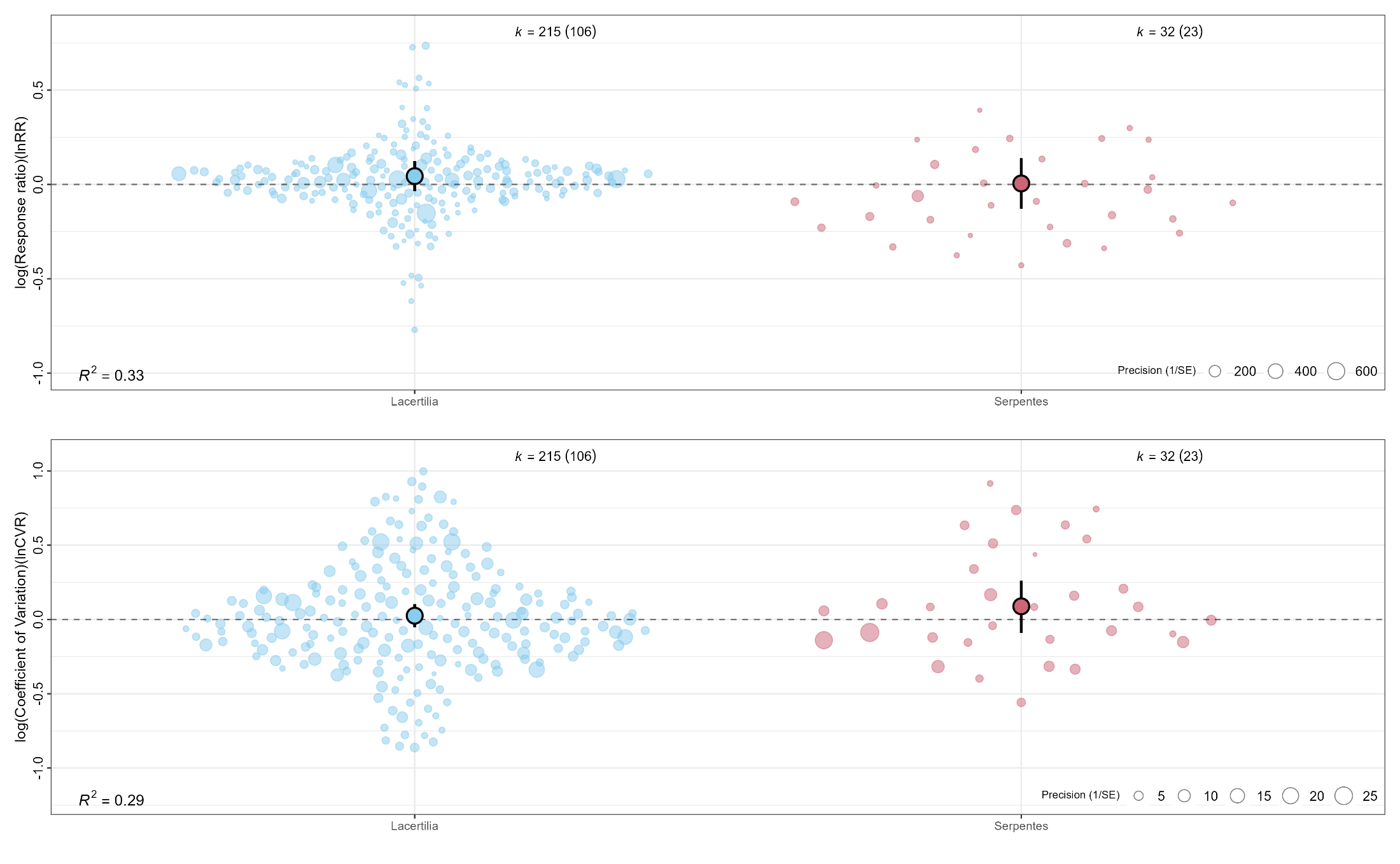


Figure S3 Plots for differences in mean and variation of adult body size (with confidence intervals in black lines) between low and high elevation populations in squamate suborders


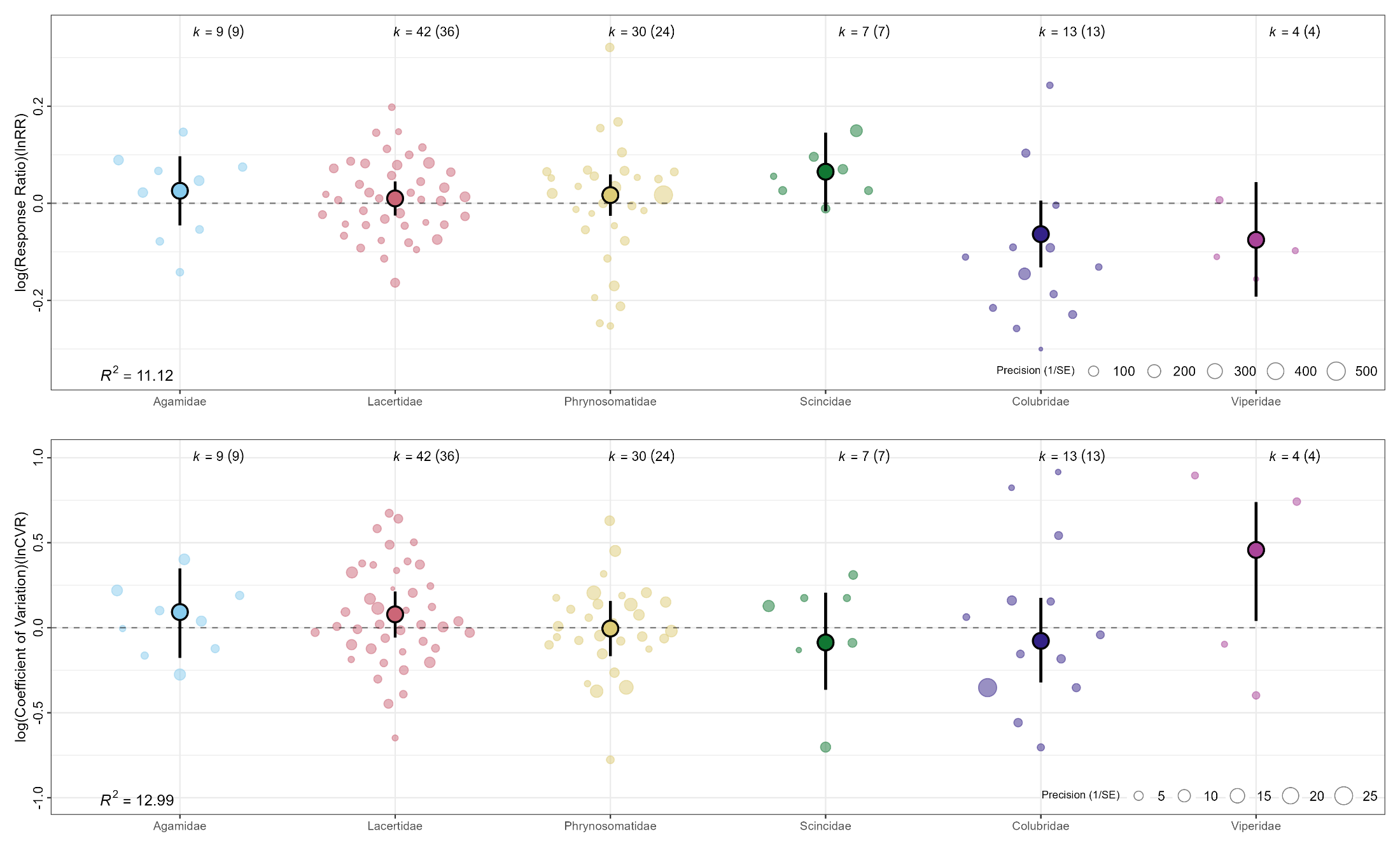


Figure S4 Plots for differences in mean and variation of adult female body size (with confidence intervals in black lines) between low and high elevation populations in squamate families


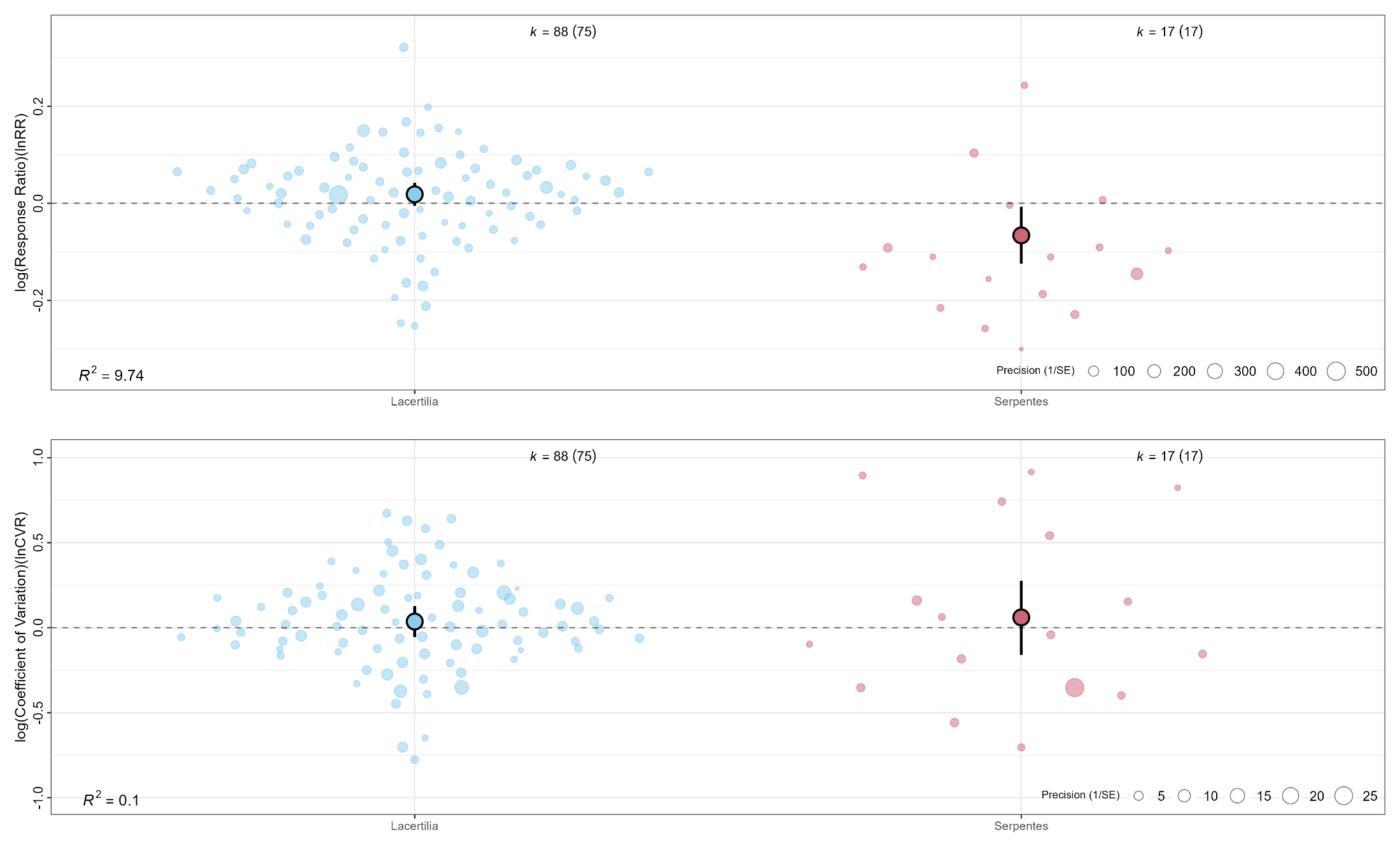


Figure S5 Plots for differences in mean and variation of adult female body size (with confidence intervals in black lines) between low and high elevation populations in squamate suborders


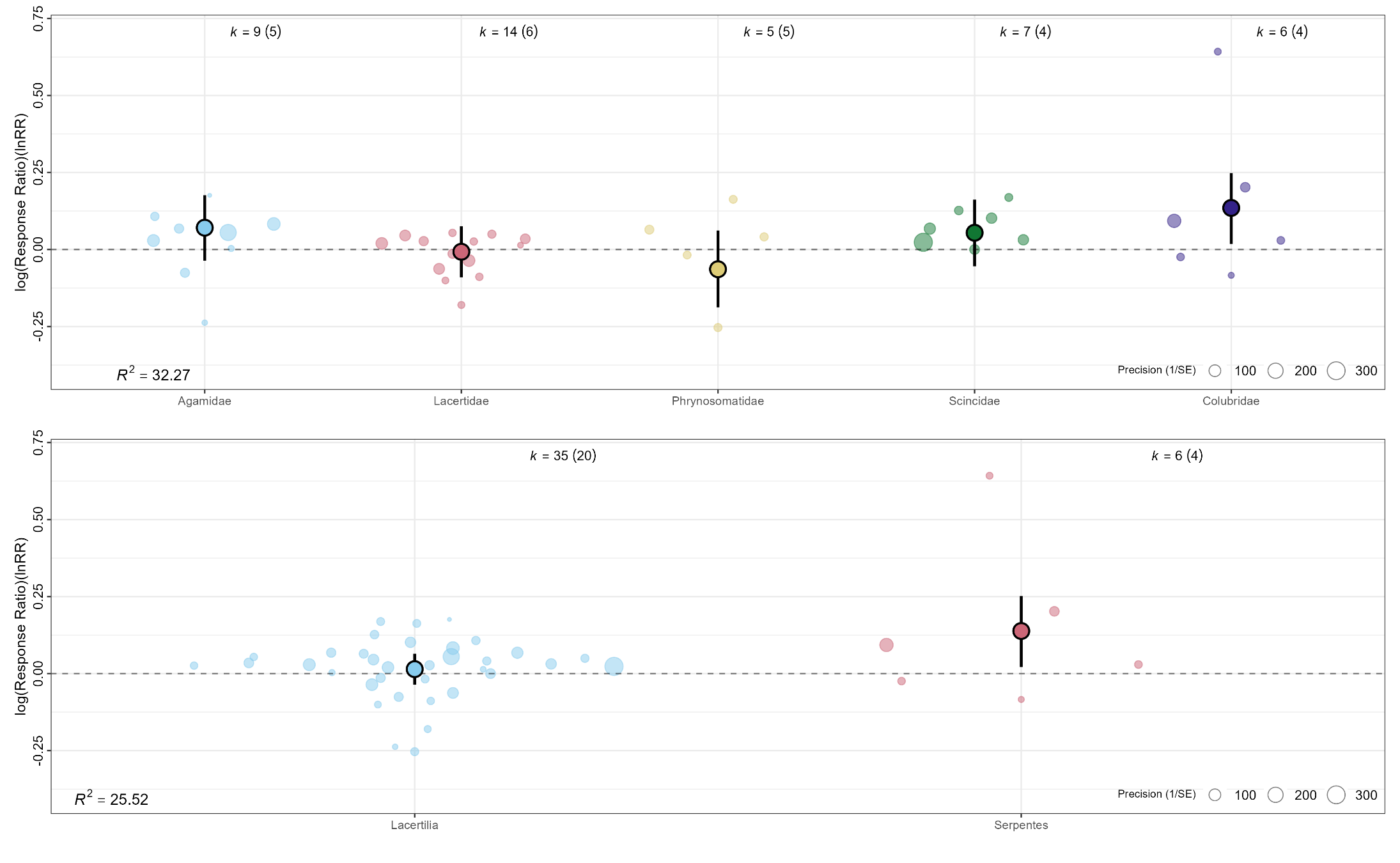


Figure S6 Plots for differences in mean neonate body size (with confidence intervals in black lines) between low and high elevation populations in squamate families and suborders


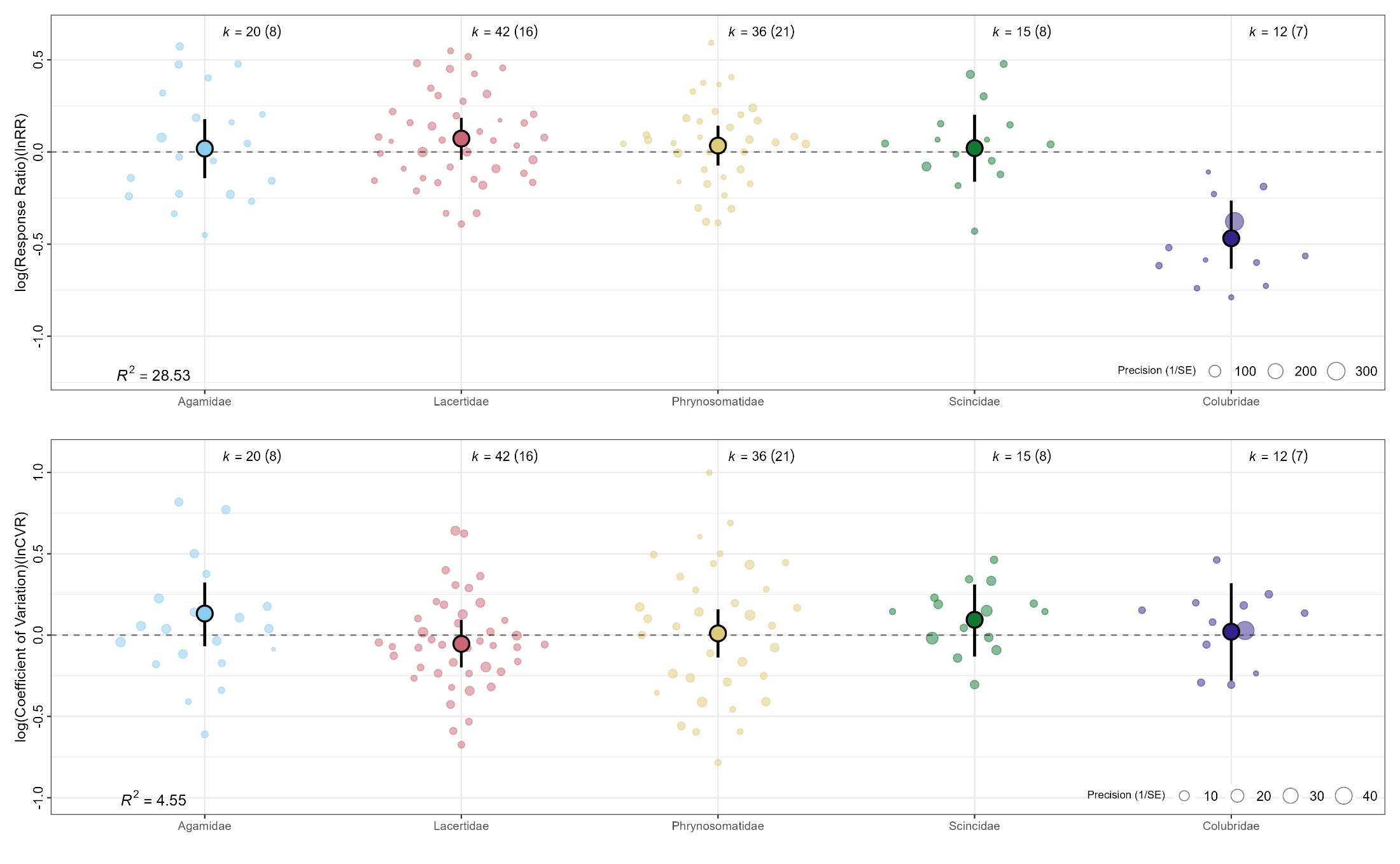


Figure S7 Plots for differences in mean and variation of fecundity (with confidence intervals in black lines) between low and high elevation populations in squamate families


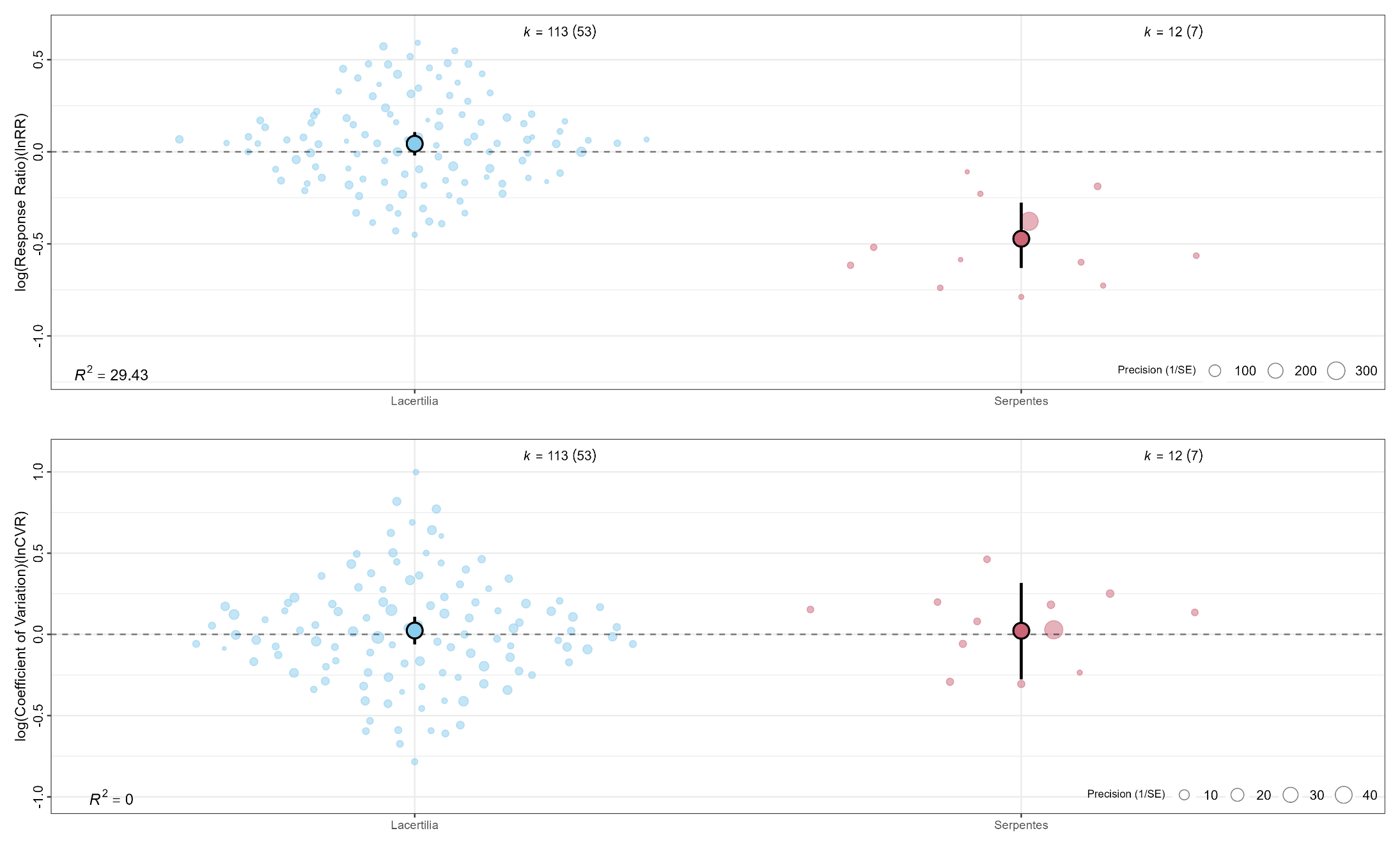


Figure S8 Plots for differences in mean and variation of fecundity between low and high elevation populations in squamate suborders


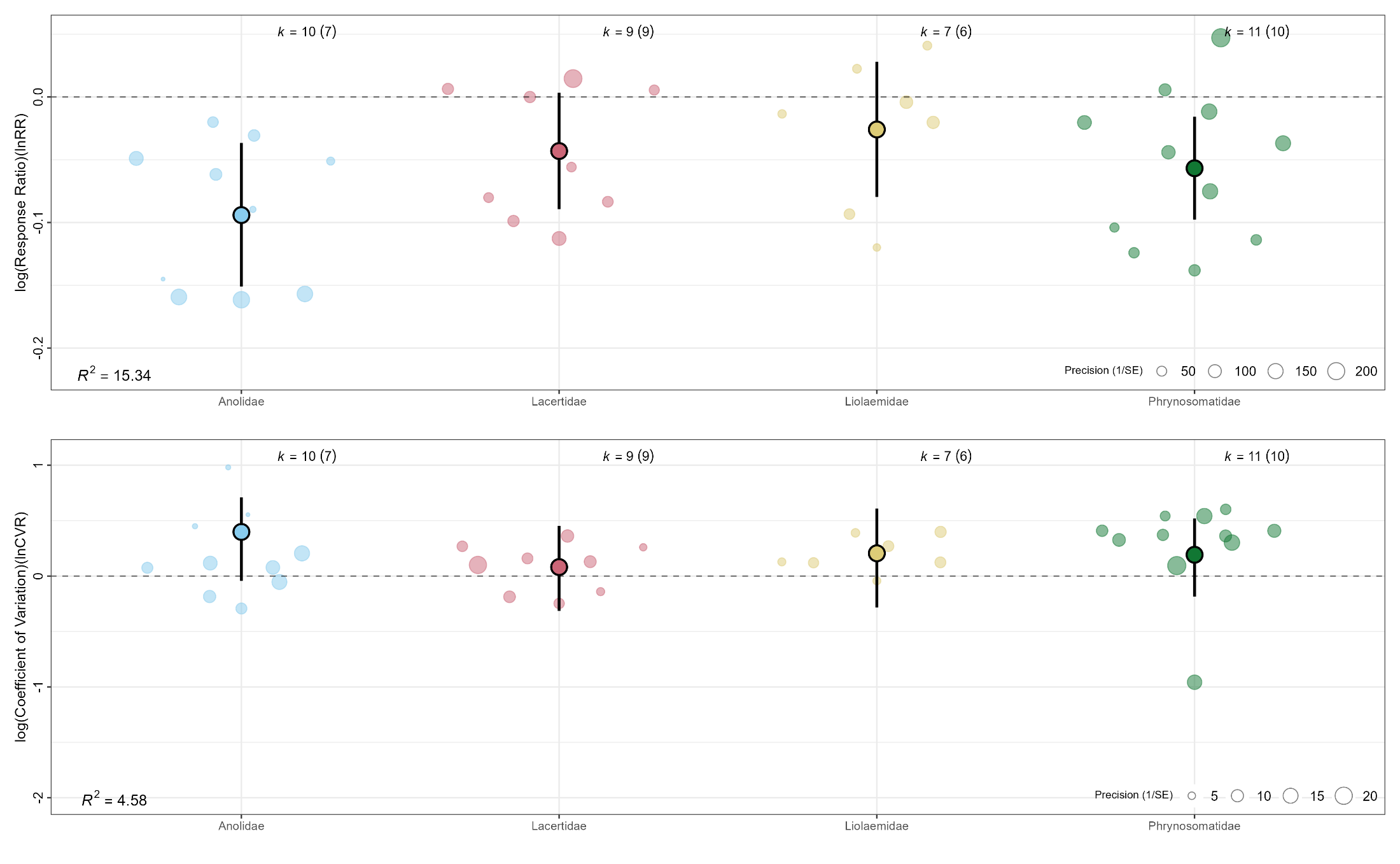


Figure S9 Plots for differences in mean and variation of body temperature (with confidence intervals in black lines) between low and high elevation populations in squamate families


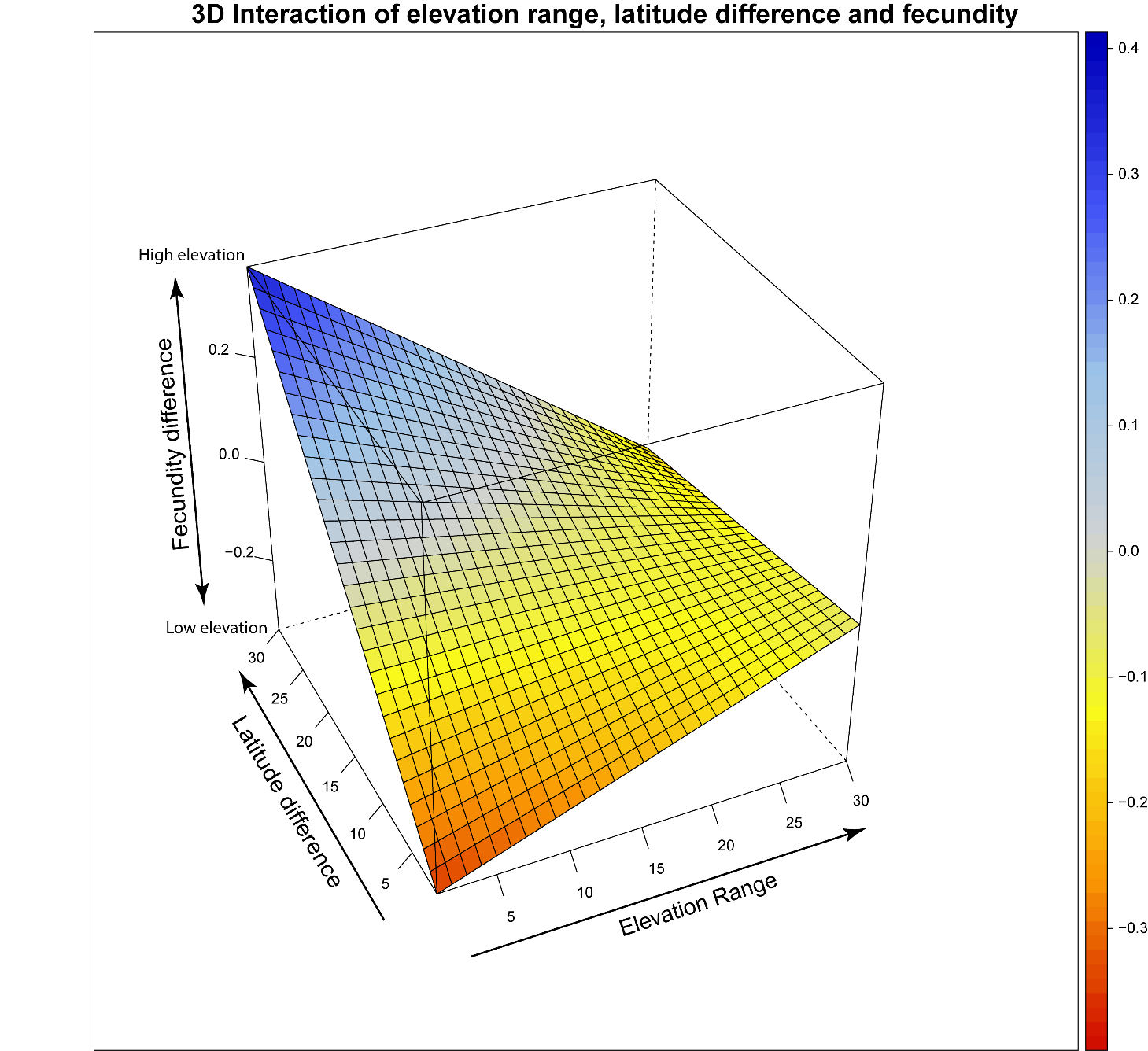


Figure S10 Surface plot illustrating the complex effect of elevation range and latitudinal difference on fecundity of squamates. The fecundity difference axis is symmetric around 0, negative values reflect an increase in fecundity towards lower elevations, while positive values reflect an increase in fecundity towards higher elevations. Elevation range is defined as the log-transformed difference between the elevation of the highest populations and the elevation of the lowest population. Latitude difference is the log-transformed absolute difference between the location of the low altitude population and the high altitude population


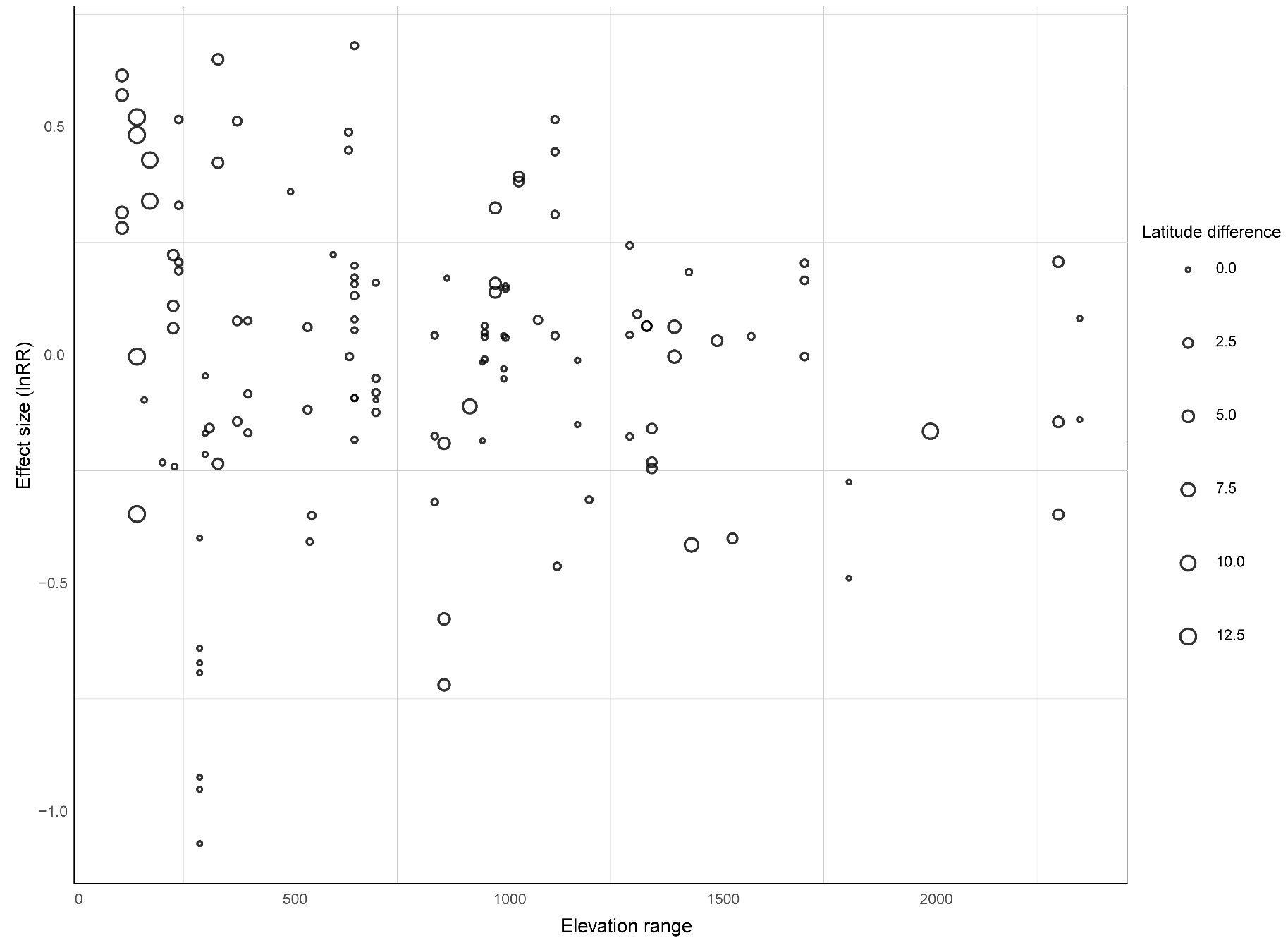


Figure S11 Scatterplot showing the relationship between fecundity difference of low-high elevation population pairs and latitude difference. Point size indicates the latitudinal difference between populations (in ° of latitude). Positive effect sizes indicate higher fecundity in high-elevation populations; negative values indicate higher fecundity in low elevation populations


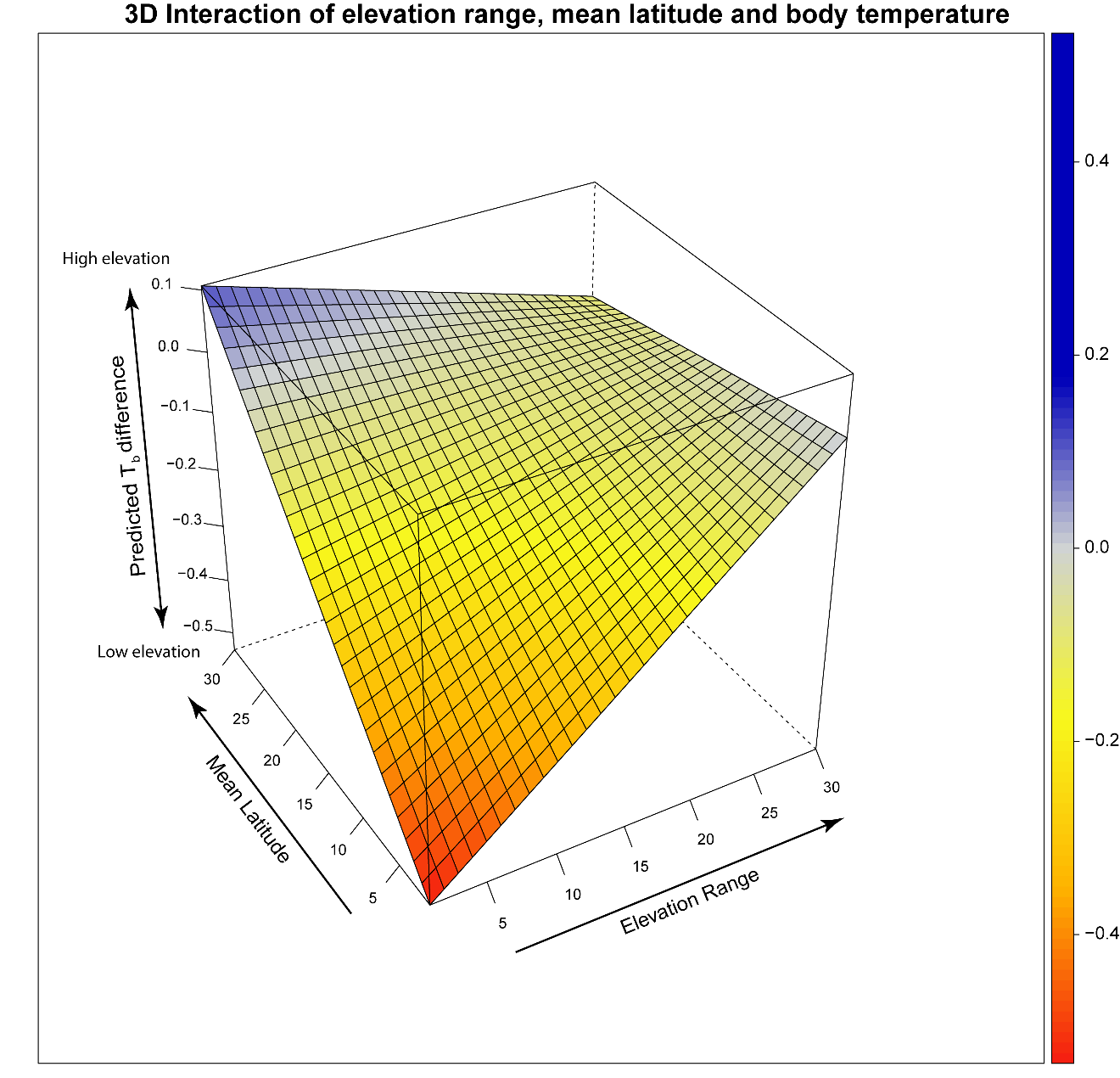


Figure S12 Surface plot illustrating the complex effect of elevation range and mean latitude on the body temperature of squamates. The body temperature difference axis is symmetric around 0, negative values reflect an increase in body temperature towards lower elevations, while positive values reflect an increase in body temperature towards higher elevations. Elevation range is defined as the log-transformed difference between the elevation of the highest populations and the elevation of the lowest population. Mean latitude is the log-transformed absolute mean of the population pairs


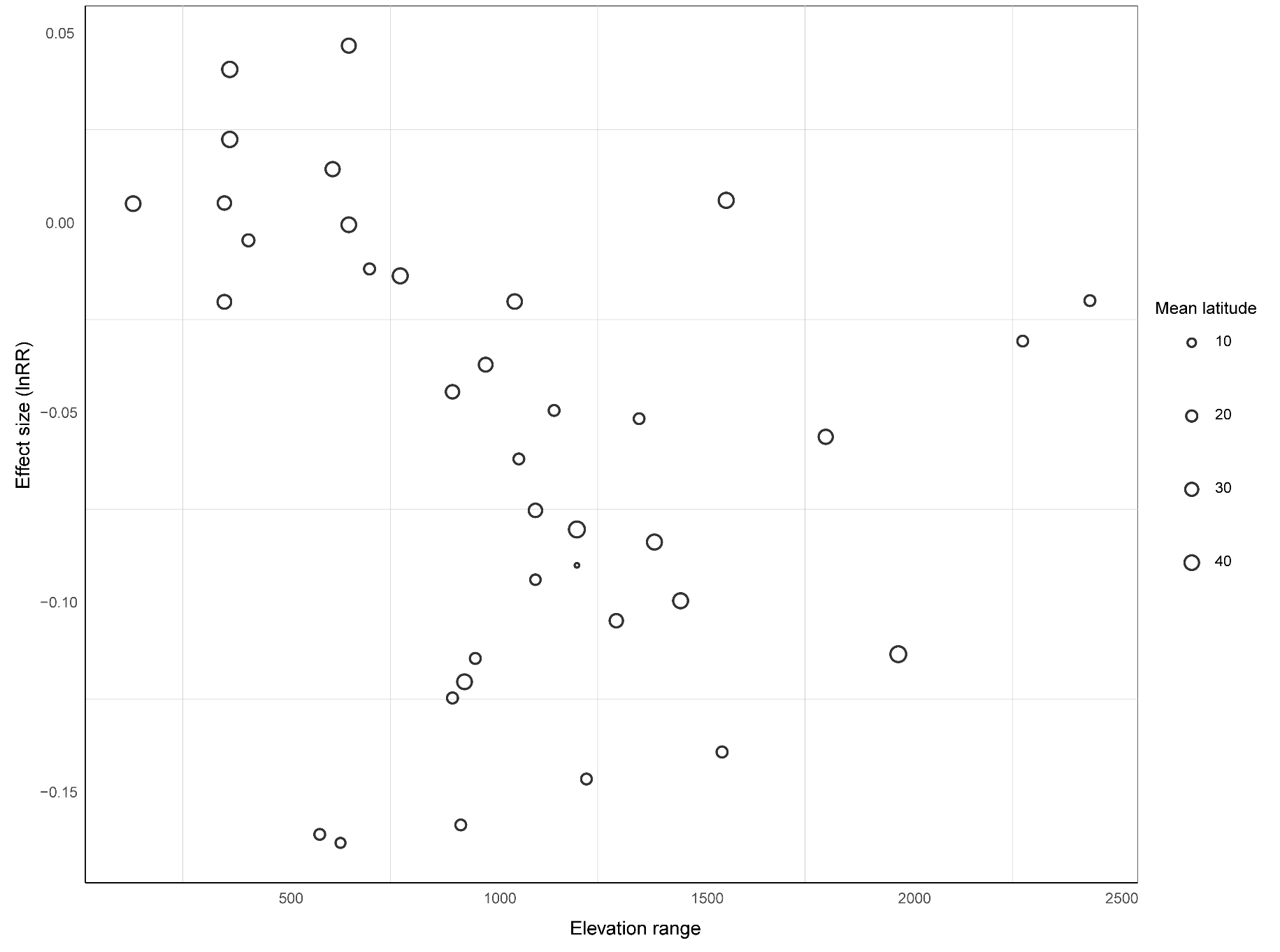


Figure S13 Scatterplot showing the relationship between body temperature difference of low-high elevation population pairs and mean latitude. Point size indicates the mean latitude of the population pairs ((in ° of latitude). Positive effect sizes indicate higher body temperature in high-elevation populations; negative values indicate higher body temperatures in low elevation populations


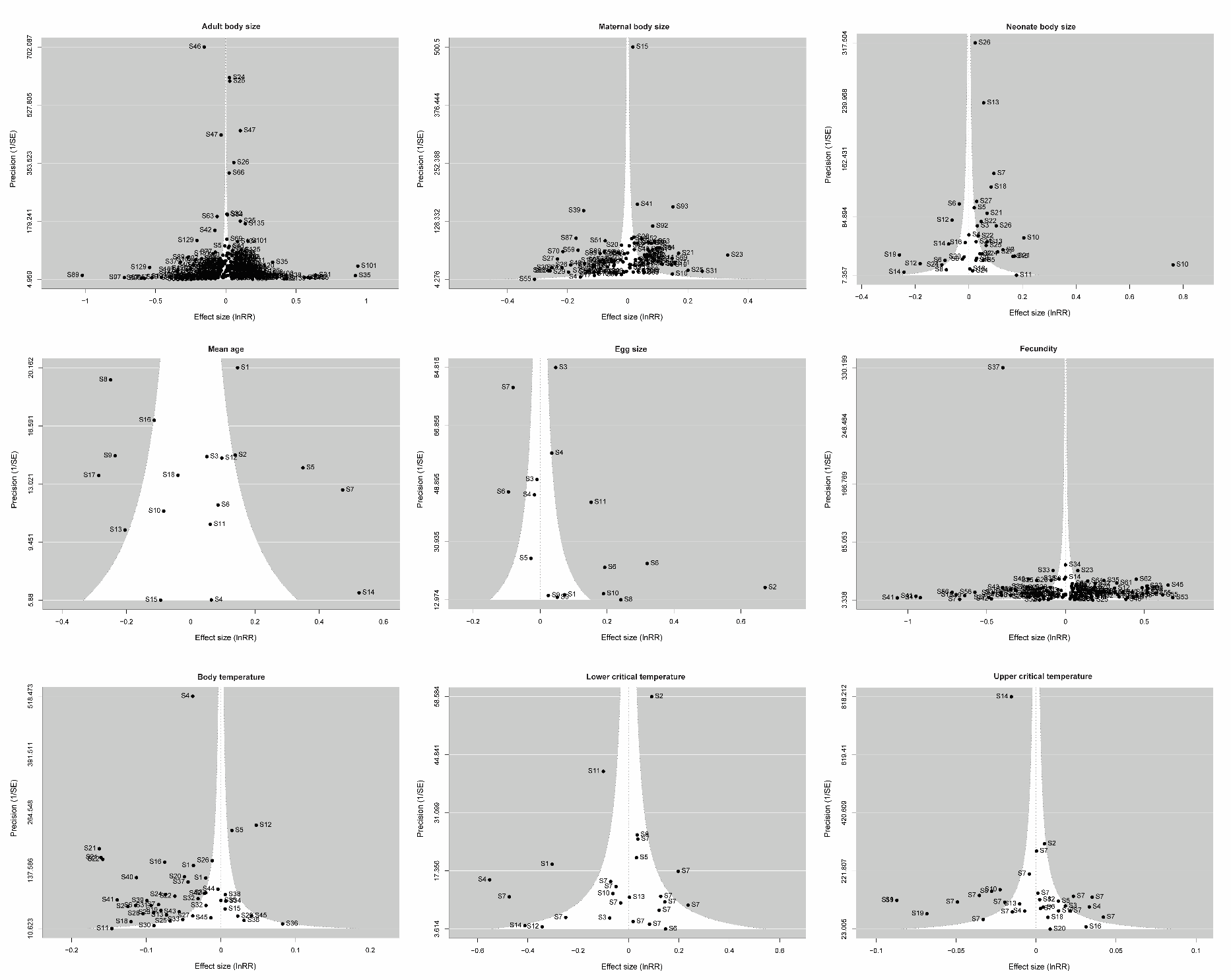


Figure S12 Exploratory funnel plots for mean effect sizes of traits (lnRR) included in the analysis (studyID used as label)


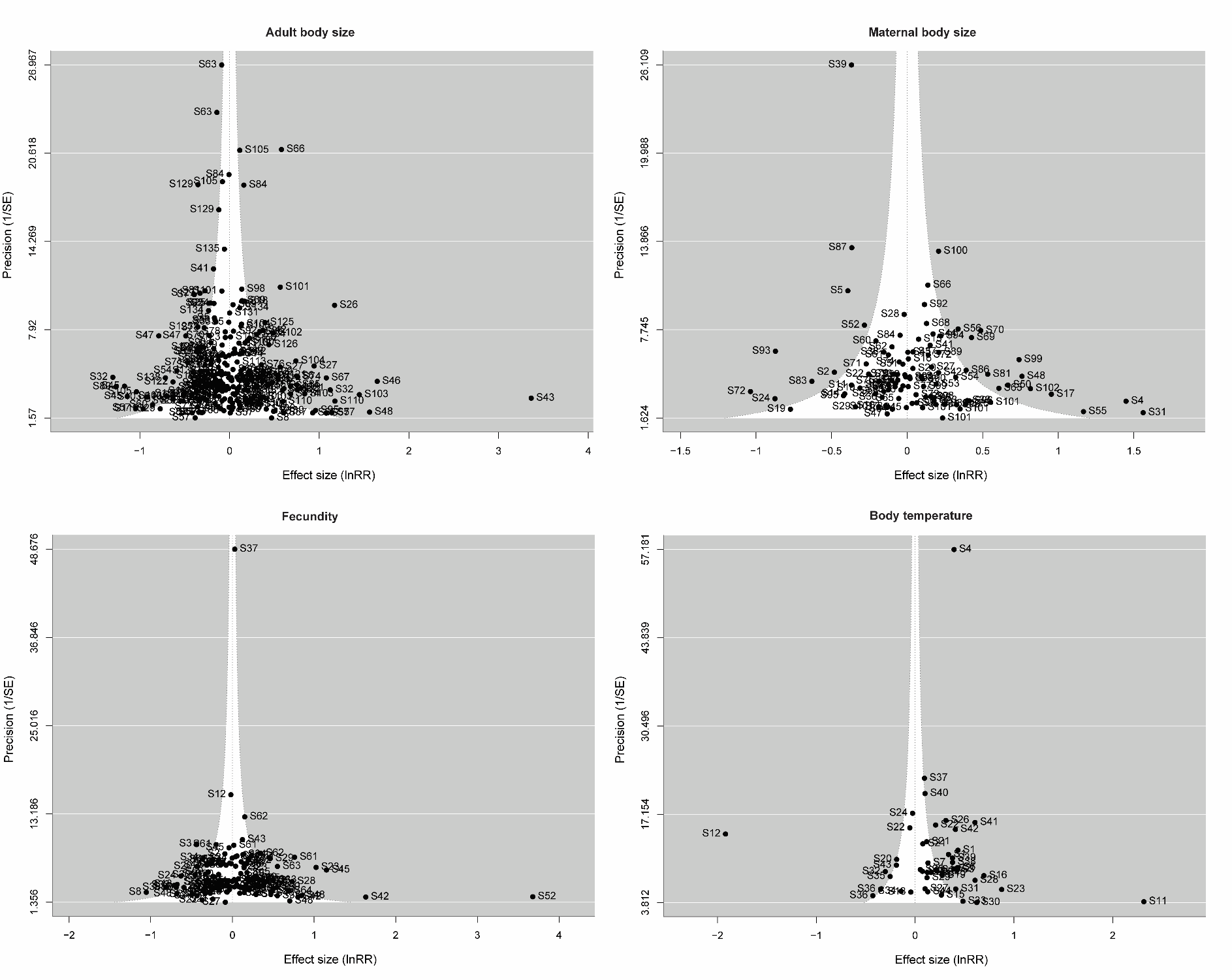


Figure S13 Exploratory funnel plots for the coefficient of variation of traits (CVR) included in the analysis (studyID used as label)

**Supplementary references**

1.

Cinar, O., Nakagawa, S. & Viechtbauer, W. (2022). Phylogenetic multilevel meta-analysis: A simulation study on the importance of modelling the phylogeny. *Methods in Ecology and Evolution*, 13, 383-395.

2.

Fu, R., Gartlehner, G., Grant, M., Shamliyan, T., Sedrakyan, A., Wilt, T.J. *et al.* (2011). Conducting quantitative synthesis when comparing medical interventions: AHRQ and the Effective Health Care Program. *Journal of Clinical Epidemiology*, 64, 1187-1197.

3.

Ghalambor, C.K., Huey, R.B., Martin, P.R., Tewksbury, J.J. & Wang, G. (2006). Are mountain passes higher in the tropics? Janzen's hypothesis revisited. *Integrative and Comparative Biology*, 46, 5-17.

4.

Higgins, J.P.T., T., L. & Deeks, J.J. (2024). Chapter 6: Choosing effect measures and computing estimates of effect [last updated August 2023]. In: *Cochrane Handbook for Systematic Reviews of Interventions version 6.5. Available from* [*www.training.cochrane.org/handbook*](www.training.cochrane.org/handbook) (eds. Higgins, JPT, Thomas, J, Chandler, J, Cumpston, M, Li, T, Page, MJ *et al.*). Cochrane.

5.

Higgins, J.P.T., Thompson, S.G., Deeks, J.J. & Altman, D.G. (2003). Measuring inconsistency in meta-analyses. *BMJ*, 327, 557-560.

6.

Hozo, S.P., Djulbegovic, B. & Hozo, I. (2005). Estimating the mean and variance from the median, range, and the size of a sample. *BMC Medical Research Methodology*, 5, 13.

7.

Janzen, D.H. (1967). Why Mountain Passes are Higher in the Tropics. *The American Naturalist*, 101, 233-249.

8.

Muñoz, M.M. & Bodensteiner, B.L. (2019). Janzen’s Hypothesis Meets the Bogert Effect: Connecting Climate Variation, Thermoregulatory Behavior, and Rates of Physiological Evolution. *Integrative Organismal Biology*, 1, 1-12.

9.

Nakagawa, S., Lagisz, M., Jennions, M.D., Koricheva, J., Noble, D.W.A., Parker, T.H. *et al.* (2022). Methods for testing publication bias in ecological and evolutionary meta-analyses. *Methods in Ecology and Evolution*, 13, 4-21.

10.

Nakagawa, S., Lagisz, M., O'Dea, R.E., Pottier, P., Rutkowska, J., Senior, A.M. *et al.* (2023a). orchaRd 2.0: An R package for visualising meta-analyses with orchard plots. *Methods in Ecology and Evolution*, 14, 2003-2010.

11.

Nakagawa, S., Yang, Y., Macartney, E.L., Spake, R. & Lagisz, M. (2023b). Quantitative evidence synthesis: a practical guide on meta-analysis, meta-regression, and publication bias tests for environmental sciences. *Environmental Evidence*, 12, 8.

12.

Noble, D.W.A., Lagisz, M., O'dea, R.E. & Nakagawa, S. (2017). Nonindependence and sensitivity analyses in ecological and evolutionary meta-analyses. *Molecular Ecology*, 26, 2410-2425.

13.

Page, R.D.M. & Holmes, E.C. (1998). Chapter 2 Trees. Molecular Evolution: A Phylogenetic Approach. Blackwell Science Oxford, UK, pp. 11-36.

14.

Paradis, E. & Schliep, K. (2018). ape 5.0: an environment for modern phylogenetics and evolutionary analyses in R. *Bioinformatics*, 35, 526-528.

15.

Stahel, W.A. (2002). *Statistische Datenanalyse. Eine Einführung für Naturwissenschaftler*. Vieweg, Braunschweig.

16.

Tonini, J.F.R., Beard, K.H., Ferreira, R.B., Jetz, W. & Pyron, R.A. (2016). Fully-sampled phylogenies of squamates reveal evolutionary patterns in threat status. *Biological Conservation*, 204, 23-31.

17.

Valentine, J.C., Pigott, T.D. & Rothstein, H.R. (2010). How Many Studies Do You Need?:A Primer on Statistical Power for Meta-Analysis. *Journal of Educational and Behavioral Statistics*, 35, 215-247.

18.

Viechtbauer, W. (2010). Conducting meta-analyses in R with the metafor package. *Journal of statistical software*, 36, 1-48.
